# Climate Change Drives the Distribution of Insect Vectors for GLRaV‐3 on a Global Scale

**DOI:** 10.1002/ece3.72297

**Published:** 2025-10-14

**Authors:** Minmin Niu, Yunyun Lu, Boxiang Zhao, Fengxia Dong, Junfei Bi, Pengfei Jing, Kangjie Wang, Zhengyuan Liu, Jiufeng Wei, Wei Ji

**Affiliations:** ^1^ College of Plant Protection Shanxi Agricultural University Taigu China; ^2^ College of Chemical Engineering Huaqiao University Quanzhou China; ^3^ College of Horticulture Shanxi Agricultural University Taigu China; ^4^ Shanxi Yuncheng Vocational and Technical College of Agricultural Yuncheng China; ^5^ Bureau of Agriculture and Rural Affairs of Yuncheng City Yanhu China

**Keywords:** climate change, GLRaV‐3, MaxEnt, soft‐scale insects, vector‐borne plant diseases

## Abstract

Grapevine leafroll‐associated virus 3 (GLRaV‐3) is a significant plant virus affecting grapevines worldwide, causing considerable economic losses. Soft scale insects (Coccidae) serve as key vectors for GLRaV‐3 transmission. Understanding how climate change impacts the distribution of these vector species is crucial for improving grapevine disease management strategies. Despite previous studies focusing on other insect vectors, limited research has been conducted on soft scale species, especially in the context of climate change. This study addresses the research gap by predicting the future global distribution of soft scale species responsible for GLRaV‐3 transmission under various climate change scenarios. The potential distribution of seven soft scale species was analyzed using the MaxEnt model. Data on species occurrence were gathered from global biodiversity databases, and key environmental variables were identified using principal component analysis. Climate projections were incorporated using Shared Socioeconomic Pathways (SSPs) under four future timeframes (2030s, 2050s, 2070s, 2090s). The model indicated that temperature plays a critical role in limiting soft scale distribution, with projections showing a northward shift in distribution for several species under climate change. Three species are expected to expand their range, while the remaining four may see a reduction in suitable habitat. These shifts suggest potential changes in GLRaV‐3 transmission risk in key grapevine‐growing regions. This research provides vital insights into the future distribution of GLRaV‐3 vectors, helping to guide targeted surveillance and management strategies. By predicting potential outbreak areas, this study contributes to the proactive management of grapevine diseases under changing climatic conditions.

## Graphical abstract

The distribution range of seven insect vectors of GLRaV‐3 were predicted by MaxEnt model. Thermal conditions were a vital factor constraining the potential distribution ranges of all vector insects. Centroid shifts suggested that the potential distribution range of soft scale will move northward under climate change. Our study provides implications for control the spread of GLRaV‐3.

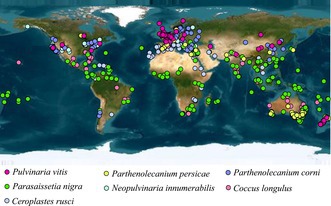

## 1 Introduction

Scale insects (Coccomorpha) are a remarkably diverse group, with over 8500 described species classified into 33 extant families worldwide (García Morales et al. 2016; Hou et al. 2023). Along with aphids, whiteflies, and psyllids, they constitute the suborder Sternorrhyncha (Drohojowska et al. 2020; Zhao et al. 2023). Due to their diverse and fascinating biology, scale insects have attracted scientific interest for nearly two centuries. They are not only significant agricultural and forestry pests (Ülgentürk and Canakcioğlu 2004; Miller and Davidson 2005; Miller et al. 2005), but also offer intriguing research avenues in areas like chromosome number (Nur et al. 1987; Gavrilov‐Zimin 2017), genetic systems (Gullan and Kosztarab 1997; Normark 2003), and endosymbiotic microorganisms (Ross et al. 2012; Gil et al. 2004). Adding another layer to their ecological significance, some scale insects act as vectors for plant viruses (Herrbach et al. 2016). While the number of species with this capability (around 35) is considerably lower than for aphids, it is important to note that this is despite the greater diversity of scale insects (approximately 8000 species) compared to aphids (around 4400) (García Morales et al. 2016; Mille et al. 2020; Kakoti et al. 2022). Among these vector species, only mealybugs (Pseudococcidae) and soft scales (Coccidae) within the superfamily Coccoidea have been confirmed to transmit viruses (Herrbach et al. 2016, 2017; Fuchs et al. 2009). While mealybugs can transmit viruses to a variety of crops, including cacao (Puig et al. 2021), grapevine (Tsai et al. 2010; Hommay et al. 2022a), pineapple (Carrasco‐Lozano et al. 2023), and cherry (Mekuria et al. 2013), soft scales have so far only been linked to grapevine virus transmission (Tsai et al. 2010; Hommay et al. 2022b).

Many factors hinder the sustainability and profitability of the grape industry, including a lack of certified planting materials (Naik et al. 2023), climate change (Reineke and Thiery 2016; Ponti et al. 2018), pests (Reineke and Thiery 2016), and diseases (Gramaje et al. 2018). Among these challenges, viruses pose a significant threat, particularly those that can severely impact grape quality and yield. Among a wide range of viruses and virus‐like agents affecting grapevines (Martelli 2014), grapevine leafroll disease (GLD) is considered a serious threat to wine grapes across many grapevine‐growing regions, especially GLRaV‐3 (Grapevine leafroll‐associated virus 3) (Naidu et al. 2014). GLRaV‐3 belongs to the genus *Ampelovirus*, family Closteroviridae, and is one of the most economically damaging viruses worldwide (Charles et al. 2009). It has colonized most grape‐growing regions through the exchange of infected plant material (Maree et al. 2013). So far, GLRaV‐3 is widely distributed in different regions, including Europe (Hanĉević et al. 2020), South and North America (Gouveia et al. 2011; Poojari et al. 2017), the Middle East (Anfoka et al. 2004), Northern and Southern Africa (Maree et al. 2008), Asia (Farooq et al. 2013; Hao et al. 2022), and Oceania (Chooi et al. 2022). It affects various aspects of grapevine health (Maree et al. 2013). GLRaV‐3 can cause significant economic losses and yield losses (20%–40%) to the wine, table grape, and raisin industries (Maree et al. 2013; Habili and Nutter Jr. 1997). It is estimated that infection by GLRaV‐3 alone may lead to an economic loss of between $25,000 and $41,000 per hectare over the lifespan of a vineyard (Naidu et al. 2014; Atallah et al. 2012). In the North Coast of California alone, the economic benefits of GLRaV‐3 certified virus‐free seedlings are estimated at $53.5 million annually (Fuller et al. 2019).

To control GLRaV‐3 spread, vineyard managers often adopt a zero‐tolerance approach to insect vectors, typically monitored by presence/absence scores (Alimeida et al. 2013). However, the incidence of insect‐borne plant diseases is directly affected by the distribution and abundance of their vectors. Controlling these vectors could significantly reduce GLRaV‐3 spread. While all soft scale and mealybug species on vines are considered potential vectors (Alimeida et al. 2013), previous studies have only focused on mealybugs under climate change (Wei et al. 2024). The ecological relevance of different soft scale species to GLRaV‐3 remains unclear. Traditional monitoring of soft scale populations is labor‐intensive and expensive, especially at low densities. This necessitates exploring alternative methods to aid vineyard managers in managing these GLRaV‐3 vectors.

Species distribution modeling (SDM) has been used to simulate suitable areas for many taxa, including invasive pests (Chapman et al. 2019) and invasive plants (Jones 2012). A number of algorithms have been developed to assess suitable areas for species, such as maximum entropy (MaxEnt) modeling (Karuppaiah et al. 2023), Climex (Narouei‐Khandan et al. 2022), GARP (Yang et al. 2022), and Biomod2 (Huang et al. 2023). MaxEnt is a machine‐learning model with broad applications in ecology and sustainability research. This software has advantages over other models, including a short model run time, easy operation, small sample size requirements, and outstanding ability to predict suitable areas based on incomplete data sets (Elith et al. 2006; Bean et al. 2012; Kramer‐Schadt et al. 2013). This method has been used to predict the range of insect‐borne plant pathogens based on the predicted potential distribution of insect vectors, including the pathogens *Xylella fastidiosa* (Godefroid et al. 2019; Rossi and Rasplus 2023; Yoon and Lee 2023), *Sphaeropsis sapinea* (Bosso et al. 2017), and *Candidatus Liberibacter asiaticus* (Narouei‐Khandan et al. 2015; Aidoo et al. 2022).

Crucially, the suitability of various soft scale species as GLRaV‐3 vectors remains unevaluated. Furthermore, climate change poses a complex threat, potentially influencing vector distribution and abundance through both direct and indirect pathways (Baylis 2017; Ogden and Lindsay 2018). As a well‐documented consequence of global warming, a rise in insect‐borne plant diseases is expected due to expanding vector ranges and rapid insect reproduction (Skendžic et al. 2021).

The current study is focused on soft scale, a widespread vector of the virus complex including GLRaV‐3. Eight soft scales, *Ceroplastes rusci* (CR), *Coccus longulus* (CL), *Neopulvinaria innumerabilis* (NI), *Parasaissetia nigra* (Nietner, 1861) (PN), *Parthenolecanium corni* (PC), *Parthenolecanium persicae* (PP), *Pulvinaria vitis* (PV), and *Saissetia* sp., are known to transmit GLRaV‐3 (Herrbach et al. 2017; Tsai et al. 2010). *Saissetia* sp. was not included in the current study owing to a lack of detailed information on the species. The major goals of the current study were as follows: (1) Evaluate trends in suitable habitat for GLRaV‐3 insect vectors under climate change scenarios. MaxEnt, a machine learning technique, was employed to predict these potential future distributions; (2) Identify key climatic factors limiting the potential distribution of insect vectors. Understanding these factors is crucial for predicting future spread; and (3) Provide a theoretical framework for soft scale vector management and control policies. This framework can inform decision‐making to mitigate GLRaV‐3 spread.

## 2 Material and Methods

### 2.1 Occurrence Data

**FIGURE 1:**
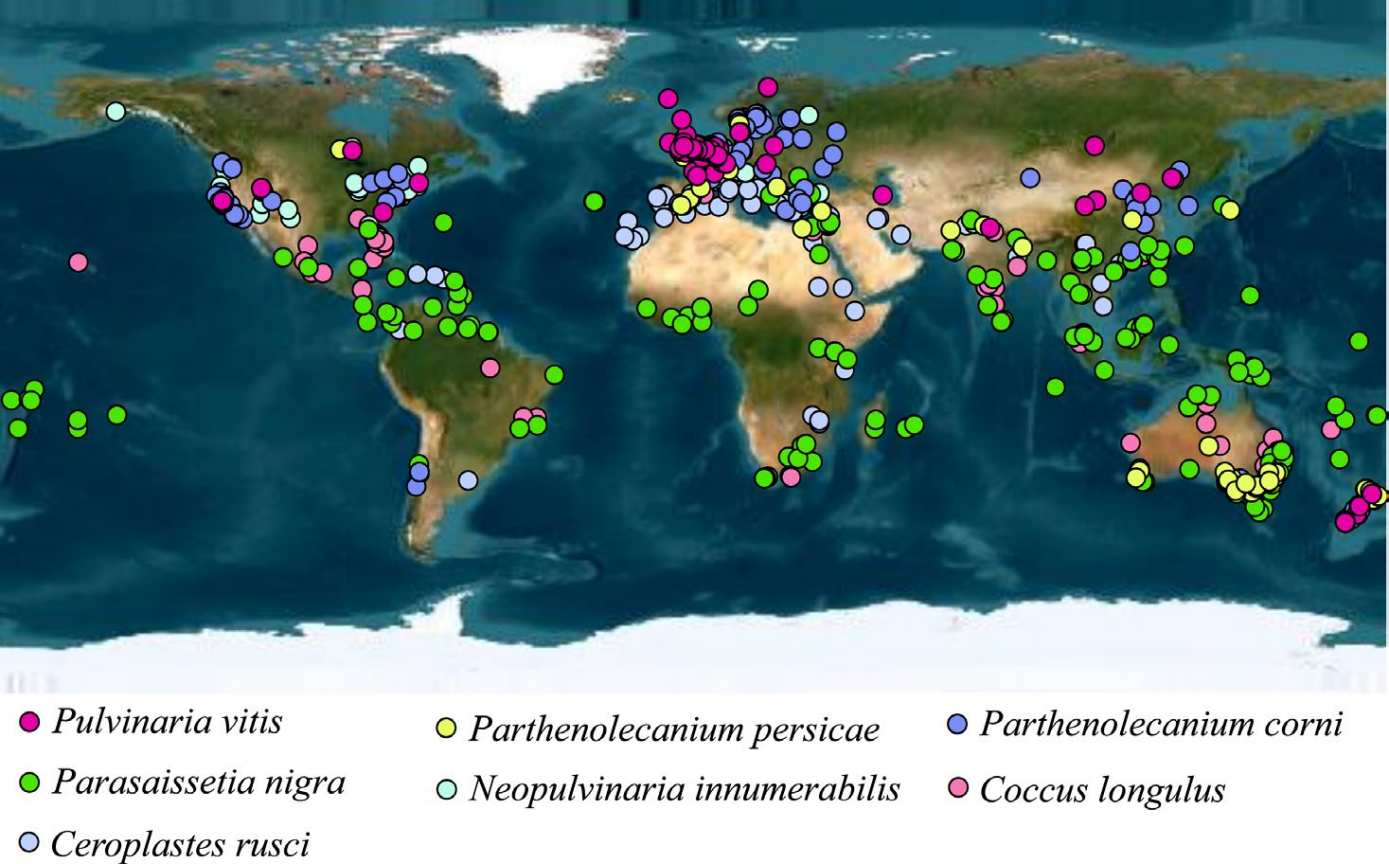
Occurrence location of seven insect vectors used in current study.

Most occurrence data for soft scale vectors of GLRaV‐3 were obtained from the Global Biodiversity Information Facility (GBIF) (https://www.gbif.org/): CR (Doi: 10.15468/dl.sugw6x), CL (Doi: 10.15468/dl.mmqxk9), NI (Doi: 10.15468/dl.mmqxk9), PC (Doi: 10.15468/dl.kzndyd), PN (Doi: 10.15468/dl.kzndyd), PP (Doi: 10.15468/dl.eke3qg), and PV (Doi: 10.15468/dl.tuyf94). Additional data were obtained from ScaleNet (https://scalenet.info/). Coordinate data for each location were obtained from the database or using the Google Earth platform. Duplicate samples and minor location errors in distribution data were removed manually. The initial data for soft scale were as follows: 419 georeferenced occurrences for CR; 498 for CL; 62 for NI; 1599 for PN; 870 for PC; 300 for PP; and 661 for PV. To reduce sampling bias, data thinning was implemented using the spThin package (Aiello‐Lammens et al. 2015) version 0.2.0 in R with a minimum separation distance of 5 km between sites. After this step, 146 occurrence records for CR, 81 for CL, 42 for NI, 236 for PN, 139 for PC, 64 for PP, and 89 for PV were retained to construct the final model. All occurrence data are shown in Figure 1 and Table S1.

### 2.2 Screening and Processing of Environmental Variables

The main bioclimatic variables (bio1–bio10) and altitude data were downloaded from the WorldClim Global Climate Database (version 2.1; https://worldclim.org/) (Fick and Hijmans 2017) for modeling potentially suitable areas with a spatial resolution of 2.5 arc‐minutes (approximately 5 km × 5 km). The WorldClim database is widely used to simulate the potential distribution of species, including pests (Maruthadurai et al. 2023) and plants (Yan et al. 2021). This database offers high‐resolution climate data (temperature, precipitation, seasonality) across the globe, making it suitable for studying species distribution at large scales (Choudhury et al. 2016).

Multicollinearity arises when two or more explanatory variables in a statistical model are highly correlated with each other. This strong correlation creates redundancy in the information they provide. As a consequence, the model coefficients (estimates of how each variable affects the outcome) become unreliable and their standard errors inflate (Júnior and Nóbrega 2018). This leads to overfitting and affects the accuracy of prediction (Santana Jr et al. 2019). To address multicollinearity among the bioclimatic variables, Principal Component Analysis (PCA) was employed, as recommended by Govere et al. (2020) and Hanspach et al. (2011). Using IBM SPSS Statistics 25, PCA was conducted to identify a set of representative environmental variables with minimal redundancy. The variables contributing most to the variance were retained for the final model (Table S2). PCA identified six principal components for all soft scale species. However, components 5 and 6 explained the same variable, so only the first five environmental variables were retained for the CR model (details in Table 1). Similarly, PCA for PC soft scales yielded four unique components, resulting in the selection of four environmental variables for further analysis (Table 1).

**TABLE 1:** Environmental variables used by seven insect vectors in the current study.

Code	Description	CR	CI	NI	PN	PP	PV	PC
alt	Altitude		●		●	●	●	●
Bio1	Annual mean temperature						●	
Bio2	Mean diurnal range (Mean of monthly [max temp–min temp])						●	
Bio3	Isothermality (BIO2/BIO7) (*×*100)						●	
Bio4	Temperature Seasonality (standard deviation *×* 100)		●					●
Bio5	Max temperature of warmest month	●	●		●	●		●
Bio6	Min temperature of coldest month		●		●	●		
Bio7	Temperature annual range (Bio5‐Bio6)							
Bio8	Mean temperature of wettest quarter			●				
Bio9	Mean temperature of driest quarter	●						
Bio10	Mean temperature of warmest quarter			●				
Bio11	Mean temperature of coldest quarter			●				
Bio12	Annual precipitation	●		●				
Bio13	Precipitation of wettest month							●
Bio14	Precipitation of driest month							
Bio15	Precipitation seasonality (Coefficient of variation)	●	●	●		●		
Bio16	Precipitation of wettest quarter						●	
Bio17	Precipitation of driest quarter							
Bio18	Precipitation of warmest quarter				●	●		
Bio19	Precipitation of coldest quarter	●				●		

*Note:* Black dot (●) represents the environmental variables used in the current study.

Abbreviations: CL, *Coccus longulus* ; CR, *Ceroplastes rusci*; NI, *Neopulvinaria innumerabilis*; PC, *Parthenolecanium corni*; PN, *Parasaissetia nigra* ; PP, *Parthenolecanium persicae*; PV, *Pulvinaria vitis*.

### 2.3 Future Climate Variables

Climate data were obtained from WorldClim version 2.1 (https://worldclim.org/) (Fick and Hijmans 2017) to project future distributions. This data incorporates Shared Socioeconomic Pathways (SSPs), a set of scenarios that account for different socioeconomic developments and their influence on greenhouse gas emissions (Moss et al. 2010; Riahi et al. 2017). Four SSPs were specifically focused on: SSP126, SSP245, SSP370, and SSP585. SSP126 represents a future with the lowest greenhouse gas concentrations, while SSP585 reflects a scenario with the most significant radiative forcing (Tian et al. 2021; Zhang et al. 2022). To account for uncertainty in future climate projections, three randomly selected Global Circulation Models (GCMs) from the Coupled Model Intercomparison Project Phase 6 (CMIP6) of the Intergovernmental Panel on Climate Change (IPCC) were employed. These models were BCC‐CSM2‐MR, MIROC6, and CanESM5. To assess the potential impact of climate change on insect vector distribution, two Shared Socioeconomic Pathways (SSPs) combined with four future time periods were considered. The SSPs were SSP126 (low greenhouse gas emissions) and SSP585 (high greenhouse gas emissions). The time periods were: 2030s (average for 2021–2040), 2050s (average for 2041–2060), 2070s (average for 2061–2080), and 2090s (average for 2081–2100).

### 2.4 Modeling Process

MaxEnt (version 3.4.1) was employed to estimate the dynamics of the potential distribution of soft scales. Transferability is a key challenge in ecological niche modeling (Low et al. 2021). Models that accurately predict in one context may not perform well in another, potentially leading to misleading results (Merow et al. 2013). To address potential limitations arising from species traits that could hinder model transferability (Low et al. 2021), feature selection was employed during model development. Additionally, key parameters within MaxEnt were optimized (Low et al. 2021; Merow et al. 2013) to achieve the best possible model complexity and avoid overfitting. This optimization process involved evaluating different Feature Combination (FC) settings and the Regularization Multiplier (RM). To prevent overfitting and improve model accuracy, the R package “ENMeval 2.0.4” (Muscarella et al. 2014) was applied to optimize MaxEnt parameters. An ideal balance between model complexity and overfitting was identified using ENMeval by evaluating eight different Feature Combination (FC) options (combinations of linear, quadratic, product, threshold, and hinge functions) alongside Regularization Multiplier (RM) values ranging from 0.5 to 4. The “checkerboard2” method within ENMeval (Warren and Seifert 2011) was used to calculate AICc scores, and models with the lowest delta AICc scores were chosen for final analysis. The ENMeval results are shown in Figure S1 and Table S3. The final FC and RM values are shown in Table 2. Other parameters for MaxEnt were set as follows: the relative influence of each environmental variable using the jackknife procedure; model outputs were generated in the logistic format for all analyses; 10‐fold cross‐validation was employed; the maximum pseudo‐absence points was set to 10,000; finally, a “fade‐by‐clamping” parameter was used to prevent model predictions outside the observed environmental range of the soft scale species. A 10th percentile training presence logistic threshold was used to define the binary suitable/non‐suitable habitats, which is widely used for species distribution modeling, particularly when datasets are collected over time by different observers and methods (Bosso et al. 2016; Wang et al. 2011). The final potential distribution maps for each insect vector were categorized into four suitability levels: Unsuitable habitat: Areas below a threshold value (based on the 10th percentile of training presence data) were deemed unsuitable for the insect; Low suitability: Areas with values between the threshold and 0.4 indicated low habitat suitability; Moderate suitability: Values between 0.4 and 0.6 indicated areas with moderate suitability; High suitability: Areas with values between 0.6 and 1 were classified as having high habitat suitability for the insect vector.

**TABLE 2:** Selected feature combination (FC) and regularization multiplier (RM) for each soft scale used in current study.

No.	Name	Abbreviation	FC	RM
1	*Ceroplastes rusci*	CR	LQHP	0.5
2	*Coccus longulus*	CL	QHPT	2
3	*Neopulvinaria innumerabilis*	NI	LQH	1
4	*Parasaissetia nigra*	PN	LQHP	0.5
5	*Parthenolecanium corni*	PC	LQHP	0.5
6	*Parthenolecanium persicae*	PP	LQHPT	1.5
7	*Pulvinaria vitis*	PV	LQHP	0.5

To account for variation among the three Global Circulation Models (GCMs) used, a final suitability map for future climatic conditions was generated by averaging the individual model outputs. SDMtoolbox, a Python‐based GIS toolkit (Brown et al. 2017), was then employed to combine the current and future suitability maps (converted to binary format). Through this approach, areas of habitat change for the insect vectors were identified and categorized as habitat contraction, expansion, or stable range. To quantify distribution shifts under current and future climate change scenarios, changes in the centroid position of suitable habitat areas were analyzed. This involved calculating the movement of the center point for both the total suitable area and the area with high habitat suitability (as defined by your classification scheme). These shifts were assessed based on longitude and latitude coordinates, following the approach used by Yan et al. (2021), thereby allowing the determination of the direction and distance of potential habitat migration for the insect vectors. The centroid changes over time for each species were generated using SDMtoolbox (Brown et al. 2017).

### 2.5 Estimation of Model Performance for Insect Vectors

Various methods were used to assess the performance of SDM, such as the area under the receiver operating characteristic curve (AUC) (Fielding and Bell 1997) and true skill statistic (TSS) (Allouche et al. 2006). AUC is a widely used method where values above 0.5 indicate a model performing better than random chance. However, AUC limitations include not considering the spatial distribution of errors and giving equal weight to two types of errors (omission and commission) (Wei et al. 2017, 2020; Lobo et al. 2008). Thus, TSS was also employed to assess the performance of MaxEnt in the current study (Allouche et al. 2006; Wunderlich et al. 2019). This method addresses the limitations of AUC by incorporating both omission and commission errors. TSS values range from −1 to 1, with 1 indicating perfect model performance (Bosso et al. 2016; Gallien et al. 2012; Hattab et al. 2017).

## 3 Results

### 3.1 Model Performance for Each Scale Insect

Strong predictive ability of the MaxEnt models was demonstrated by both AUC and TSS metrics (Table 3). All insect species models achieved AUC values between 0.934 and 0.968, and TSS values between 0.749 and 0.871. These values significantly surpass those expected from random chance, indicating high model accuracy. This confirms that the MaxEnt models were well configured, reliable, and suitable for further analyses aimed at predicting future insect vector distributions.

**TABLE 3:** Model performance of each vector insect by the area under the receiver operating characteristic curve (AUC) and true skill statistic (TSS).

No.	Species name	Abbreviation	AUC	TSS
1	*Ceroplastes rusci*	CR	0.942	0.8711
2	*Coccus longulus*	CL	0.934	0.7805
3	*Neopulvinaria innumerabilis*	NI	0.964	0.7815
4	*Parasaissetia nigra*	PN	0.935	0.7496
5	*Parthenolecanium corni*	PC	0.960	0.8356
6	*Parthenolecanium persicae*	PP	0.953	0.8374
7	*Pulvinaria vitis*	PV	0.968	0.7994

### 3.2 Environmental Variables That Define the Model

Temperature was found to play a critical role in shaping the potential distribution of all seven soft scale species (Table 4). Here's a breakdown of the key factors for each species: CL, PN, and PP: Minimum temperature of the coldest month (Bio6) was the most influential factor, contributing over 50% to their potential distribution range. CR: Mean temperature of the driest quarter (Bio9) had the strongest influence on its distribution. NI: Mean temperature of the coldest quarter (Bio11) had the greatest impact, contributing about 46.4%. PC: Temperature seasonality (Bio4) emerged as the most important factor, affecting its potential distribution by 37.7%. PV: Annual mean temperature (Bio1) was the primary determinant for its distribution, contributing 43.3%. These findings highlight the varying temperature sensitivities of different soft scale species. Understanding these climatic drivers is crucial for predicting their potential spread under future climate change scenarios.

**TABLE 4:** Percent contribution of environmental variables of each insect vector.

No.	Species name	Abbreviation	Percent contribution of climatic variable
1	*Ceroplastes rusci*	CR	**Bio9: 78.6%**; Bio19: 24.3%; Bio12: 11.2%; Bio5: 8.5%; Bio15: 7.4%
2	*Coccus longulus*	CL	**Bio6: 50.9%**; Alt: 24.4%; Bio4: 17.2%; Bio5: 5.2%; Bio15: 2.4%
3	*Neopulvinaria innumerabilis*	NI	**Bio11: 46.4%**; Bio15: 31.7%; Bio12: 12.1%; Bio10: 9.6%; Bio8: 0.2%
4	*Parasaissetia nigra*	PN	**Bio6: 62.5%**; Alt: 14%; Bio5: 12.8%; Bio18: 9%; Bio2: 1.8%
5	*Parthenolecanium corni*	PC	**Bio4: 37.7%**; Bio5: 34.3%; Alt: 14.3%; Bio13: 13.8%
6	*Parthenolecanium persicae*	PP	**Bio6: 61.8%**; Bio19: 26.7%; Bio5: 6.8%; Bio18: 3.8%; Bio15: 0.8%
7	*Pulvinaria vitis*	PV	**Bio1: 43.3%**; Bio3: 21.8%; Bio2: 15.5%; Bio16: 13.3%; Bio4: 6.1%

*Note:* The most important environmental variables that influence the model were marked in bold.

### 3.3 Response of Potential Habitat Suitability to Environmental Variables

Response curves illustrated the quantitative relationships between the logistic occurrence probability and environmental variables. The response curves (shown in Figure S2) offer valuable insights into the environmental preferences of each soft scale species. These curves illustrate the relationship between the probability of a species occurring in a particular location and various environmental variables. The response curves for seven soft scales are illustrated in Figure S2. Based on the response curves, high habitat suitability (> 0.6) for CR (Figure S2A) was found in areas where Bio5 was 5°C to 35°C, Bio9 was 15°C to 30°C, Bio12 was 700–800 mm, Bio15 was 48–100 mm, and Bio19 was 200–500 mm. For CL (Figure S2B), a high probability of occurrence was found in regions where Alt was below approximately 150 m, Bio 4 was 30°C to 48°C, Bio 5 was −3°C to 31°C, Bio 6 was 9°C to 24°C, Bio15 was 20–600 mm. Bio8 of −20°C to 20°C, Bio 4 of 2°C to 3°C, Bio11 of −2°C to 5°C, Bio12 of approximately 700–1100 mm, and Bio15 of below 40 mm were suitable for NI (Figure S2C).

Based on response curves, high habitat suitability for PC was observed for Alt of below 800 mm, Bio4 of approximately 4°C to 8°C, Bio5 of approximately 20°C to 30°C, and Bio13 of approximately 100–200 mm (Figure S2D). A high probability of PN occurrence was found in regions with Alt below 200 m, Bio2 of 5°C to 5.5°C, Bio3 of approximately 2.2–2.8, and Bio18 of approximately 900–1800 mm (Figure S2E).

The response curves for the PP distribution model (Figure S2F) suggested a high probability of occurrence in regions with Bio5 of approximately 20°C to 32°C, Bio6 of approximately 0°C to 10°C, Bio15 of below 250 mm, Bio18 of below 350 mm, and Bio19 of approximately 100–1100 mm. For PV (Figure S2G), a high probability of occurrence was found in regions with Bio1 at approximately 10°C to 12°C, Bio2 at approximately −1°C to 9°C, Bio3 at approximately 3 to 4.5, Bio4 at approximately 250–700 mm, and Bio16 at approximately 150–300 mm.

### 3.4 Potential Habitat Suitability of Soft Scale Under Current Conditions

**FIGURE 2:**
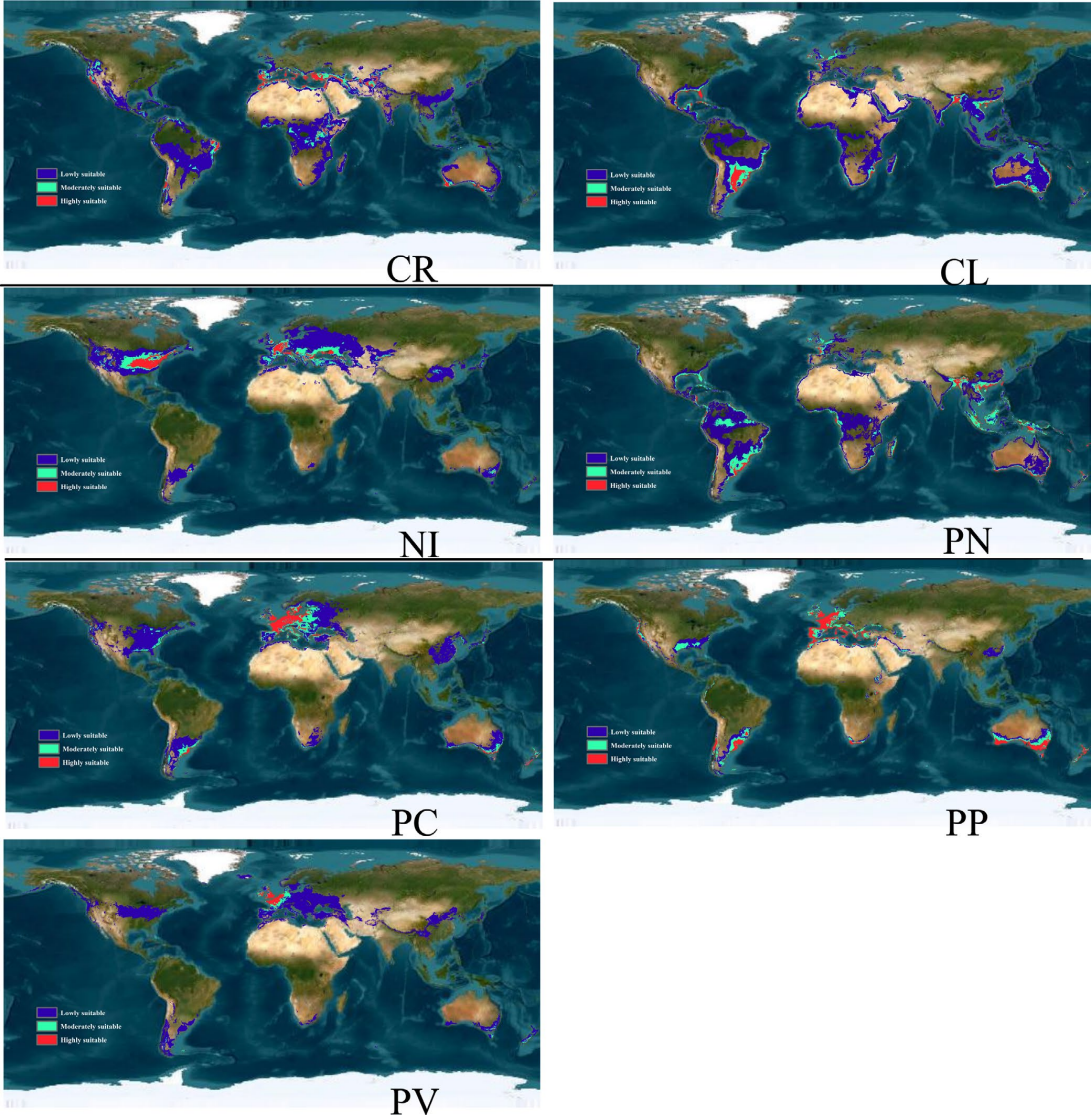
Potential distribution region of seven insect vectors under current study. CL, *Coccus longulus* ; CR, *Ceroplastes rusci*; NI, *Neopulvinaria innumerabilis*; PC, *Parthenolecanium corni*; PN, *Parasaissetia nigra* ; PP, *Parthenolecanium persicae*; PV, *Pulvinaria vitis*. Enlarged versions of the individual images depicted in the figure are provided in the Figure S4.

Several regions with high habitat suitability for the CR insect vector were identified under current climatic conditions (Figure 2, Table S4). These regions span across multiple continents: Americas: West coast of North America, western Mexico, Central America, parts of Central and South America (including eastern Brazil, Chile, and southern Argentina); Europe and Asia: Circum‐Mediterranean coast, central Asia, southern India, parts of Southeast Asia, and southern China; Africa: Central and southeastern Africa, and southeastern Madagascar; Australia: Southern, eastern, and western edges. The total estimated suitable area for CR under current conditions is approximately 4.1 × 10^7 km^2^.

For CL, part of the west coast of North America, Gulf Coast, Caribbean coast; most of South America; the coast of Africa, Madagascar; circum‐Mediterranean coast, Great Britain and Ireland, Red Sea coast, Eastern India, Southeast Asia, South China; New Zealand, and most of Australia and Papua New Guinea were identified, covering approximately 4.54 × 10^7^ km^2^ (Figure 2 and Table S4).

NI is another important vector, having a potential distribution spanning central North America; south‐central Argentina; most of Europe; Central Asia, Central China, South Korea, and Japan; south‐central Australia. This region covered approximately 3.52 × 10^7^ km^2^ (Figure 2 and Table S4). The potential distribution of PN was larger than that of NI, covering nearly 4.32 × 10^7^ km^2^ of the land mass in the world (Figure 2 and Table S4), mainly located on the west coast of North America, Gulf Coast, Central America; around the coast of South America, a large area of southeastern South America, northern South America; northeastern Europe, circum‐Mediterranean coast; around the coast of Africa, central Africa; around the Indian mainland, border of Nepal, Bhutan, and Bangladesh, Southeast Asia, South China, Japan; Papua New Guinea, New Zealand, and around the coast of Australia.

Two species (PC and PP) belonging to *Parthenolecanium* had similar potential distributions. In North America, the potential distribution included the west coast and southern USA. However, the potential range of PC was larger than that of PP. In South America, the potential distribution range of PP was located in Argentina, Uruguay, and Southern Chile, and that of PC was similar but larger. In Europe, the potential distribution range of PP included the circum‐Mediterranean coast and Northeastern Europe; the potential distribution of PC was similar to that of PP but extended to the east. In Africa, the two species showed a similar distribution, located in Southern Africa; however, the distribution range was larger for PP than PC. They showed a similar distribution pattern in Australia and Asia. The potential distribution region of PC covered approximately 4.32 × 10^7^ km^2^ of the land mass of the world (Figure 2 and Table S4), while that of PP covered approximately 1.78 × 10^7^ km^2^ (nearly 40% of the land mass covered by PP) (Figure 2 and Table S4).

High habitat suitability for the PV insect vector was identified across approximately 2.25 × 10^7^ km^2^ (Figure 2, Table S4). These regions are spread across several continents: Central North America; *South America*: Southern Chile and Southeast Argentina; *Europe*: Southern Europe; *Africa*: a small part of southern Africa; *Asia*: Central China and Nepal; *Oceania*: New Zealand and southern Australia.

### 3.5 Future Distribution Shifts Under Climate Change

**FIGURE 3:**
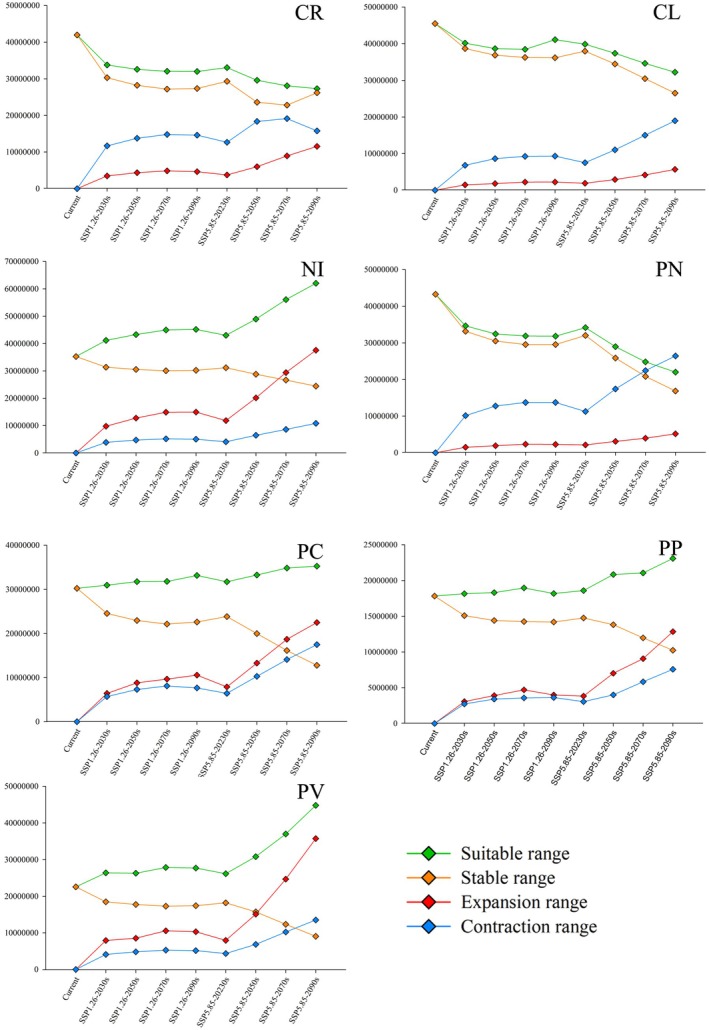
Change trend of potential distribution region of seven insect vectors under different climatic conditions. CL, *Coccus longulus* ; CR, *Ceroplastes rusci*; NI, *Neopulvinaria innumerabilis*; PC, *Parthenolecanium corni*; PN, *Parasaissetia nigra* ; PP, *Parthenolecanium persicae*; PV, *Pulvinaria vitis*. Enlarged versions of the individual images depicted in the figure are provided in the Figure S5.

To assess potential changes in soft scale distribution under future climate scenarios, three categories were employed: stable, expansion, and contraction. A shrinking potential distribution range for the CR, CL, and PN insect vectors was predicted under future climate scenarios (2030–2090), regardless of the emissions pathway (low—SSP1.26 or high—SSP5.85) (Figures 3, S3 and Table S4). This suggests that climate change may render currently suitable habitats less favorable for these species. Conversely, the areas of potential habitat expansion and contraction are predicted to increase. In contrast, the four remaining insect vectors (NI, PC, PP, and PV) exhibited an opposing trend. Their potential distribution ranges are predicted to increase under both low and high emission scenarios (Figures 3, S3, Table S4). This indicates that climate change might create new areas suitable for these species. These findings highlight the varied responses of different insect vectors to future climate change. Some species may experience range contractions, while others may expand their distributions. Understanding these variations is crucial for predicting future pest outbreaks and implementing effective management strategies.

### 3.6 Dynamics of Insect Vectors Under Climatic Change

**FIGURE 4:**
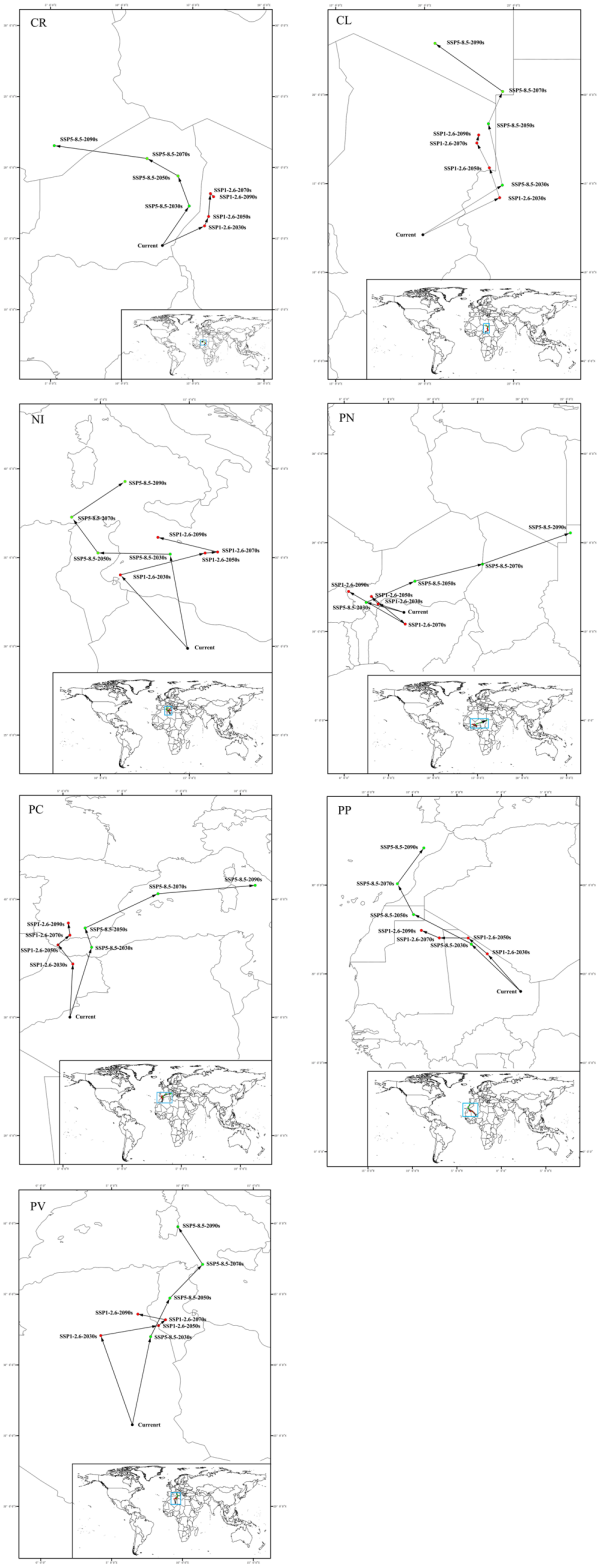
Centroid shifts of the potential distribution region of seven insect vectors under future climatic conditions. CL, *Coccus longulus* ; CR, *Ceroplastes rusci*; NI, *Neopulvinaria innumerabilis*; PN, *Parasaissetia nigra* ; PC, *Parthenolecanium corni*; PP, *Parthenolecanium persicae*; PV, *Pulvinaria vitis*. Enlarged versions of the individual images depicted in the figure are provided in the Figure S6.

Significant shifts in the future distribution of all soft scale species were indicated by population centroid analyses (Figure 4). This suggests that climate change will likely cause these insects to migrate long distances from their current locations. The extent of this migration appears to be influenced by the severity of climate change scenarios. Under the higher emission scenario (SSP585), all insect vectors are predicted to move further compared to the lower emission scenario (SSP1.26). Here's a breakdown of the predicted migration directions for each species: CR and CL: These species are expected to shift northward (SSP126) or northwestward (SSP585); NI, PP, and PN: Similar to CR and CL, these species are predicted to move northwestward (SSP126) or northeastward (SSP585); PC: This species will likely migrate northward (SSP126) or northeastward (SSP585); PV: This insect vector is predicted to shift northwestward under both climate scenarios. These findings highlight the potential for long‐distance migrations of soft scale insects due to climate change. Understanding these shifts is crucial for predicting future outbreaks and developing effective management strategies.

## 4 Discussion

### 4.1 Influence of Environmental Variables

Building on existing knowledge (Camacho and Chong 2015), temperature is reaffirmed as a critical factor limiting the distribution and abundance of soft scale insects. Among various environmental variables analyzed, thermal conditions emerged as the most significant determinant of their potential distribution ranges. This finding is consistent with the established understanding that temperature directly influences insect survival and reproduction (Frank and Just 2020). Similar to recent studies on other scale insects (Shan et al. 2022), temperature was highlighted as the primary factor shaping the global distribution of seven soft scale species. Specifically, the destructive pest *Ceroplastes cirripediformis* was significantly influenced by temperature, reinforcing the notion that thermal conditions are paramount in determining the geographic range of these insects (Wei et al. 2020; Wang et al. 2021).

Temperature was highlighted as the key factor shaping the distribution of soft scale insects. However, precipitation seasonality (Bio15) also emerged as an influential factor, corroborating findings from previous studies (Shan et al. 2022; Wang et al. 2021). Response curves indicated a negative correlation between Bio15 and the potential distribution ranges of *Coccus longulus* (CL), *Neopulvinaria innumerabilis* (NI), and *Parthenolecanium persicae* (PP). This suggests that seasonal rainfall patterns may indirectly affect the distribution of these pest species, potentially through impacts on crawler activity and population abundance, as suggested by Zhao et al. (2024).

### 4.2 Dynamics of Soft Scales Under Climatic Change

Consistency with previous research on global warming's effects on species distribution was observed (Freeman et al. 2018; Ma et al. 2022), and significant shifts in the future distribution of soft scale insects were predicted. For over half (57.1%) of the species modeled, the suitable habitat range is expected to expand under climate change scenarios where expansion outweighs contraction. Conversely, the remaining three species are projected to experience a decrease in suitable habitat due to dominant contraction. This highlights the varied responses of different soft scale species to climate change, with some potentially benefiting from new habitats while others face range restrictions.

Climate change will likely have a differential impact on different soft scale species, with some expanding their ranges and others experiencing contraction. Consistency with previous studies and expectations was observed (Shan et al. 2022; Wang et al. 2021; Ma et al. 2022; Azrag et al. 2022), indicating that climate warming is likely to expand the potential distribution ranges of many soft scale species. This expansion can be attributed to the direct effects of warming on insect development, survival, and reproduction (Frank 2021), leading to geographic range expansion and potential consequences for grapevine health. Similar trends have been observed in other insect pests such as *Heortia vitessoides* (Xu et al. 2020) and *Hyphantria cunea* (Ge et al. 2019). However, three species were identified in which climate change is predicted to cause a range contraction. This may be due to geographic barriers like coastlines, which force these species to move toward less suitable areas (Bellard et al. 2017; Pinsky et al. 2020).

A northward movement in the potential distribution of all seven soft scale species under future climate scenarios (SSP126 and SSP585) was revealed. This finding is similar to the observations in various taxa, including freshwater fishes (Chu et al. 2005), *Larix* trees (Mamet et al. 2018), birds (Hitch and Leberg 2007), and other insects (Zuliani et al. 2015), as a response to global warming. The likely explanation for this northward shift is temperature. An annual mean temperature between 9°C and 12°C (Bio1) was suggested as ideal for soft scale survival (e.g., PV). Climate warming may push temperatures at lower latitudes beyond this suitable range, potentially reducing development or fertility rates. Conversely, warming at higher latitudes may create suitable conditions where they previously did not exist.

### 4.3 Limitation and Future Directions

While this study offers the first global assessment of soft scale distribution under climate change, several aspects can be addressed in future research to improve model accuracy and comprehensiveness: (1) Incorporating biotic factors: The models are currently focused on environmental variables. Future iterations could benefit from including biotic interactions, such as species competition and predator–prey relationships (Gherghel et al. 2018), as well as the influence of geomorphology (Rudi et al. 2018). (2) Global grape distribution: Although the potential distribution of seven soft scale vectors of GLRaV‐3 was analyzed, a more complete picture of potential disease spread would be provided by considering the global distribution of grapevines. (3) Updated climate data: Integrating recent climate data from the past 20 years would enhance the models' relevance to contemporary conditions. Addressing these limitations will require additional model parameters, leading to more robust and reliable predictions for future studies.

### 4.4 Factors Limiting GLRaV‐3 Spread Beyond Vector Availability

The host plants of these seven insect vectors span at least 17 different families, with the grape genus being just one among them (García Morales et al. 2016). The host plants of *Ceroplastes rusci* cover 53 families and 96 genera, *Coccus longulus* 56 families and 142 genera, *Neopulvinaria innumerabilis* 29 families and 50 genera, *Parasaissetia nigra* 100 families and 294 genera, *Parthenolecanium corni* 54 families and 129 genera, *Parthenolecanium persicae* 37 families and 61 genera, and *Pulvinaria vitis* 17 families and 37 genera. Interestingly, GLRaV‐3 is not always detected in regions with competent vectors. In New Zealand, for example, *Pseudococcus longispinus* is widespread across viticultural regions, yet GLRaV‐3 prevalence is not uniform. Research suggests that cooler climates may limit vector activity and reduce virus transmission rates (Charles et al. 2009). Although these vectors are polyphagous and capable of thriving under diverse environmental conditions, the virus remains absent or occurs at low incidence in some of these same areas. This indicates that additional factors—such as climate, vector–virus–host interactions, and transmission dynamics—may constrain the virus's spread.

These patterns resemble observations in other pathosystems. For instance, *Xylella fastidiosa* , the causal agent of Pierce's disease, has a narrower climatic range than its sharpshooter vectors because of temperature constraints on both bacterial survival and vector activity (Purcell 1997).

### 4.5 Implications for GLRaV‐3 Management

Soft scale insects act as critical vectors for GLRaV‐3, a damaging disease affecting grapevines (Maree et al. 2013; Kruger and Douglas‐Smit 2013; Naidu et al. 2015). Effective control strategies for these insects are essential for maintaining grapevine health (Kruger and Douglas‐Smit 2013). The spread of soft scales occurs primarily through two mechanisms: human‐mediated movement (e.g., agricultural trade) and wind dispersal (Gullan and Kosztarab 1997). Understanding these mechanisms is vital for developing strategies to prevent the spread of both soft scales and GLRaV‐3.

The importance of agricultural trade regulations and plant movement restrictions in minimizing the spread of soft scales was emphasized (Paini et al. 2016; Bebber et al. 2014). This is particularly relevant for species like PV (unsuitable in Australia and South America), PP (unsuitable in South America and South Africa), PC (unsuitable in Australia and South Africa), and CL (unsuitable in vast areas including Australia, Africa, and South America). In regions where these diseases are established, targeted quarantine measures, field controls, and eradication efforts to prevent further outbreaks may be guided by the findings.

## 5 Conclusions

Grapevine leafroll disease (GLRaV‐3), transmitted by soft scale insects, poses a significant threat to vineyards worldwide. This study employed machine learning techniques, specifically the MaxEnt model, to predict the future global distribution of seven soft scale vector species under various climate change scenarios. The current distribution of these vectors was found to be primarily constrained by temperature, which accounted for 46.4%–78.6% of suitable habitat areas. Climate change is projected to substantially modify these distribution patterns, with most soft scale species experiencing up to a 60% increase in suitable habitats and a northward shift in their distribution centers. These changes suggest a heightened risk of GLRaV‐3 spread in new regions, emphasizing the need for proactive management strategies. By identifying potential future hotspots for soft scale populations, this research provides critical insights for the development of targeted surveillance and control measures, thereby aiding in the protection of global grape production against these detrimental pests.

## Author Contributions

**Minmin Niu:** data curation (equal), formal analysis (equal), methodology (equal), software (equal), writing – original draft (equal), writing – review and editing (equal). **Yunyun Lu:** methodology (equal), software (equal), validation (equal), writing – original draft (equal). **Boxiang Zhao:** software (equal). **Fengxia Dong:** data curation (equal). **Junfei Bi:** formal analysis (equal). **Pengfei Jing:** project administration (equal). **Kangjie Wang:** project administration (equal). **Zhengyuan Liu:** project administration (equal). **Jiufeng Wei:** conceptualization (equal), data curation (equal), formal analysis (equal), methodology (equal), supervision (equal), writing – original draft (equal), writing – review and editing (equal). **Wei Ji:** conceptualization (equal), funding acquisition (equal), project administration (equal), writing – review and editing (equal).

## Conflicts of Interest

The authors declare no conflicts of interest.

## Supporting information


**Appendix S1:** ece372297‐sup‐0001‐AppendixS1.zip.

## Data Availability

The data that support the findings of this study are openly available in the Appendix S1.
