# Supplementary material for: Climate Change Drives the Distribution of Insect Vectors for GLRaV‐3 on a Global Scale
Source: Ecol Evol. 2025 Oct 14;15(10):e72297. doi: 10.1002/ece3.72297 (PMC12521802; doi:10.1002/ece3.72297)

**Fig. S1.** ENMeval results for soft scales from SDMs

**(1) *Ceroplastes rusci* (CR)**


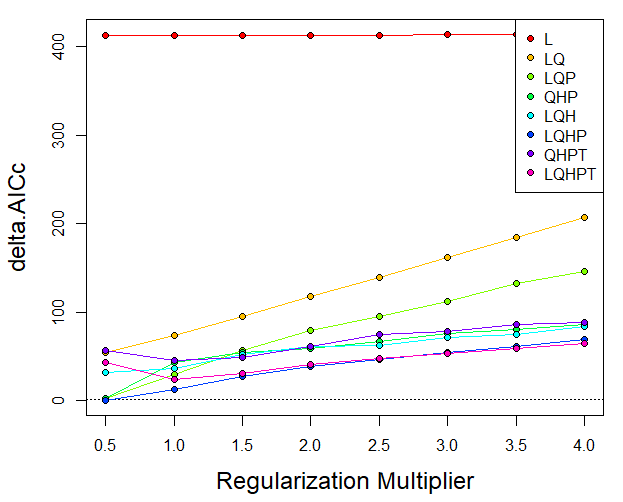


**(2) *Coccus longulus* (CL)**


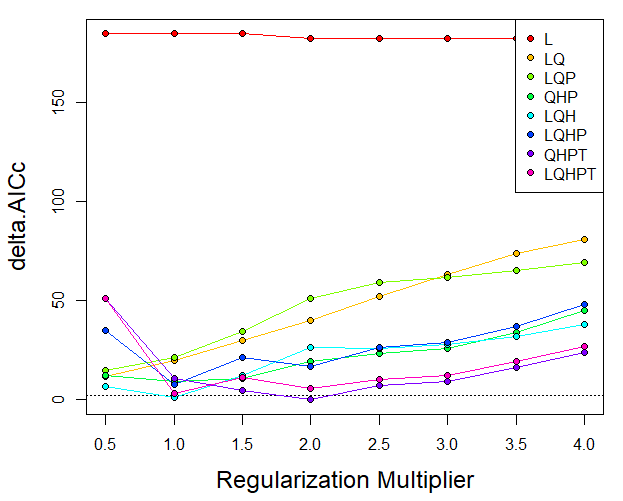


**(3) *Neopulvinaria innumerabilis* (NI)**


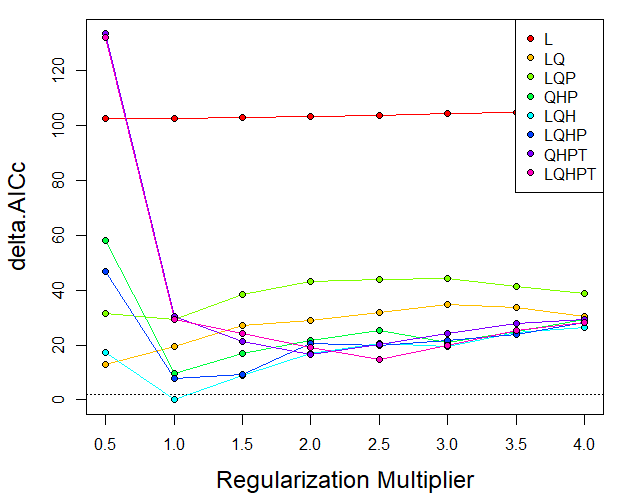


**(4) *Parasaissetia nigra* (PN)**


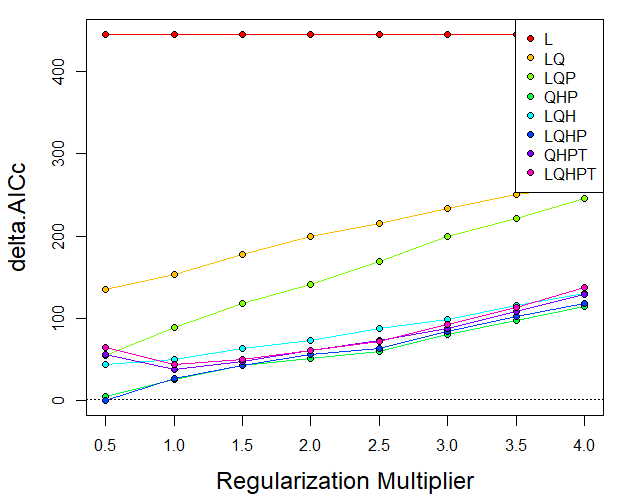


**(5) *Parthenolecanium corni* (PC)**


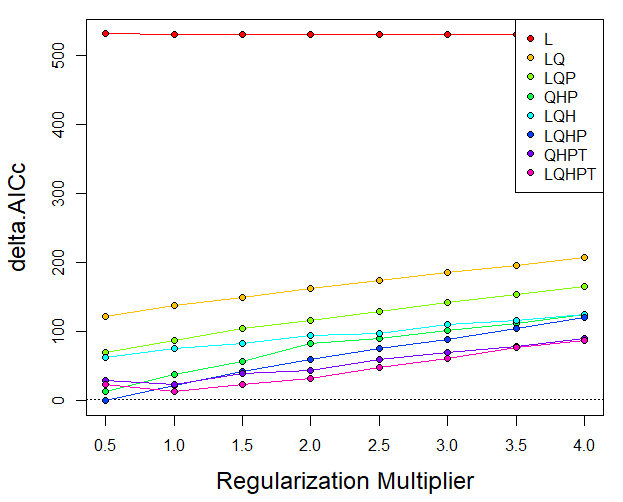


**(6)** ***Parthenolecanium persicae* (PP)**


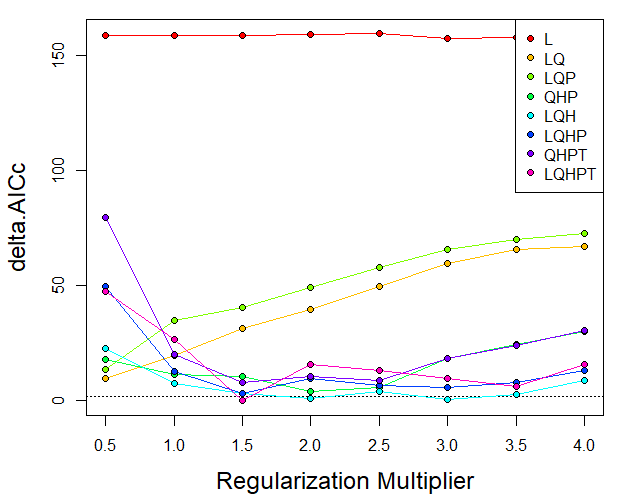


**(7) *Pulvinaria vitis* (PV)**


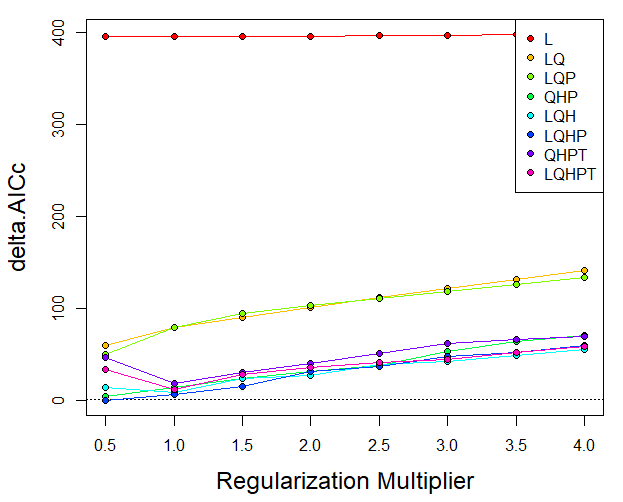

Supplement: Supplementary file 1 — Appendix S1: ece372297‐sup‐0001‐AppendixS1.zip. [file ECE3-15-e72297-s001.zip › ece372297-sup-0001-FigureS1.docx]
