# Supplementary material for: Climate Change Drives the Distribution of Insect Vectors for GLRaV‐3 on a Global Scale
Source: Ecol Evol. 2025 Oct 14;15(10):e72297. doi: 10.1002/ece3.72297 (PMC12521802; doi:10.1002/ece3.72297)
Supplement: Supplementary file 1 — Appendix S1: ece372297‐sup‐0001‐AppendixS1.zip. [file ECE3-15-e72297-s001.zip › ece372297-sup-0003-FigureS3.docx]

**Fig. S3.** Expansion, constraction and stable for soft scale insects under climate change (yellow represent constraction, red represent expansion, green represent stable; Enlarged versions of the individual images depicted in the figure are provided; A: 126-30; B: 126-50; C: 126-70; D: 126-90; E: 585-30; F: 585-50; G: 585-70; H: 585-90).

**(1) *Ceroplastes rusci* (CR)**


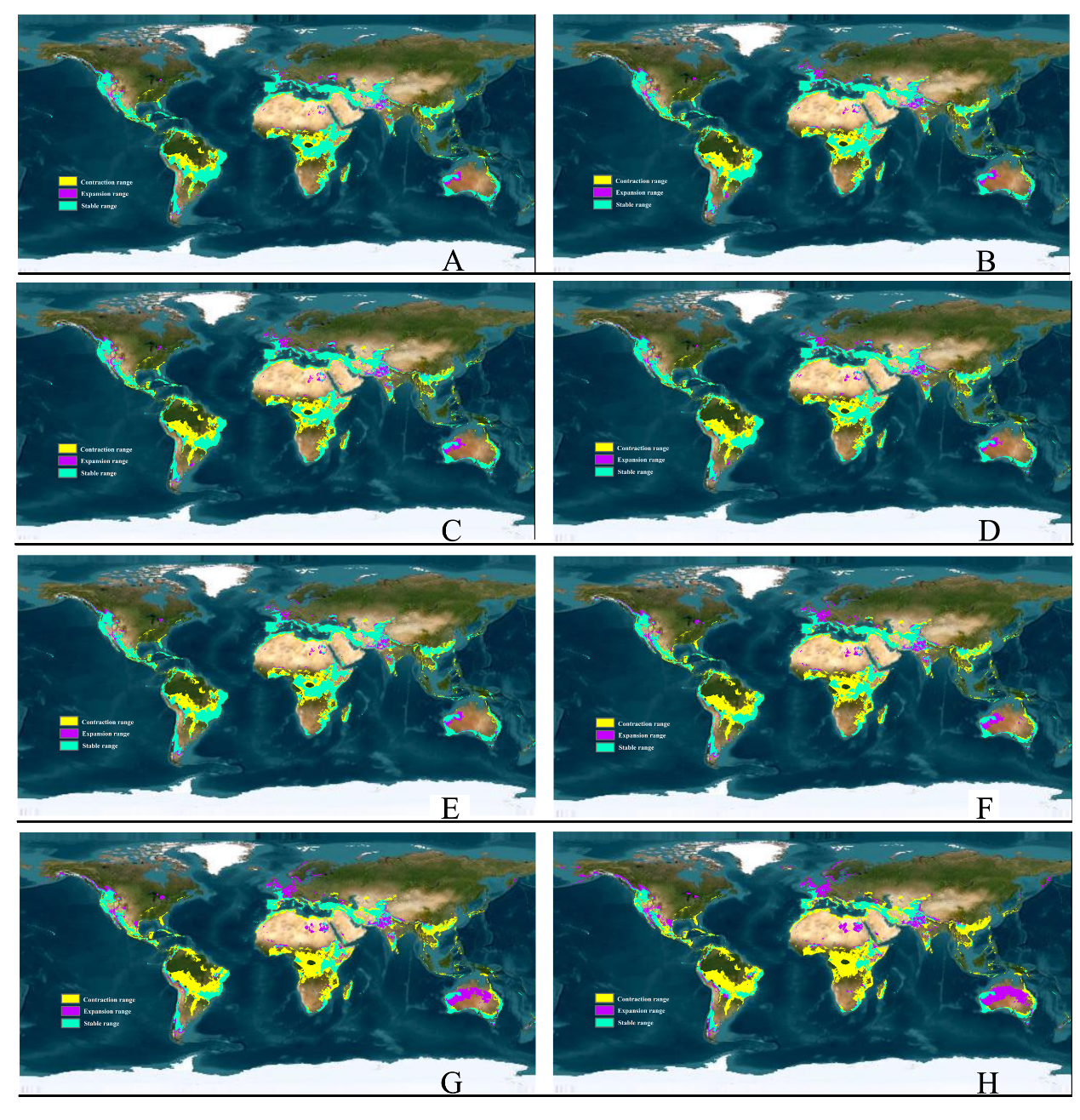


**A**


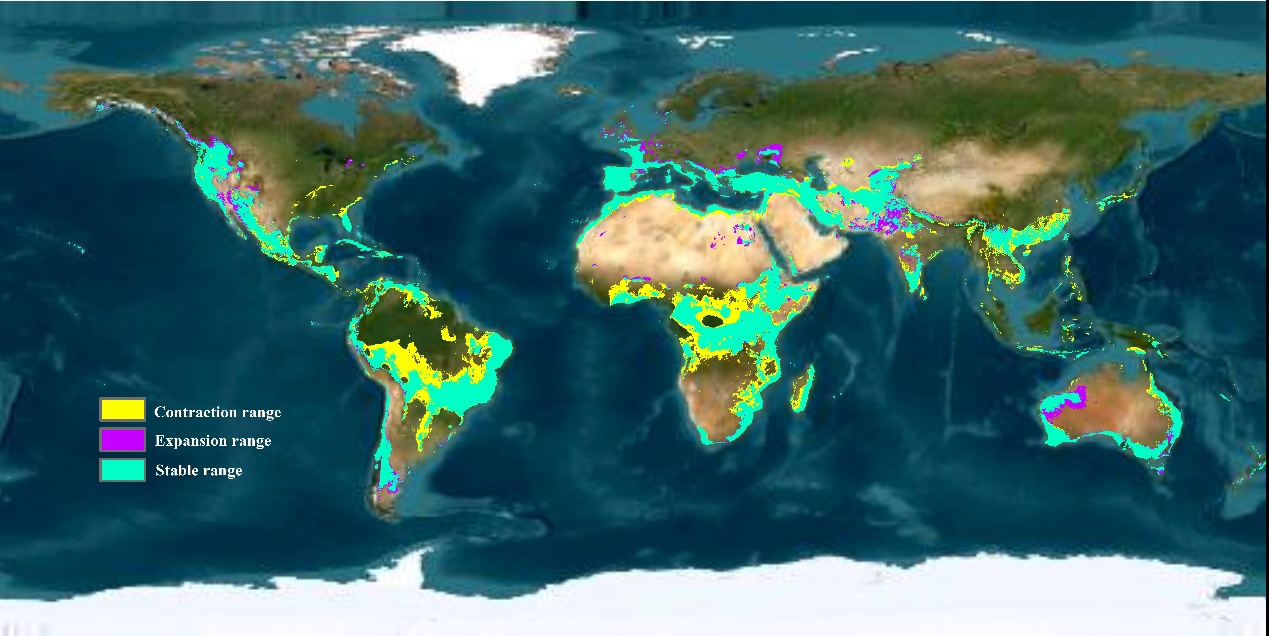


**B**


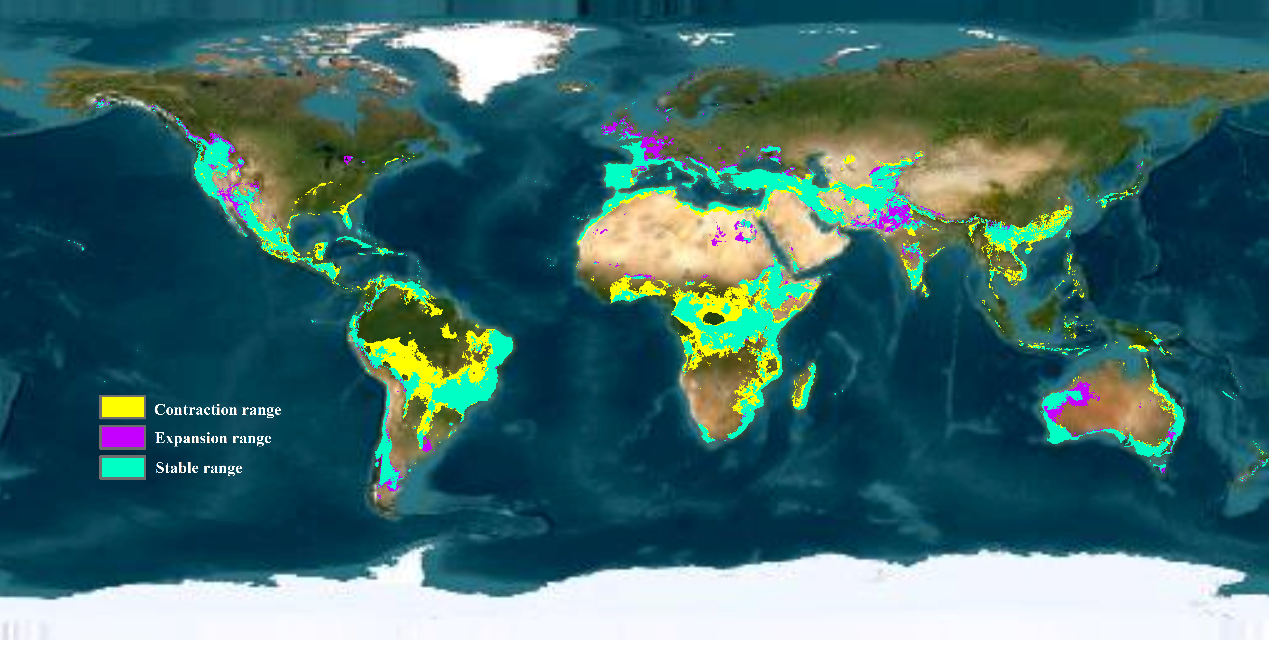


**C**


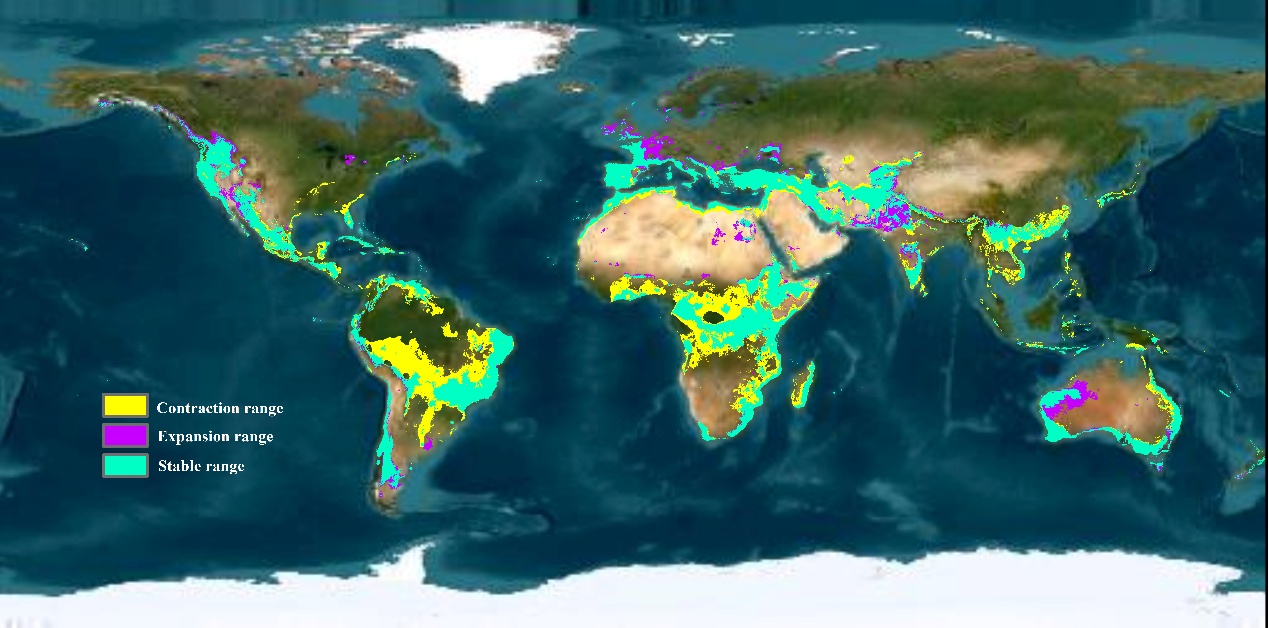


**D**


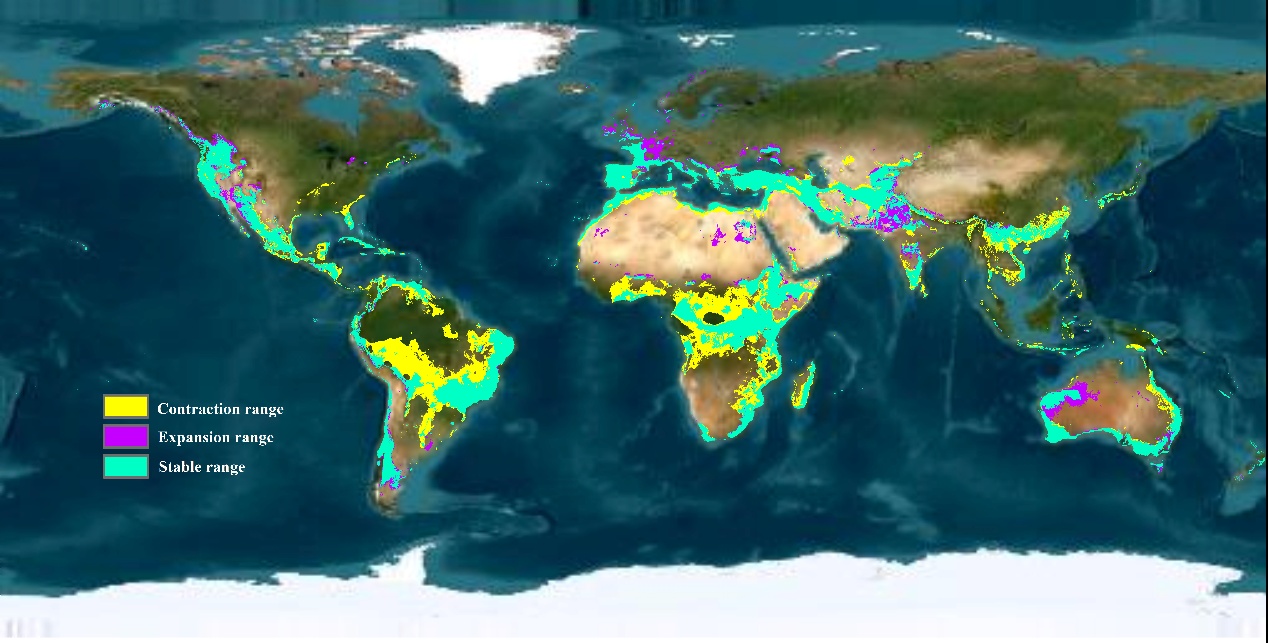


**E**


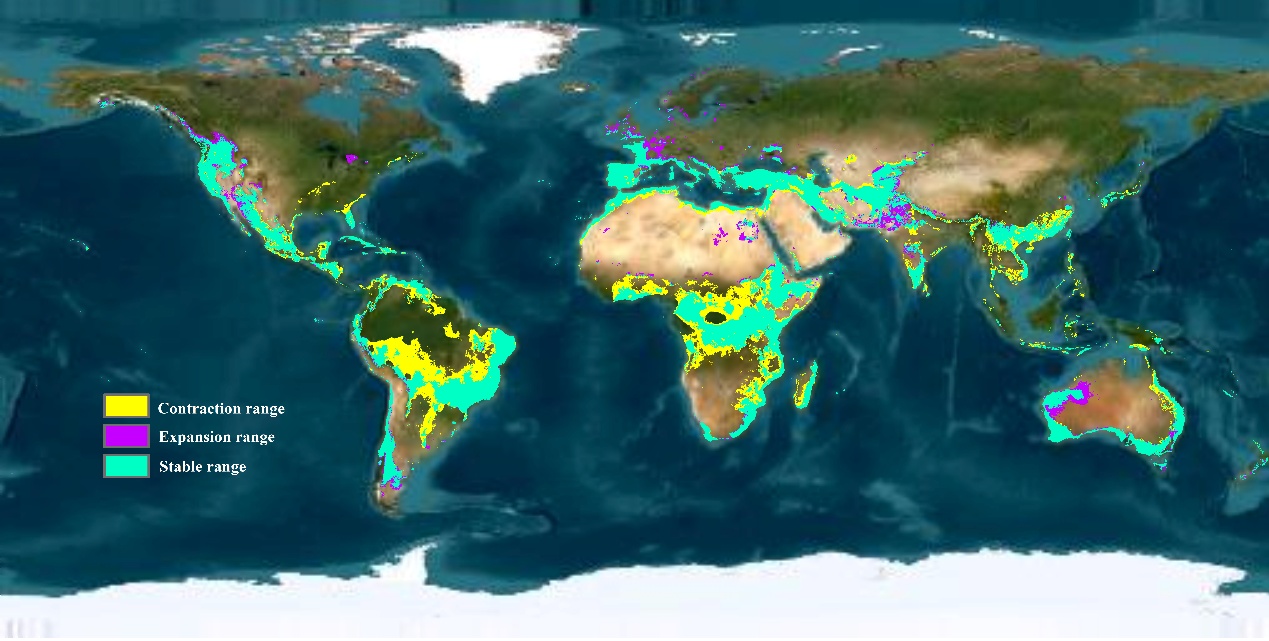


**F**


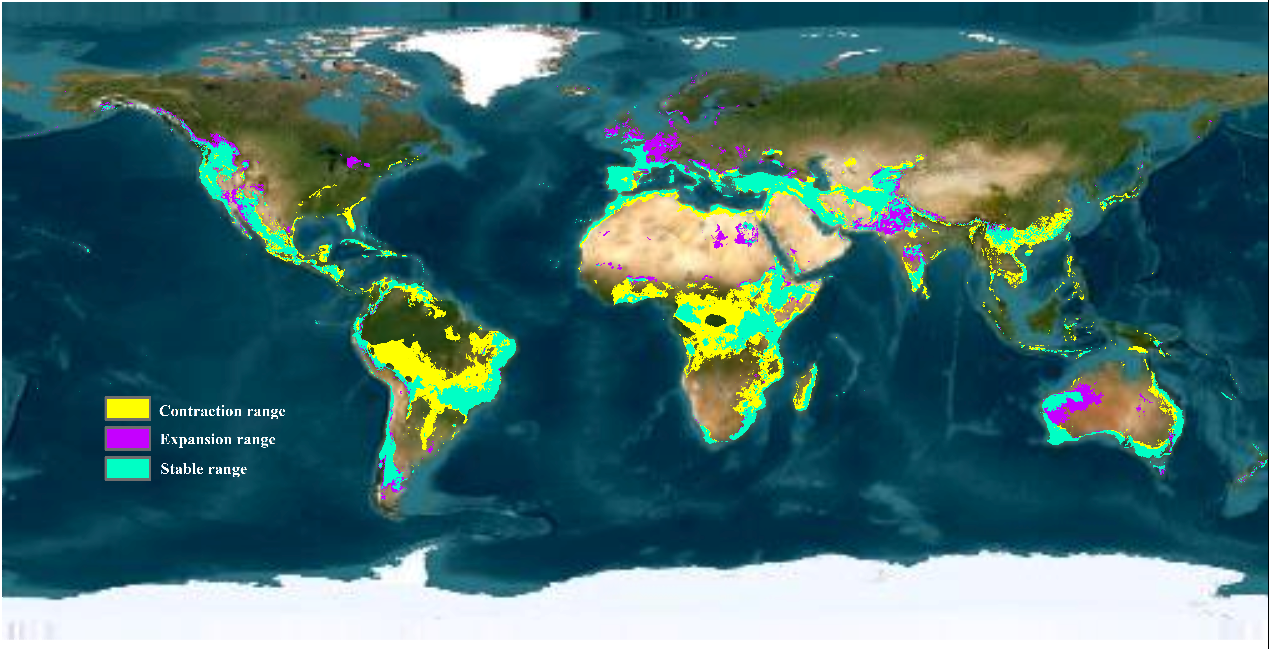


**G**


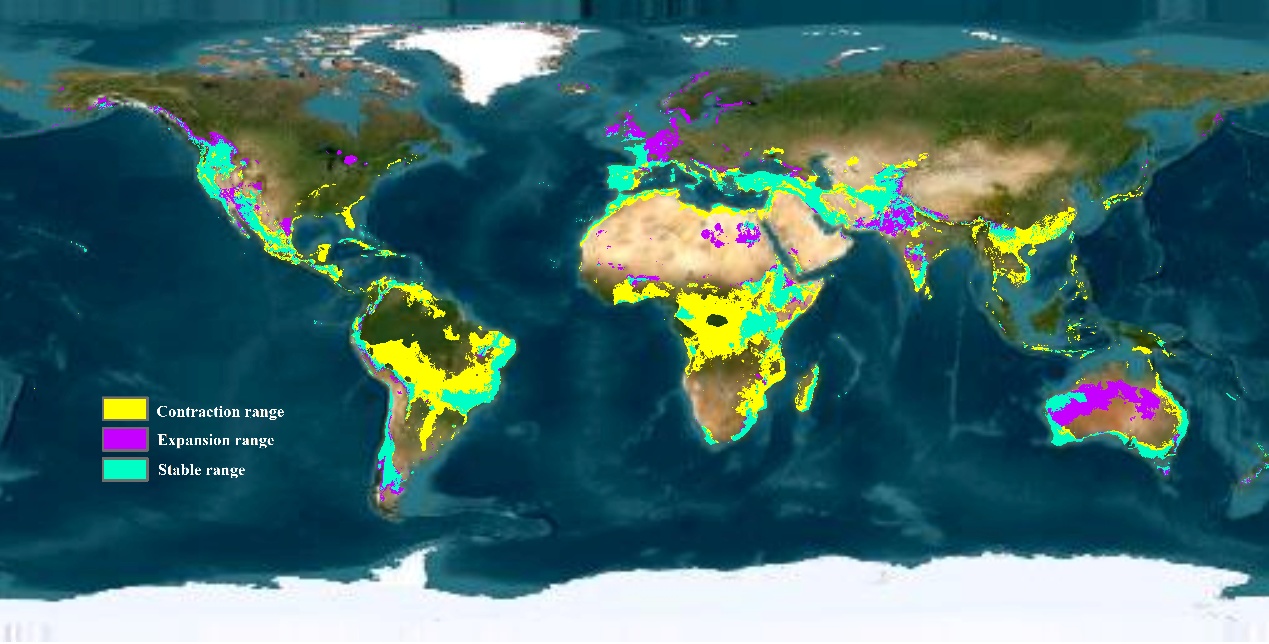


**H**


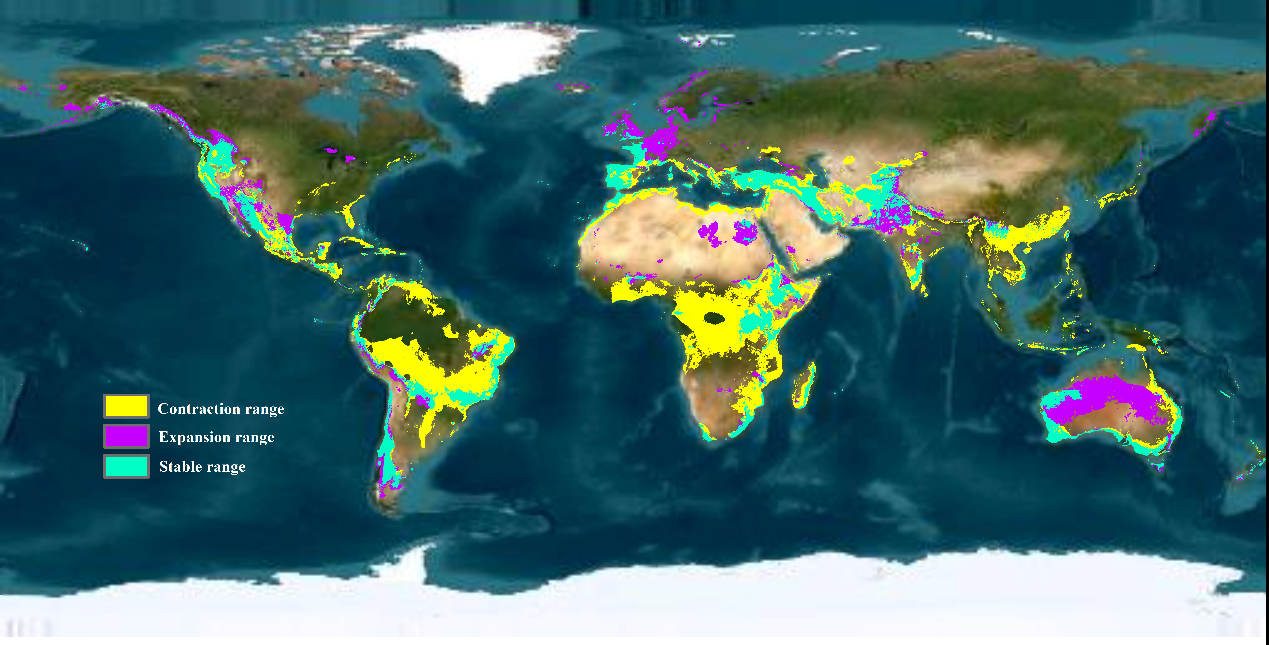


**(2) *Coccus longulus* (CL)**


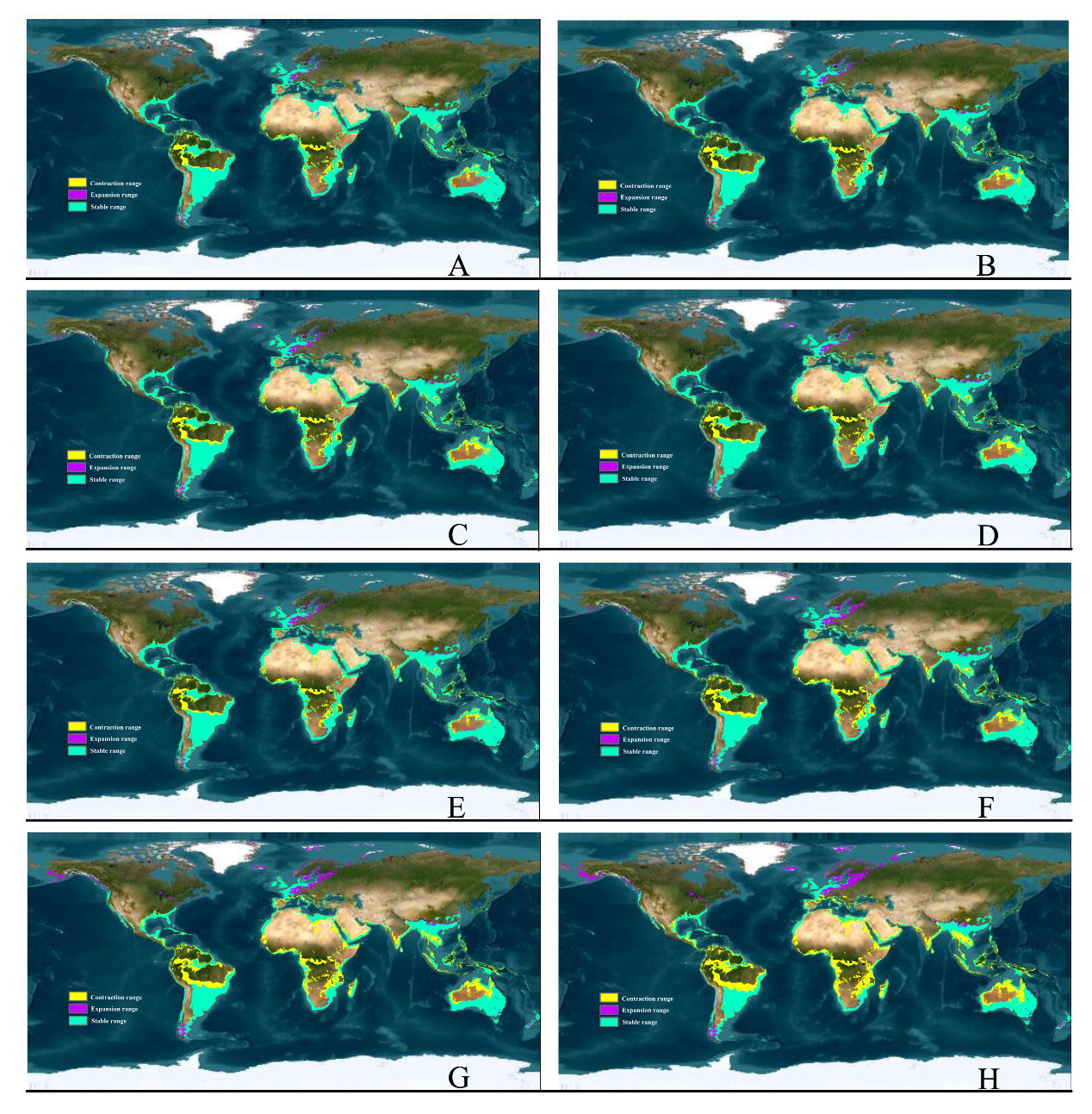


**A**

**
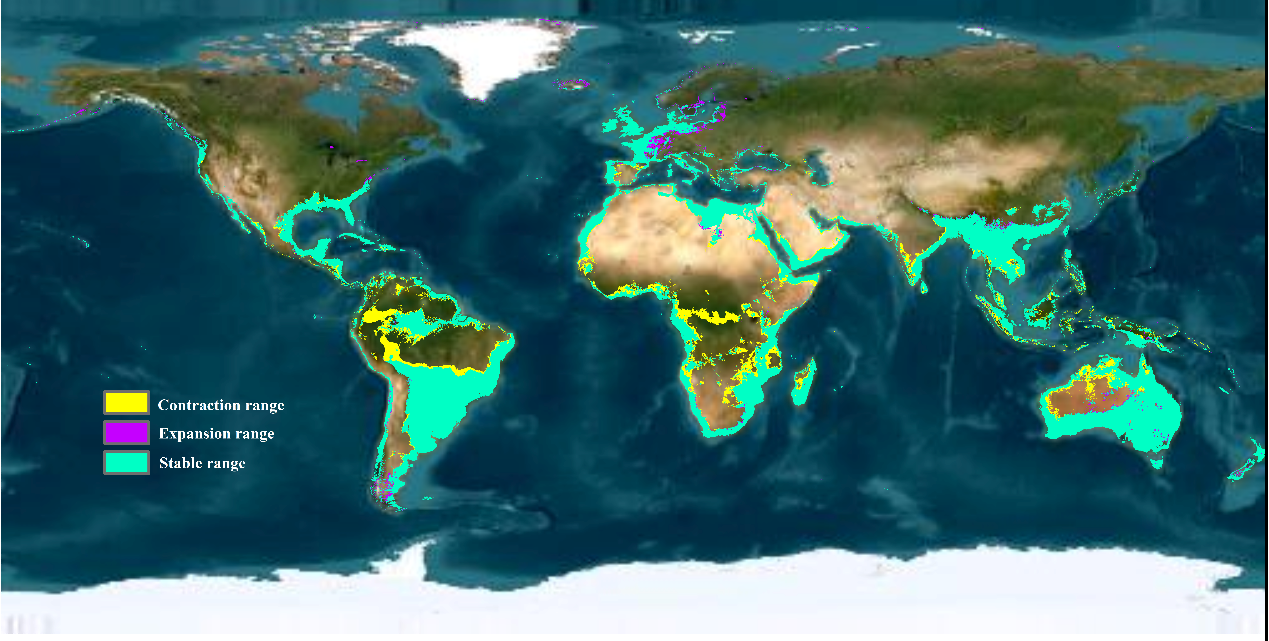
**

**B**

**
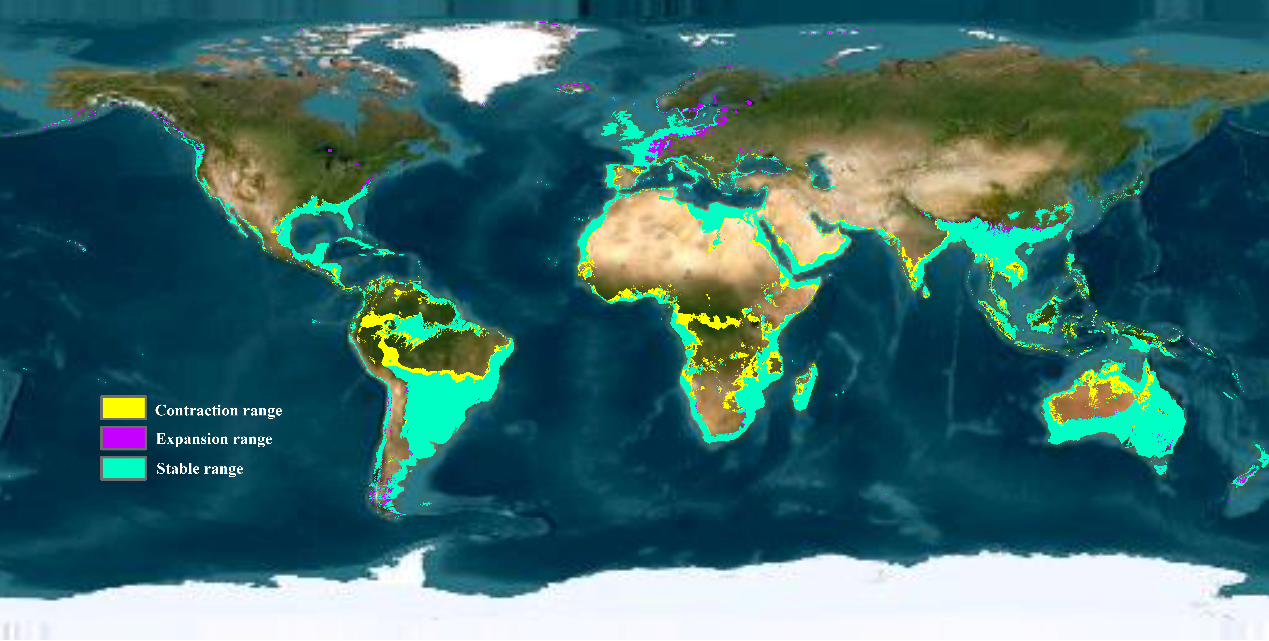
**

**C**

**
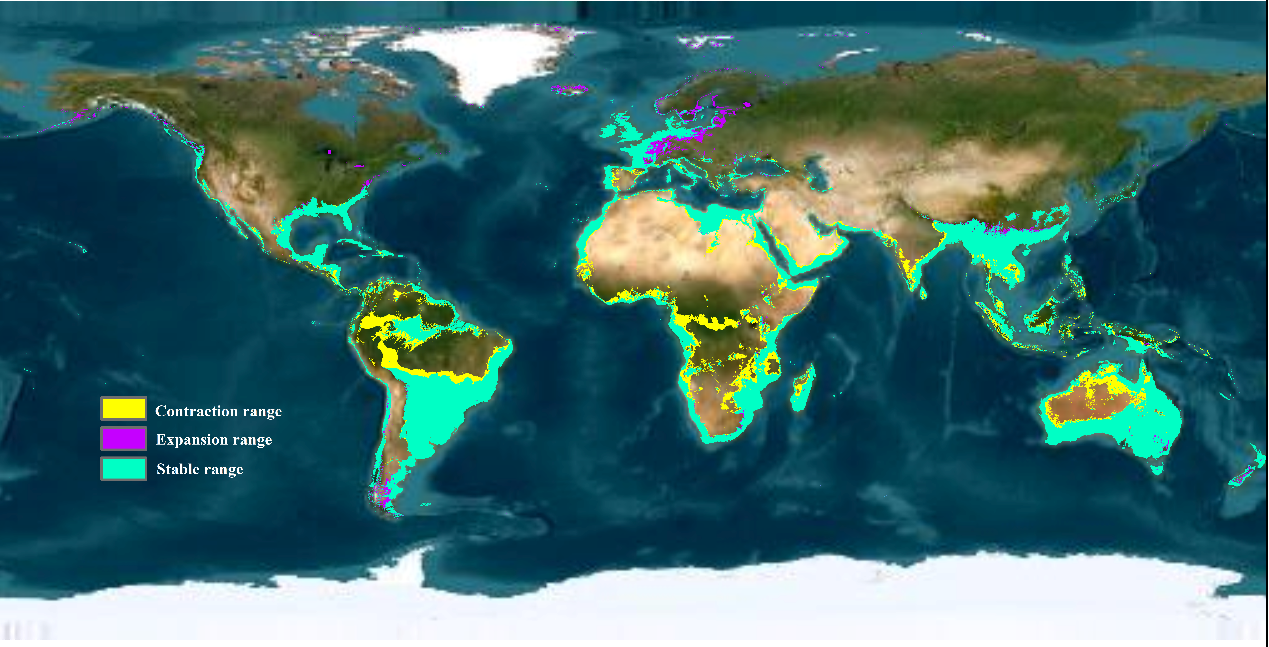
**

**D**

**
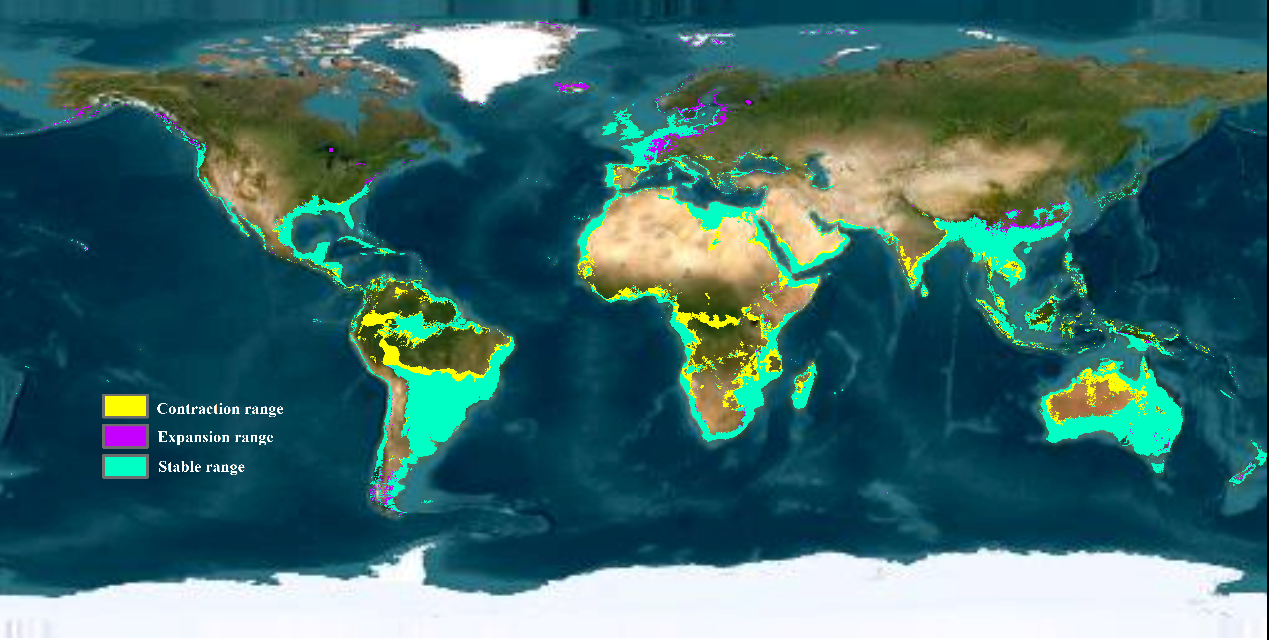
**

**E**

**
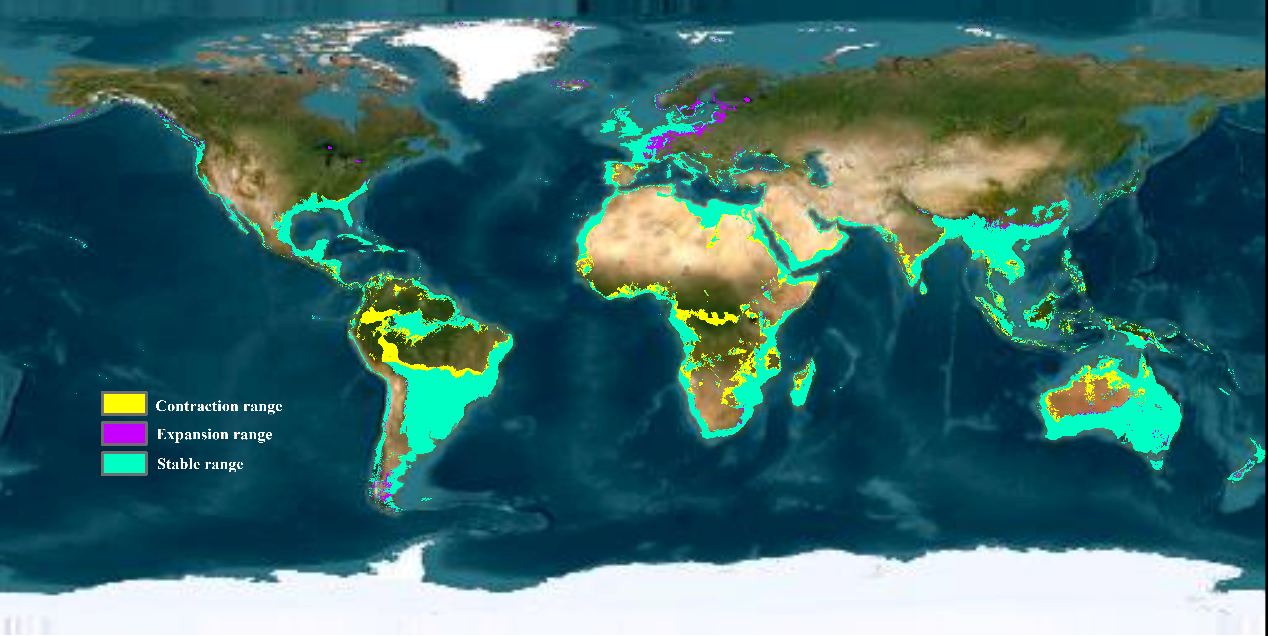
**

**F**

**
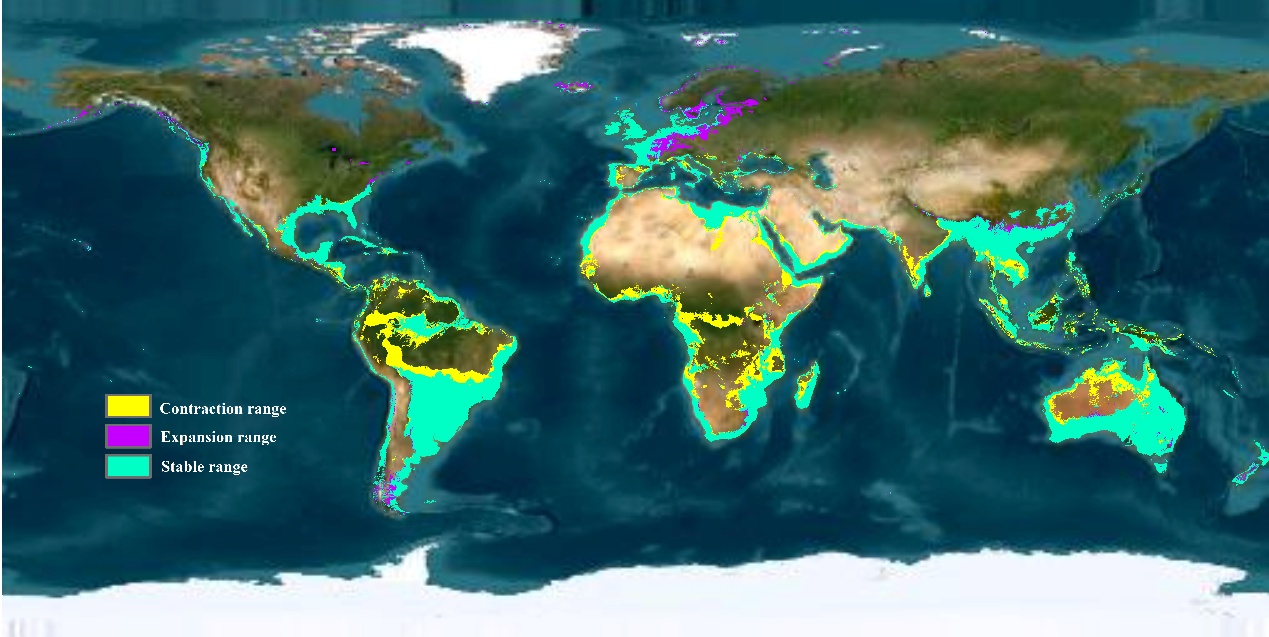
**

**G**

**
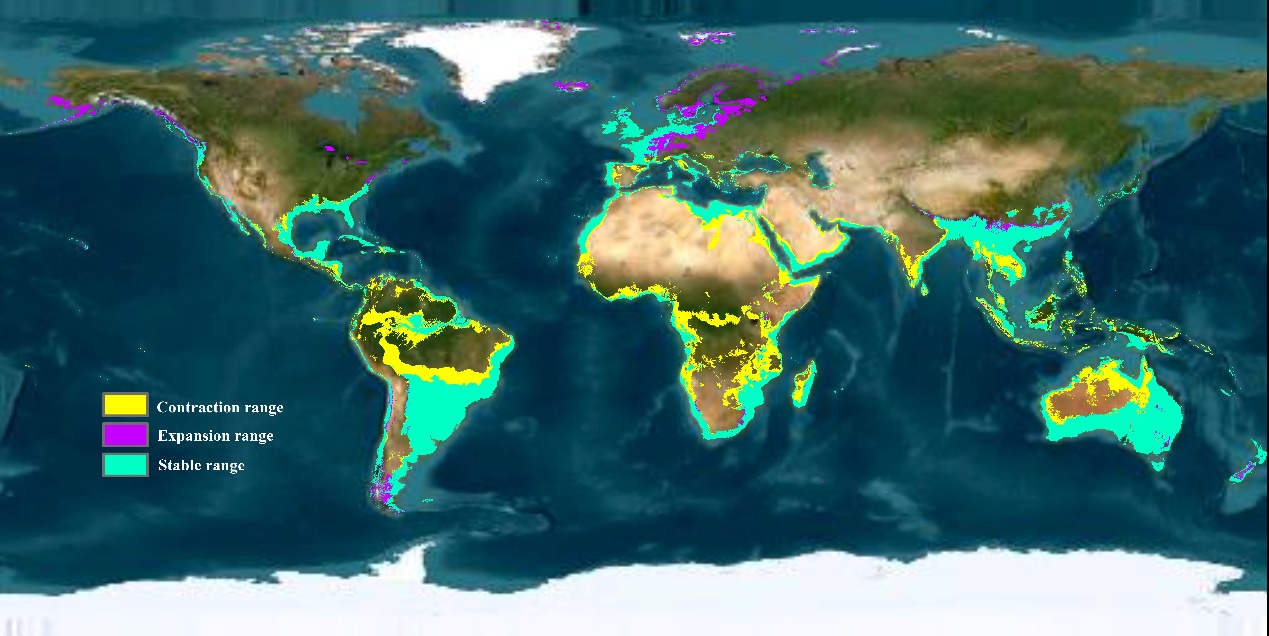
**

**H**

**
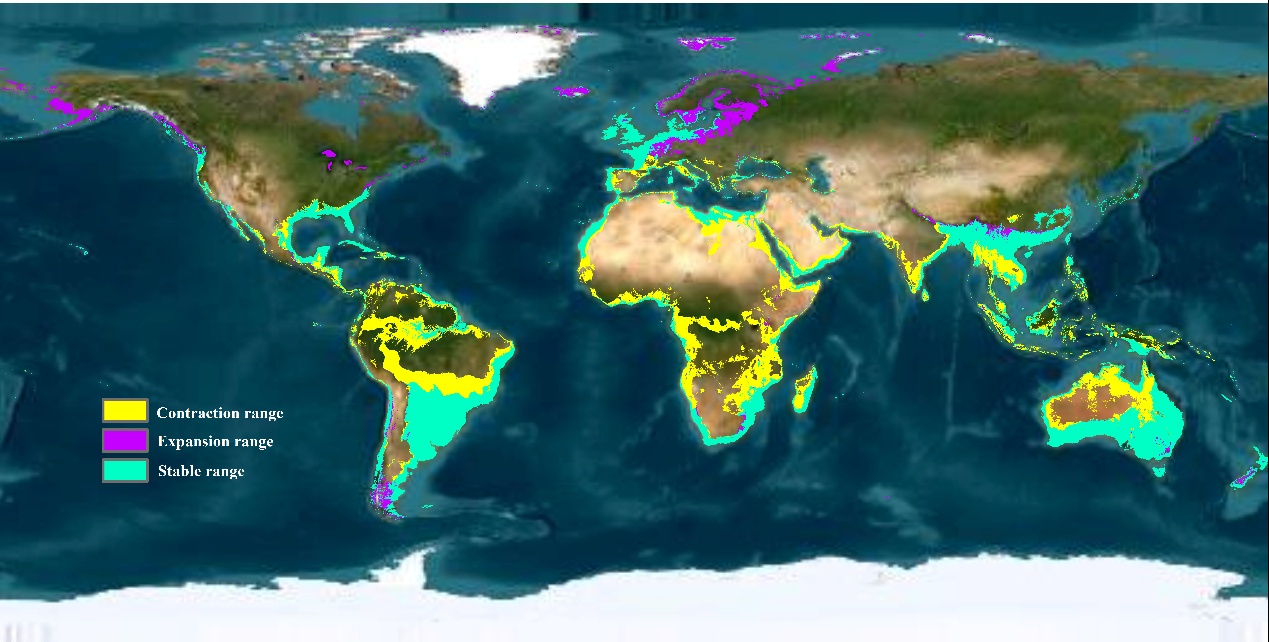
**

**(3) *Neopulvinaria innumerabilis* (NI)**


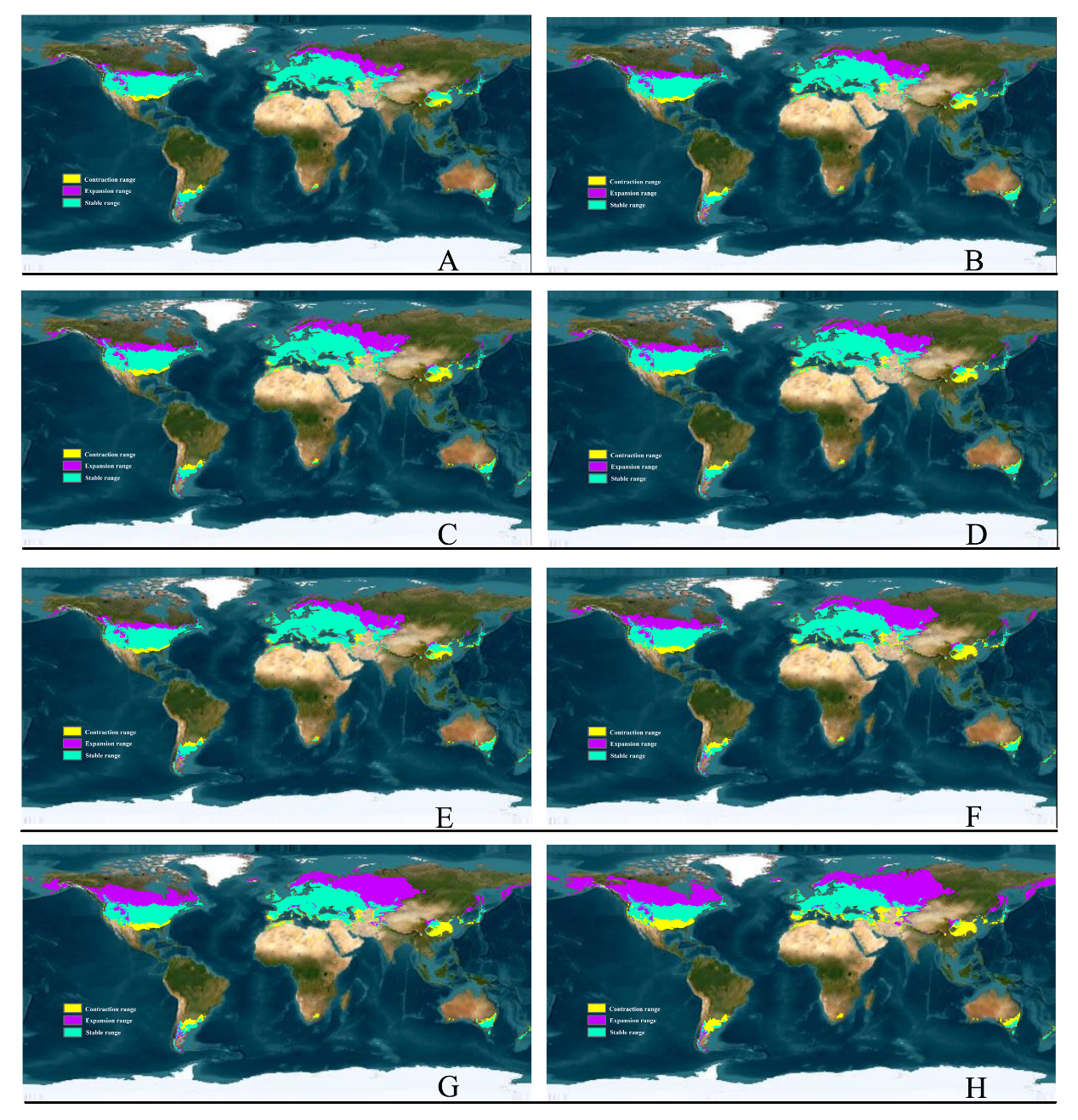


**A**

**
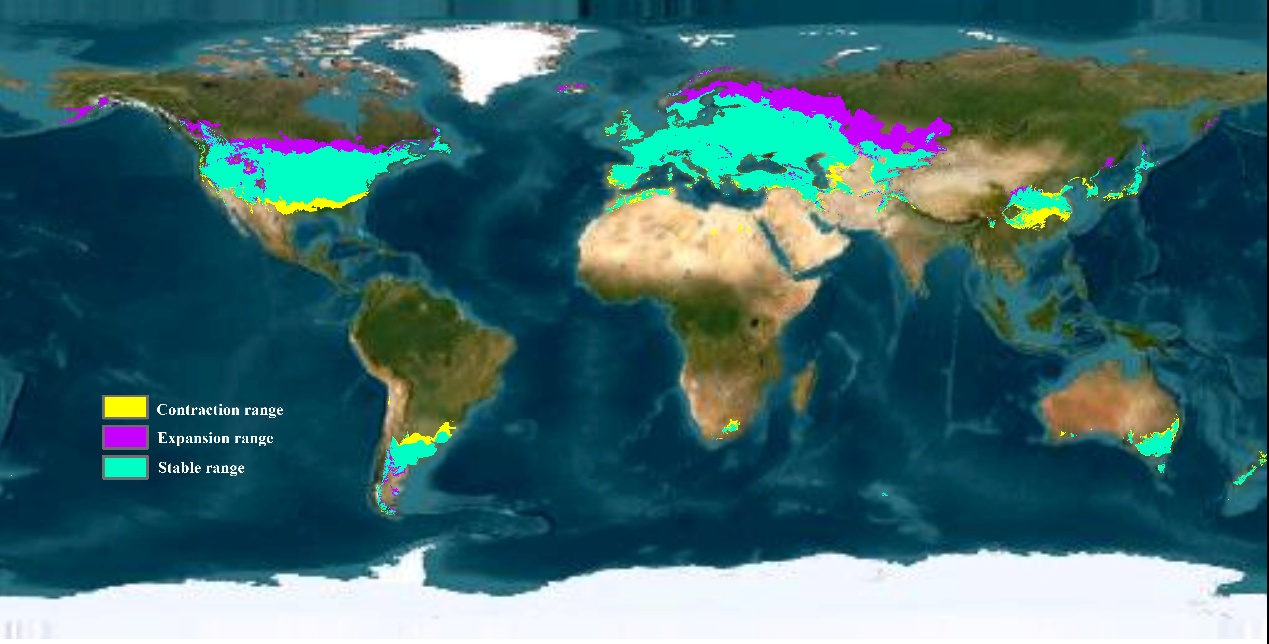
**

**B**

**
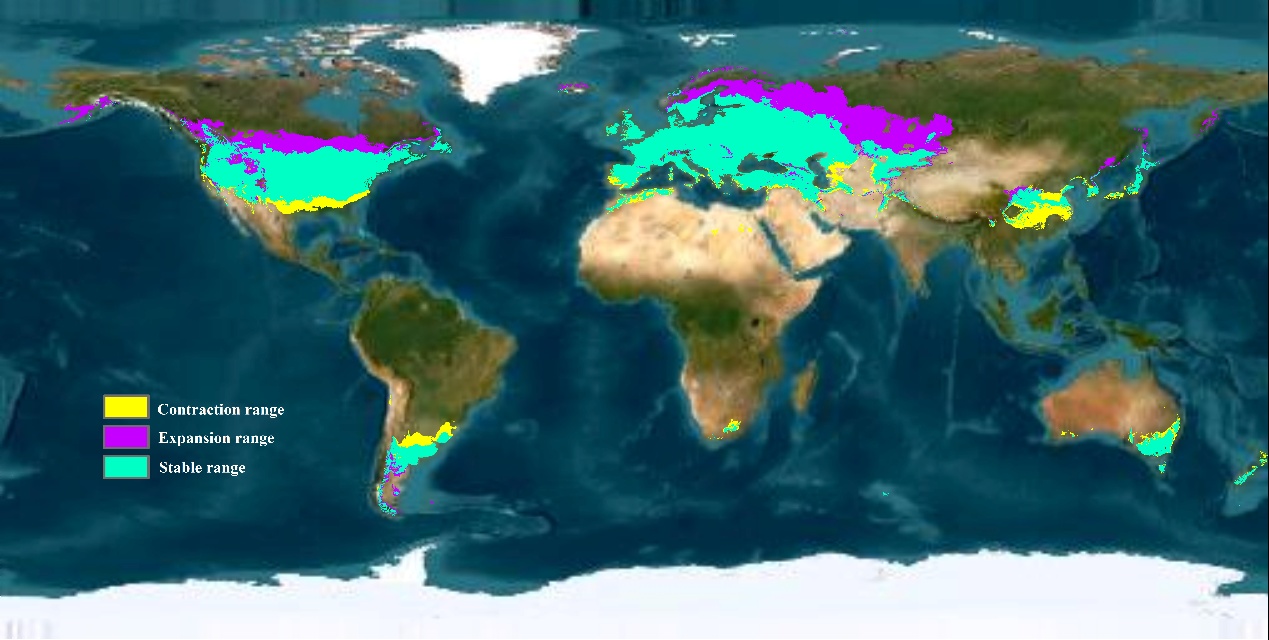
**

**C**

**
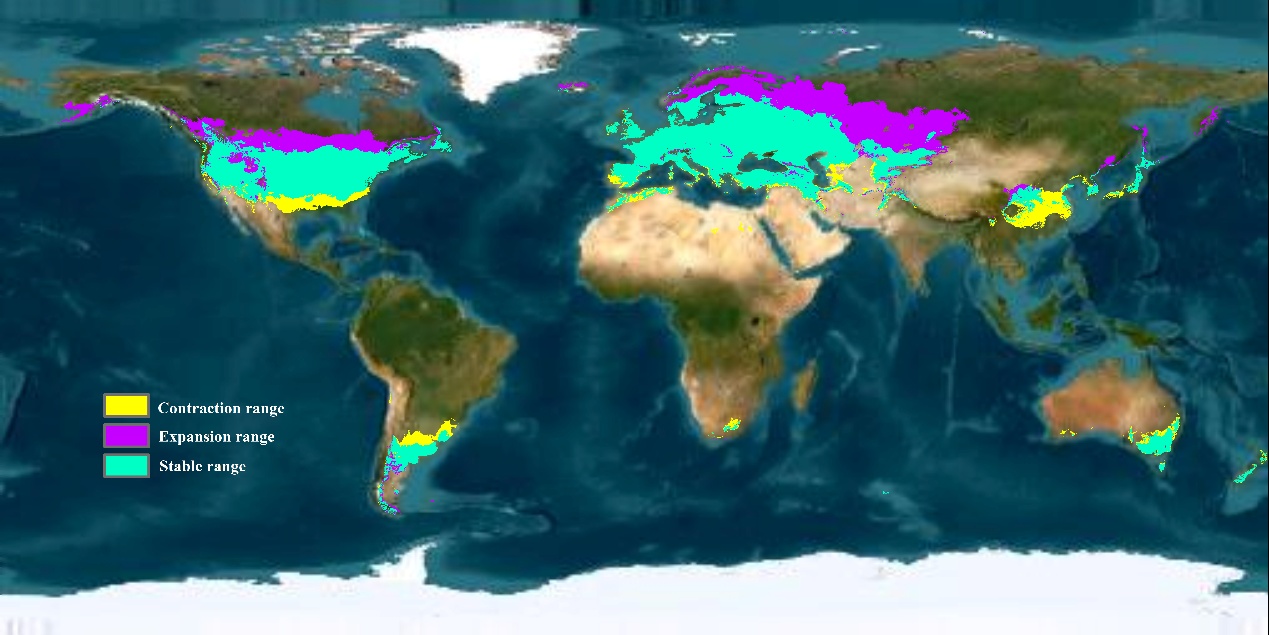
**

**D**

**
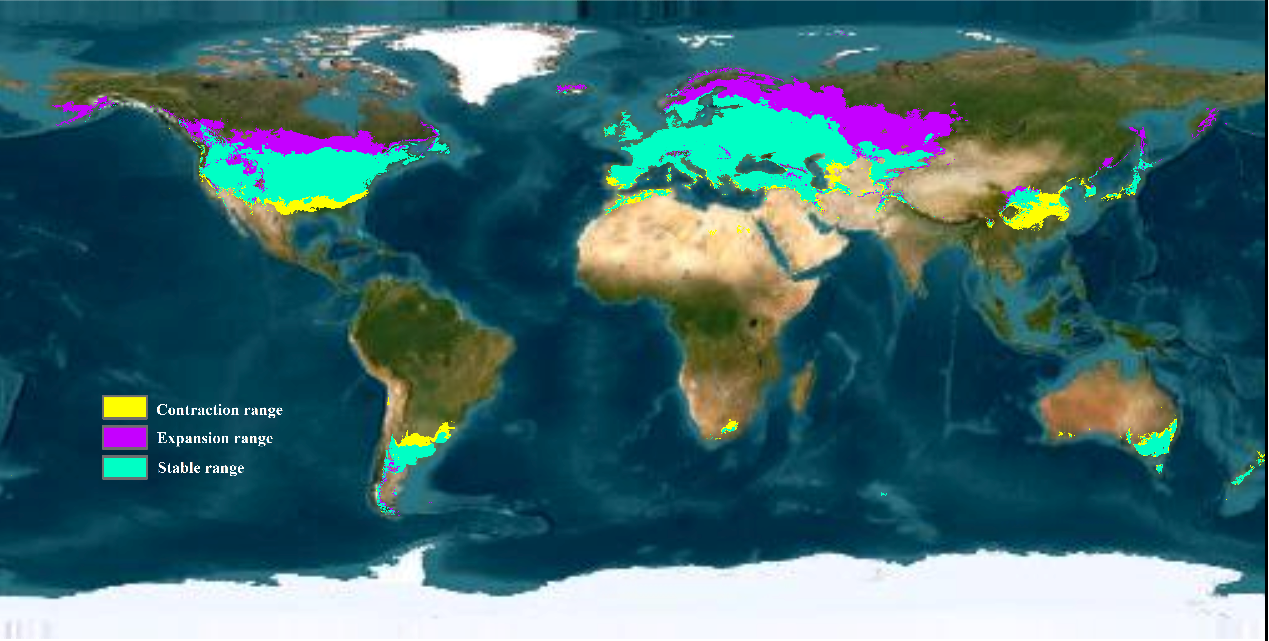
**

**E**

**
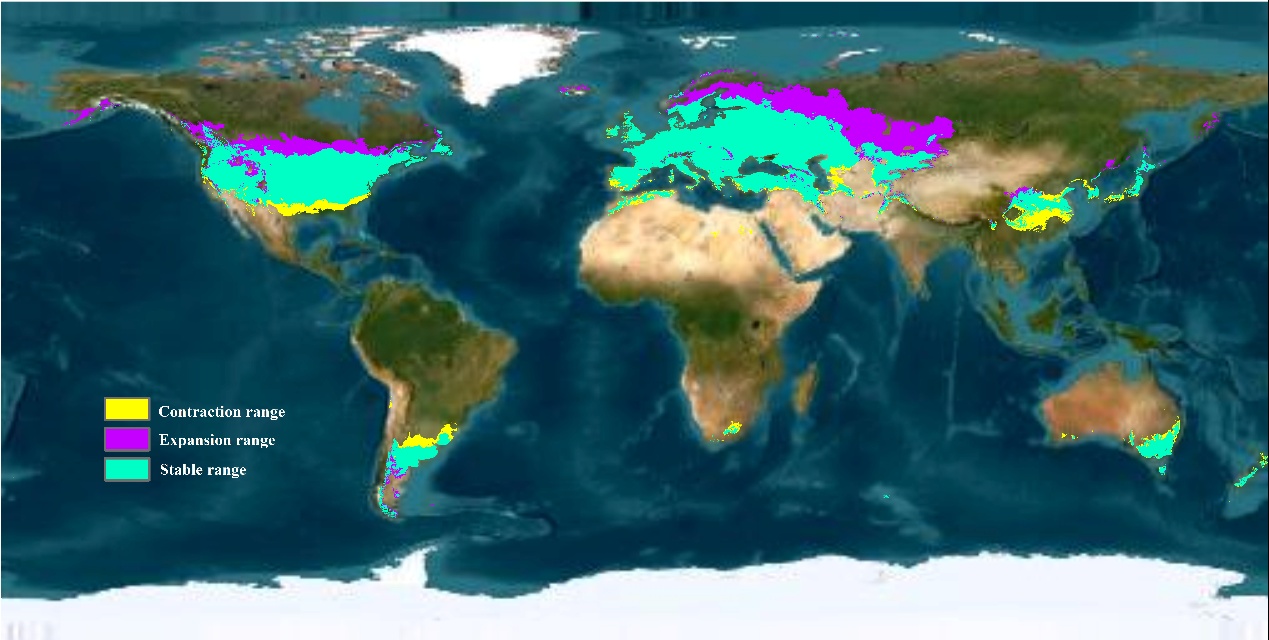
**

**F**

**
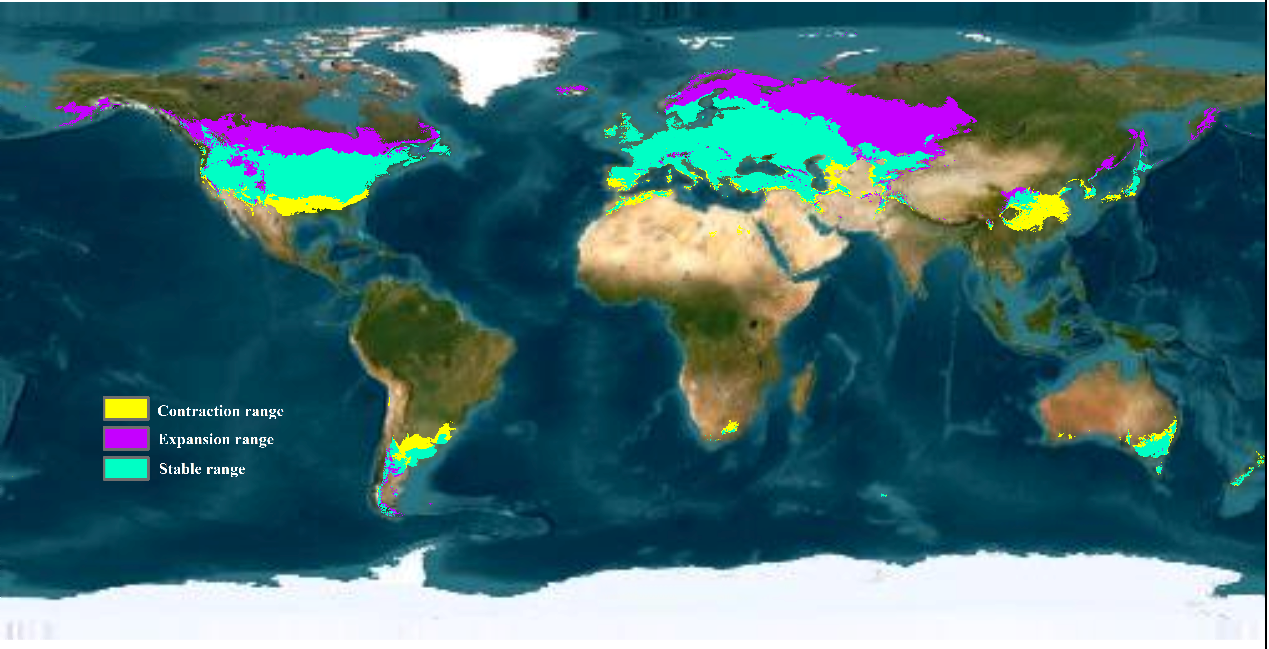
**

**G**

**
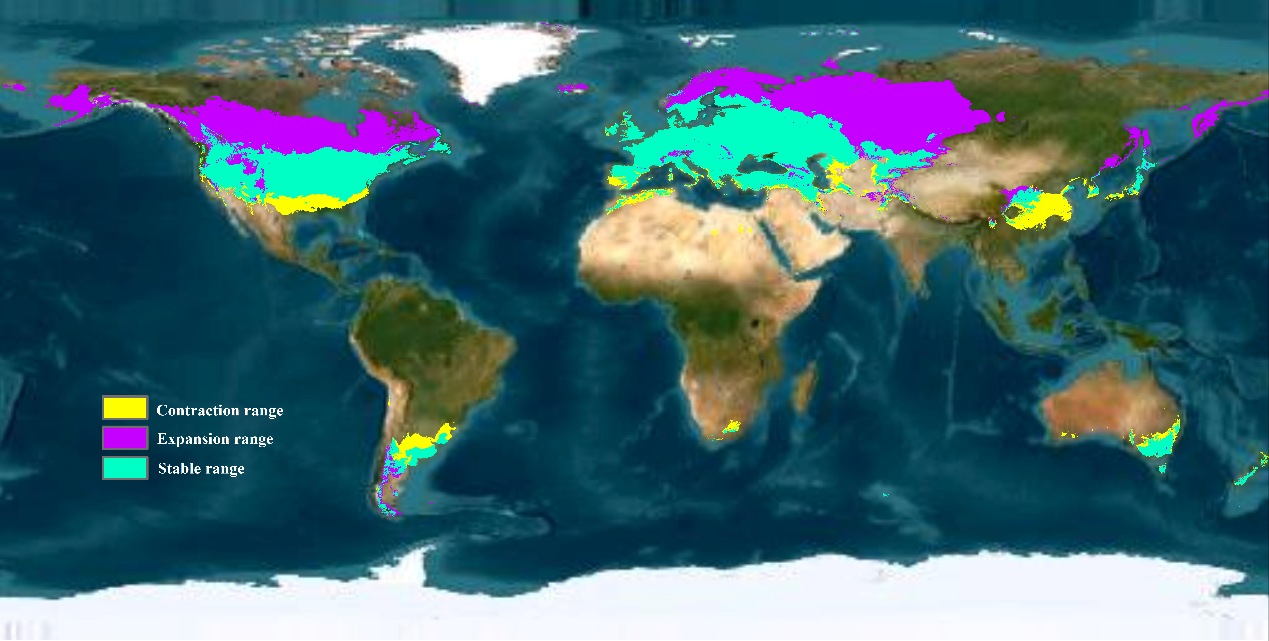
**

**H**

**
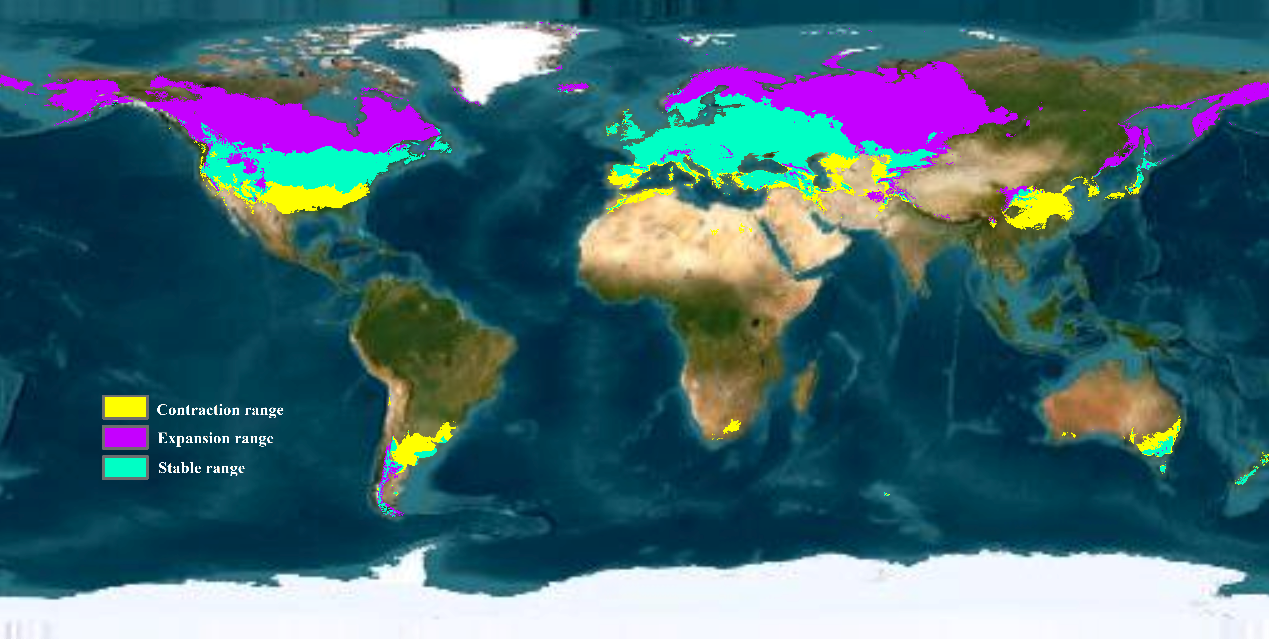
**

**(4) *Parasaissetia nigra* (PN)**


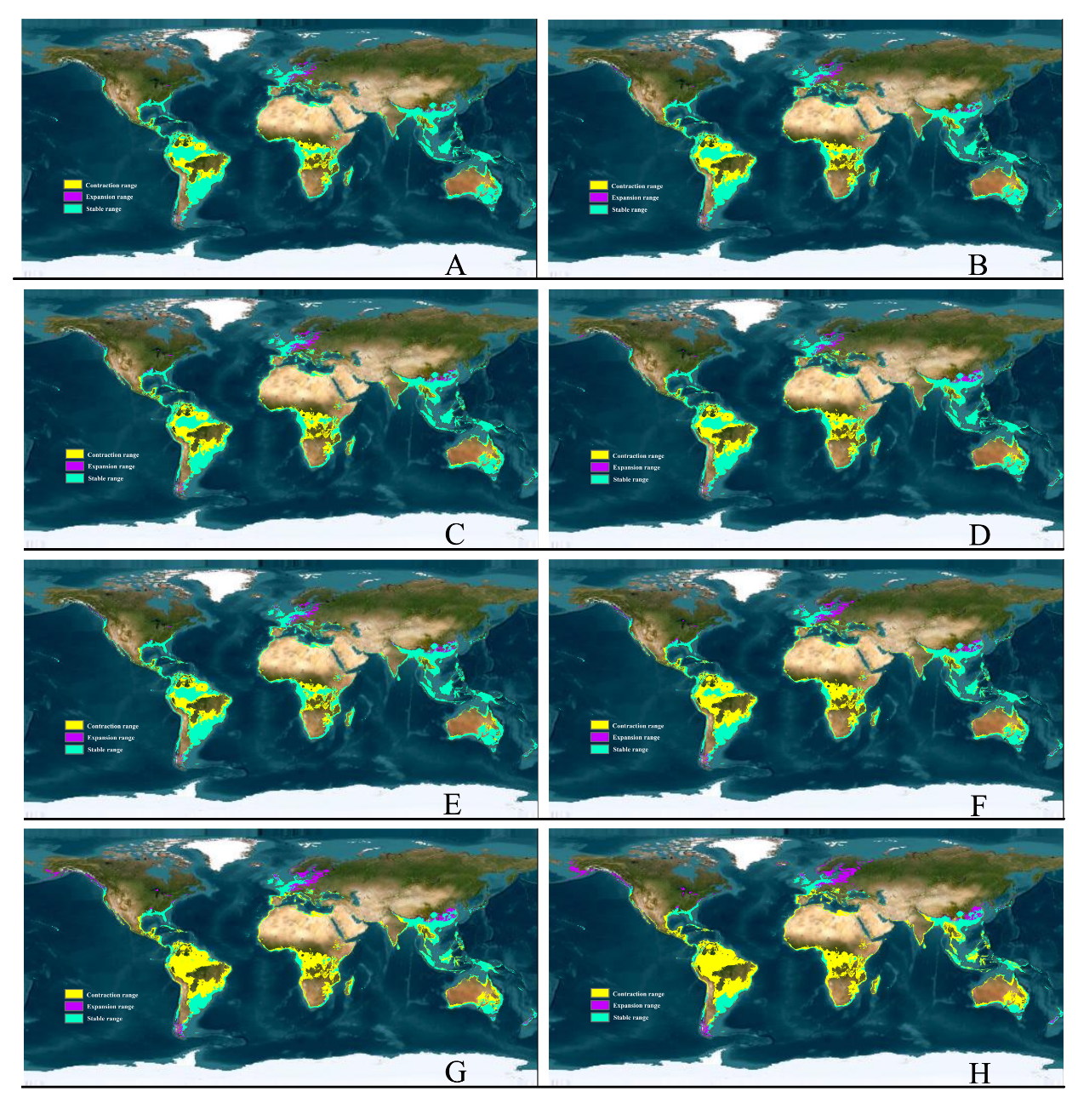


**A**

**
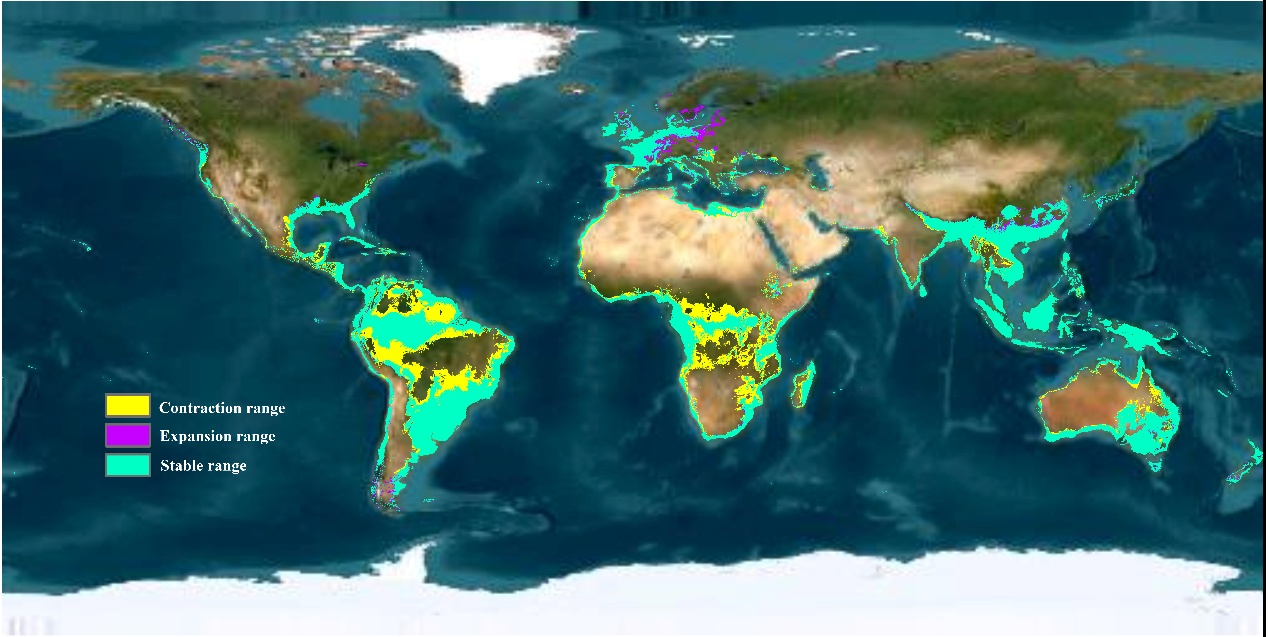
**

**B**

**
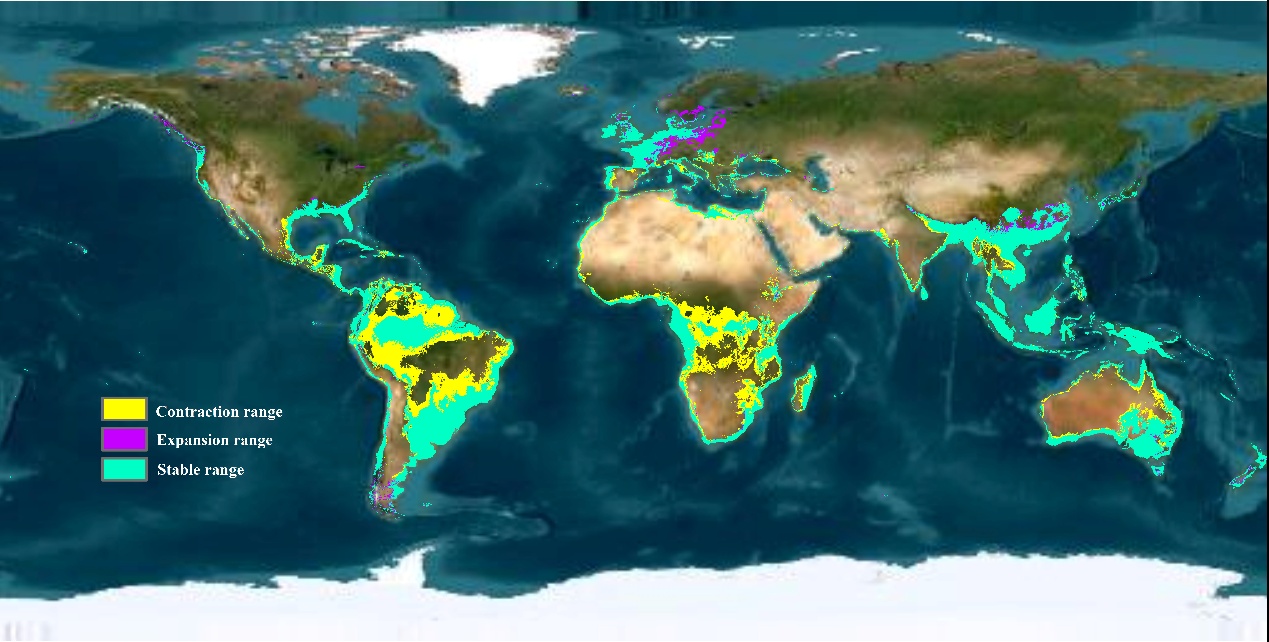
**

**C**

**
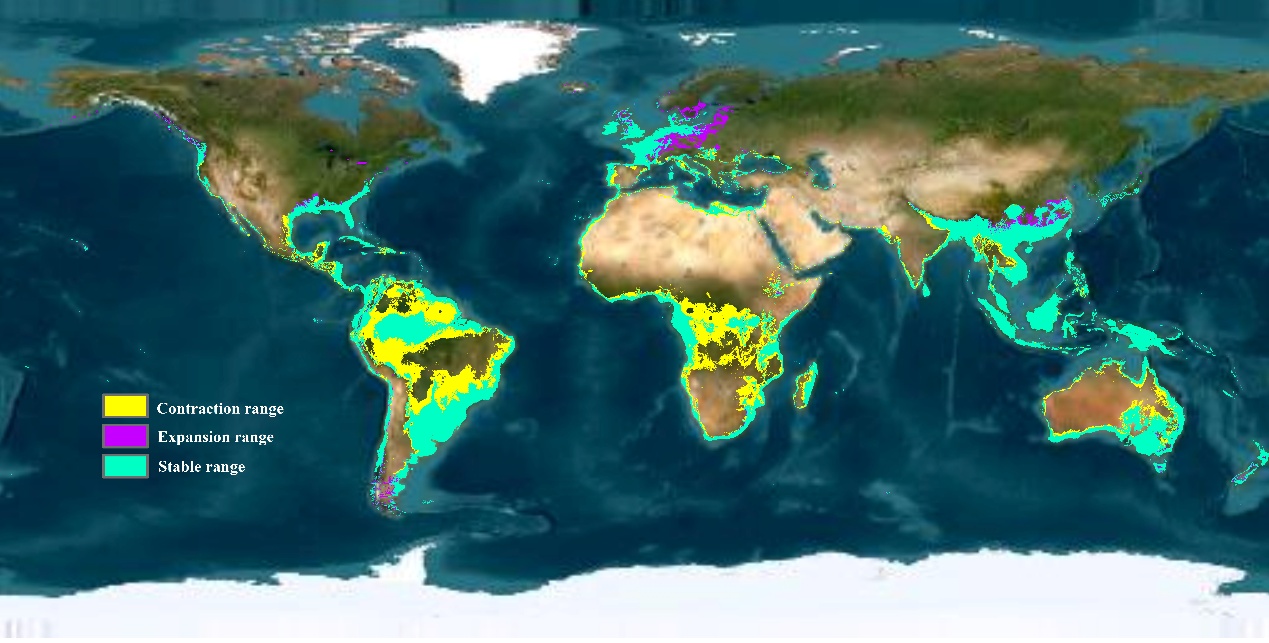
**

**D**

**
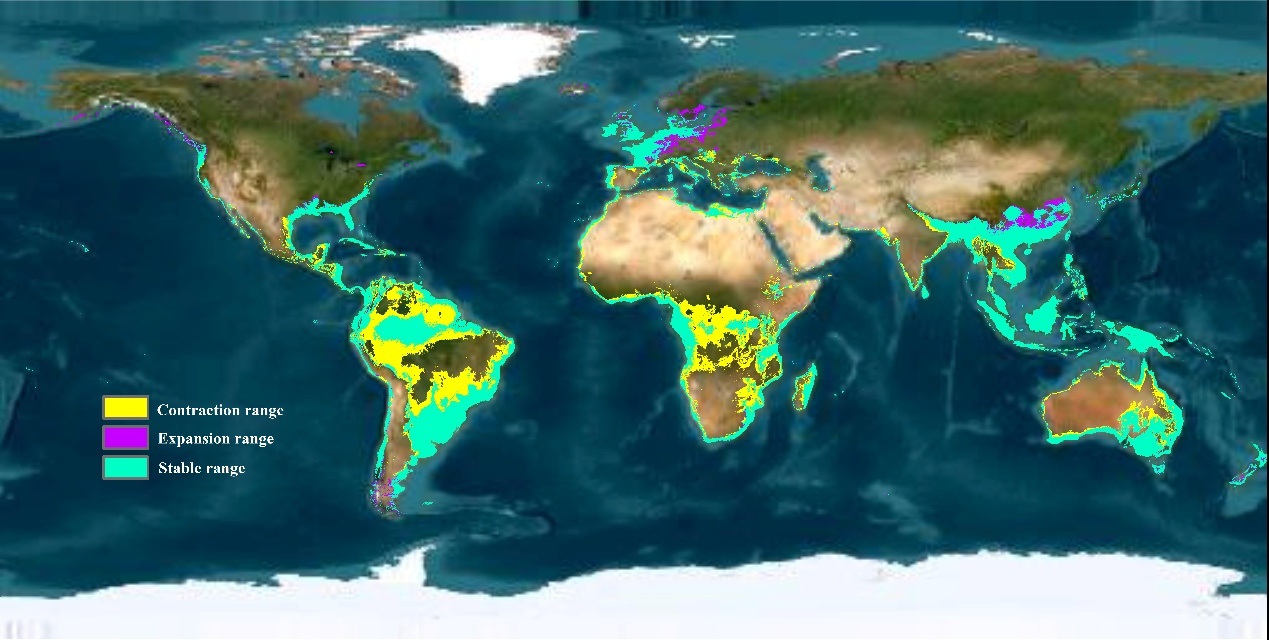
**

**E**

**
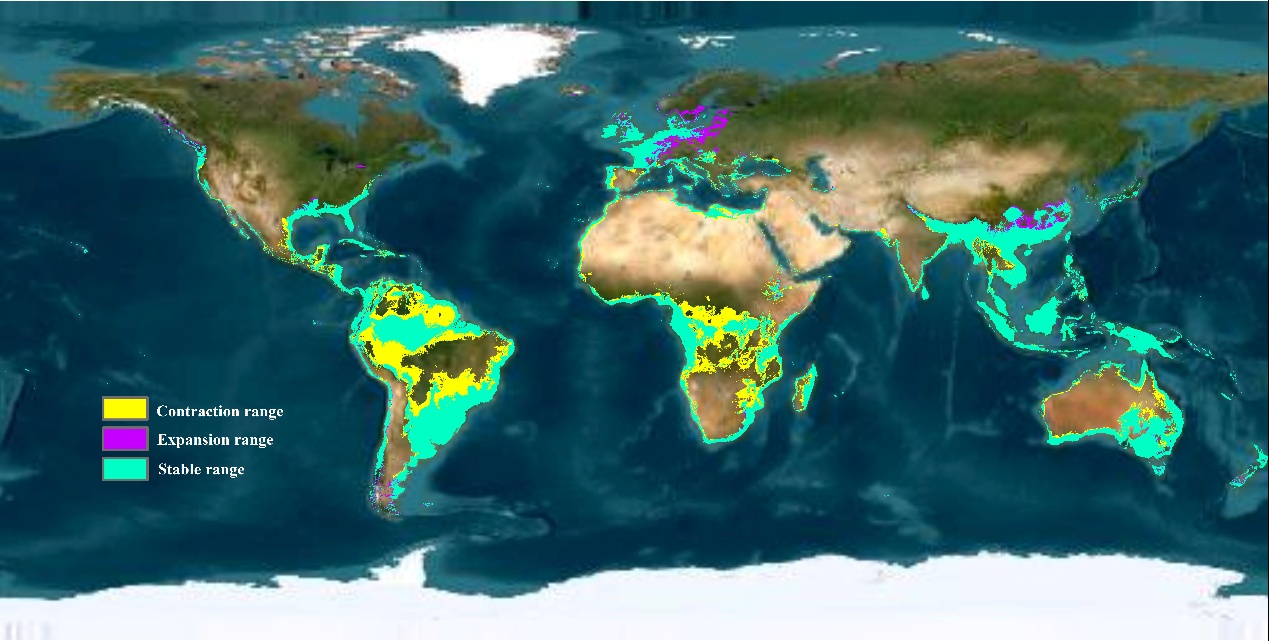
**

**F**

**
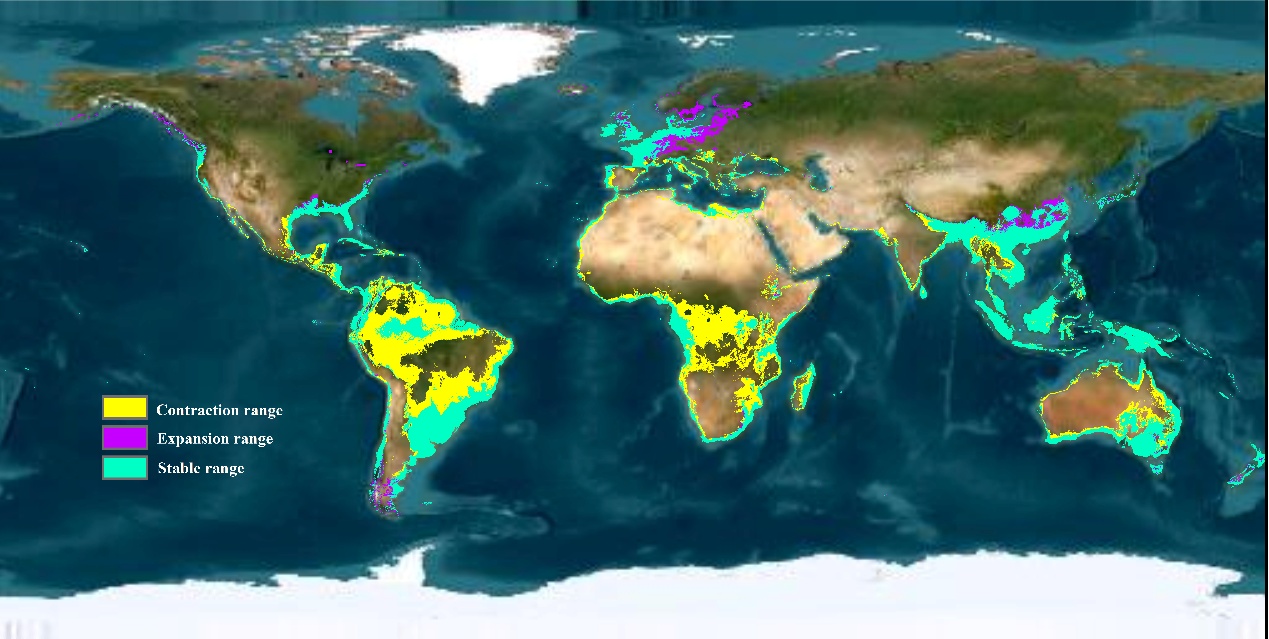
**

**G**

**
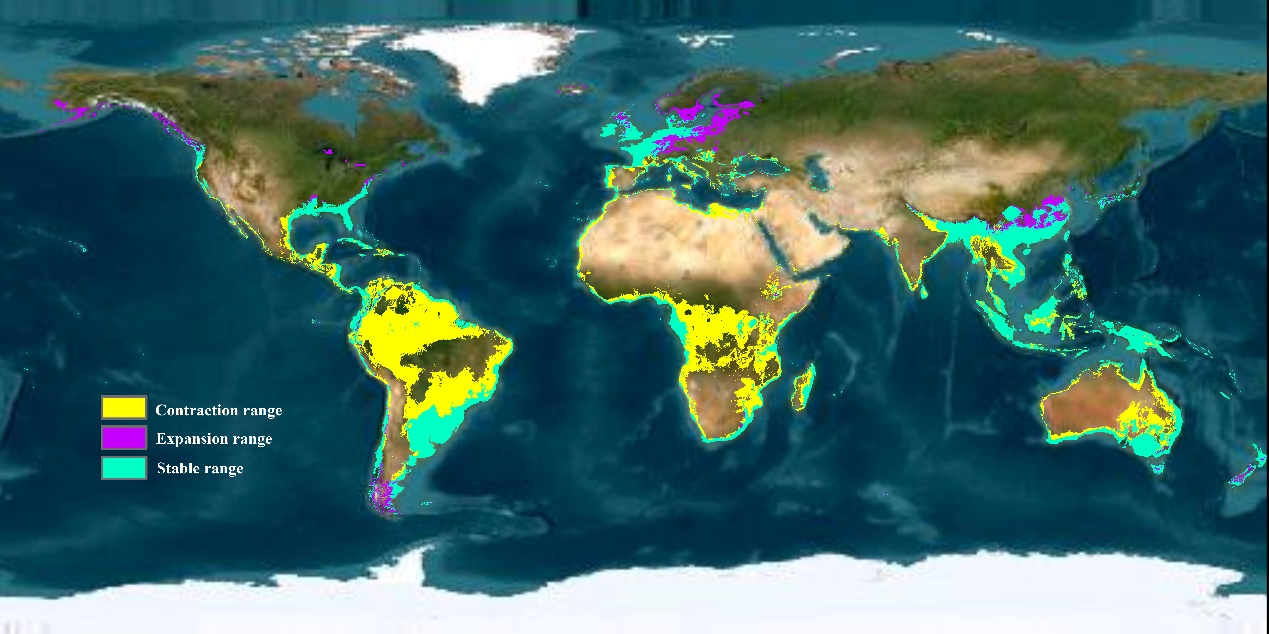
**

**H**

**
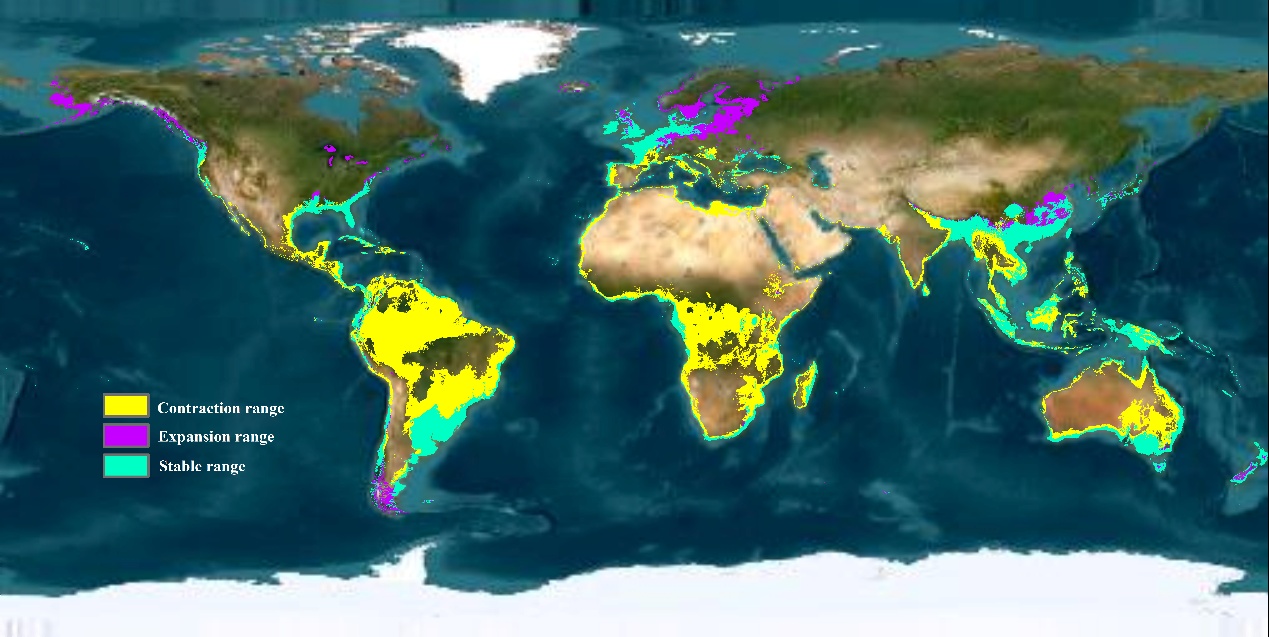
**

**(5) *Parthenolecanium corni* (PC)**


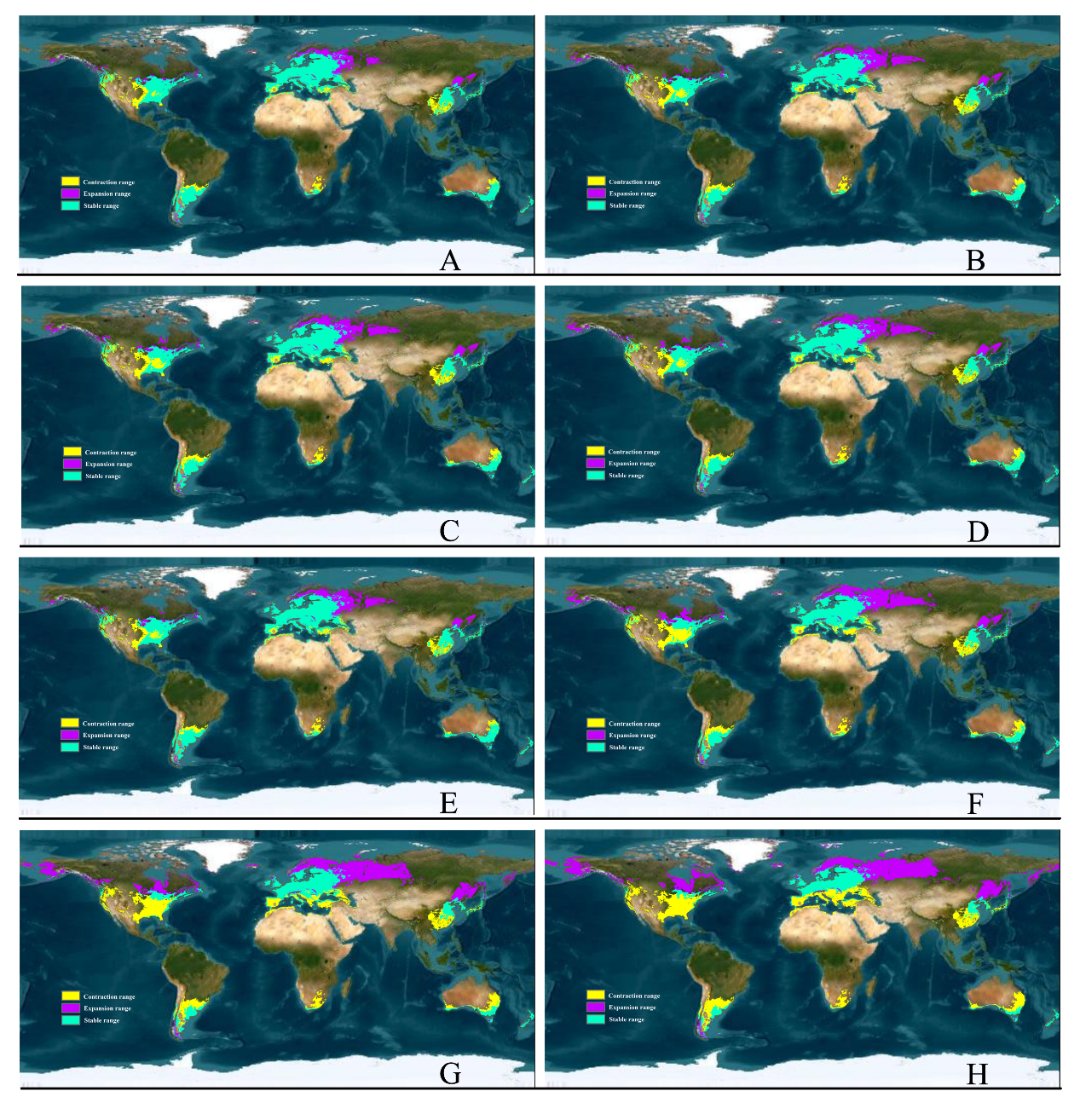


**A**

**
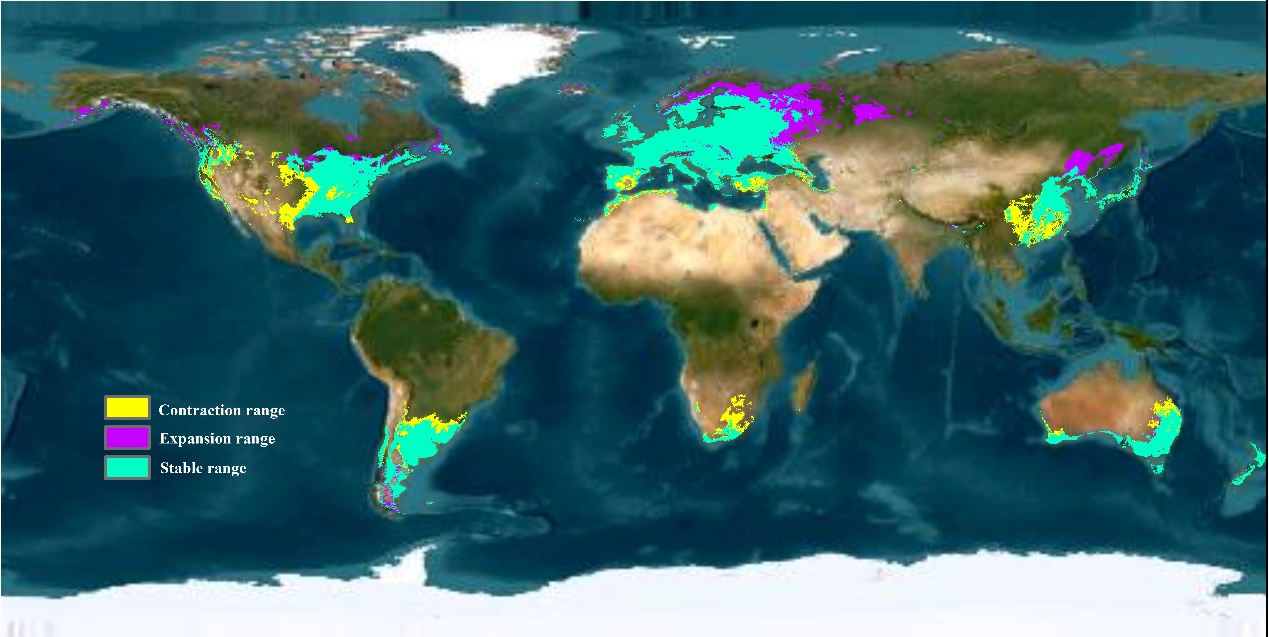
**

**B**

**
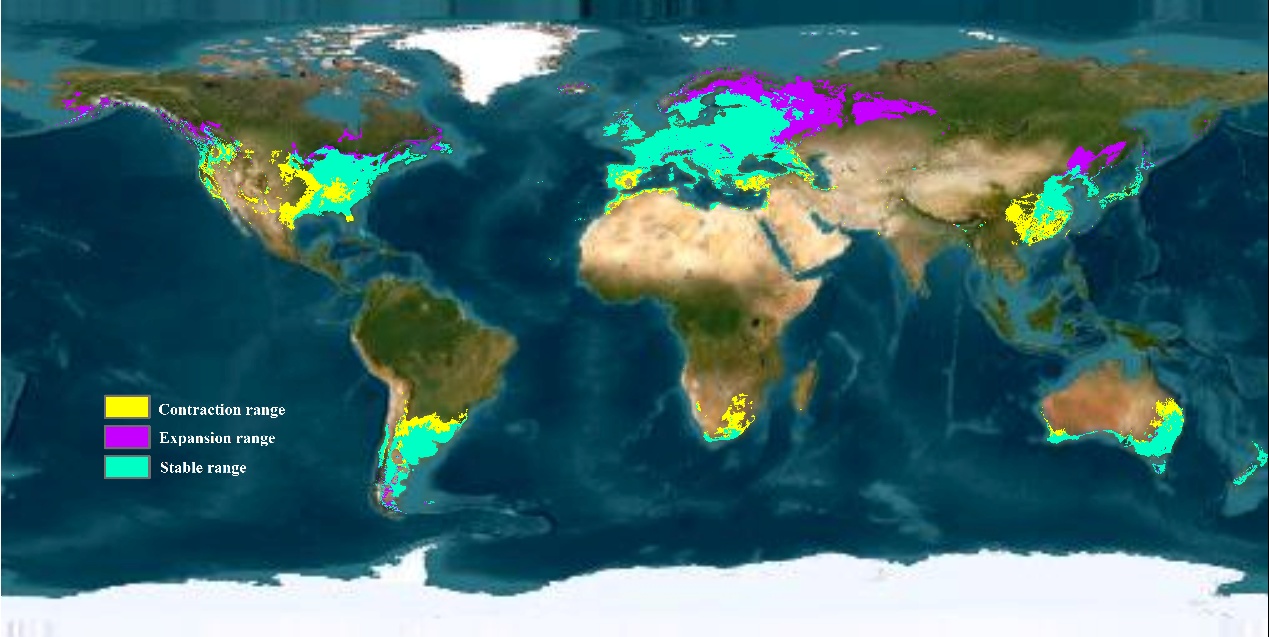
**

**C**

**
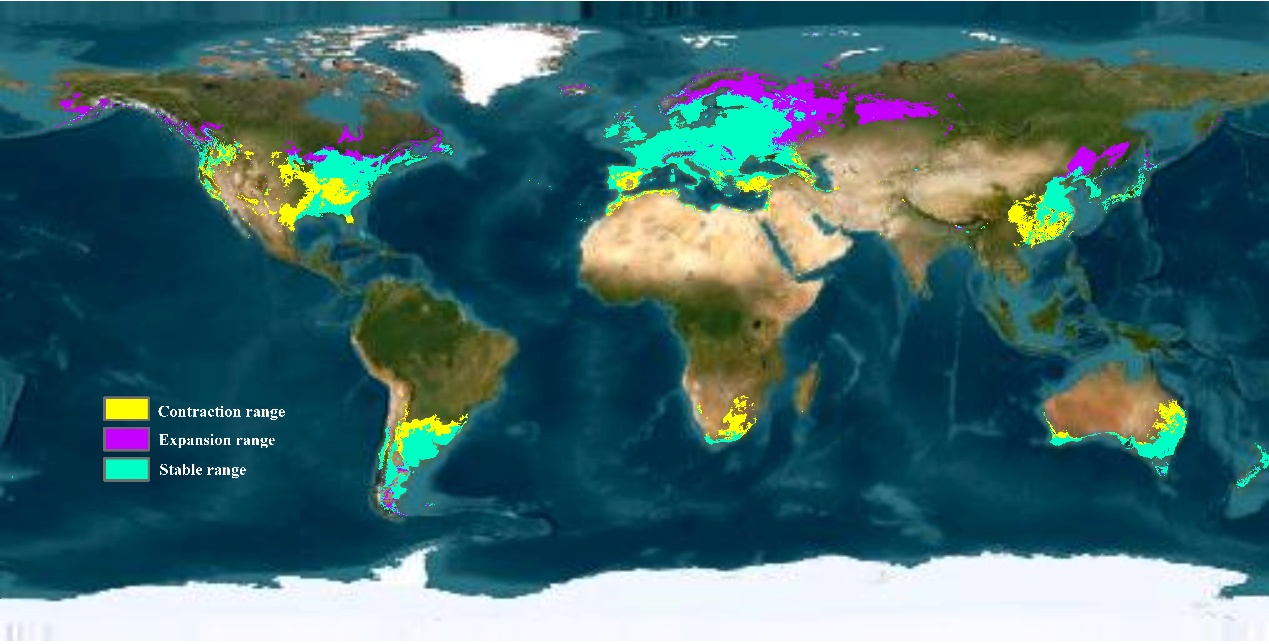
**

**D**

**
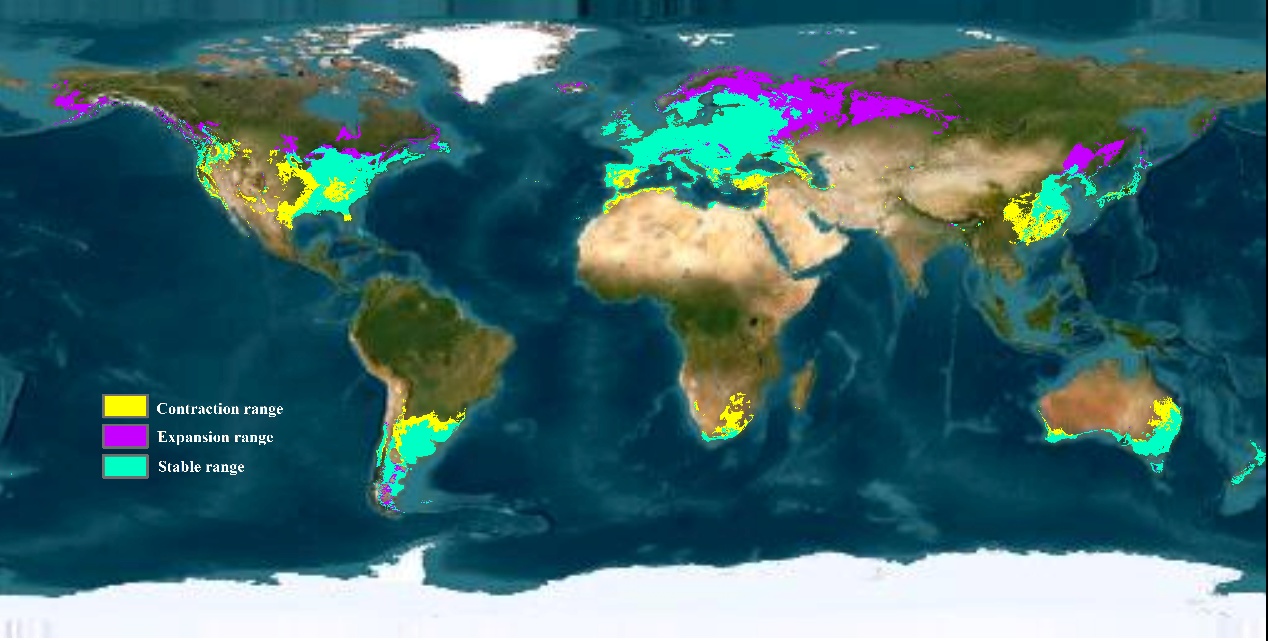
**

**E**

**
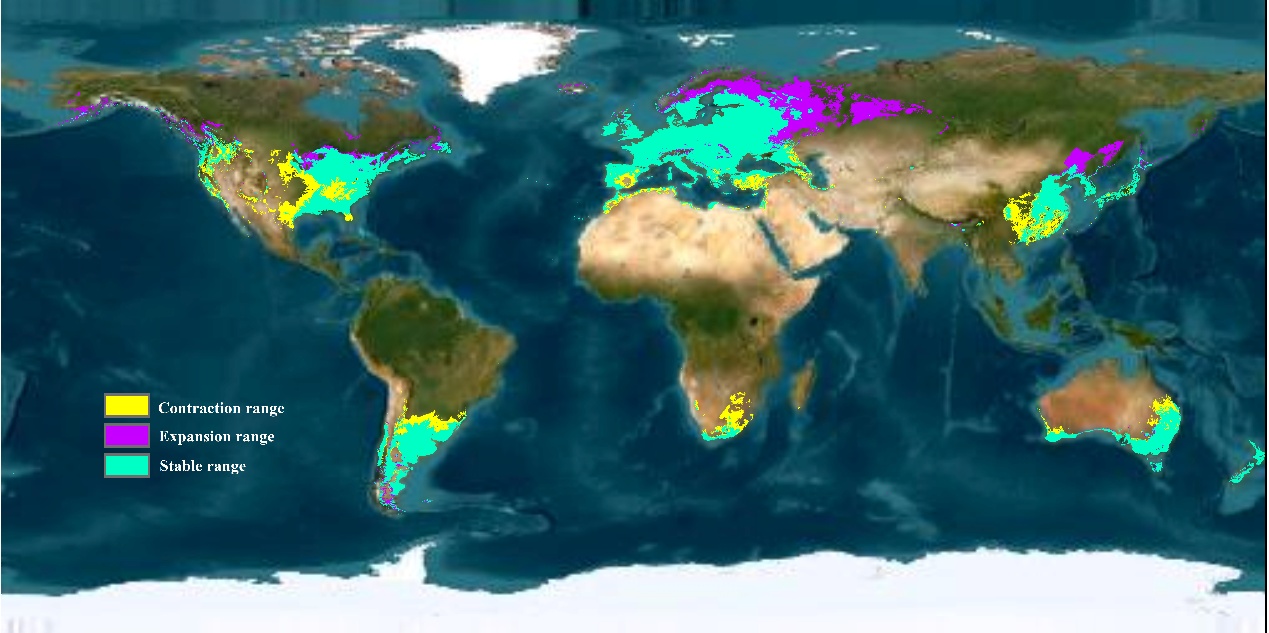
**

**F**

**
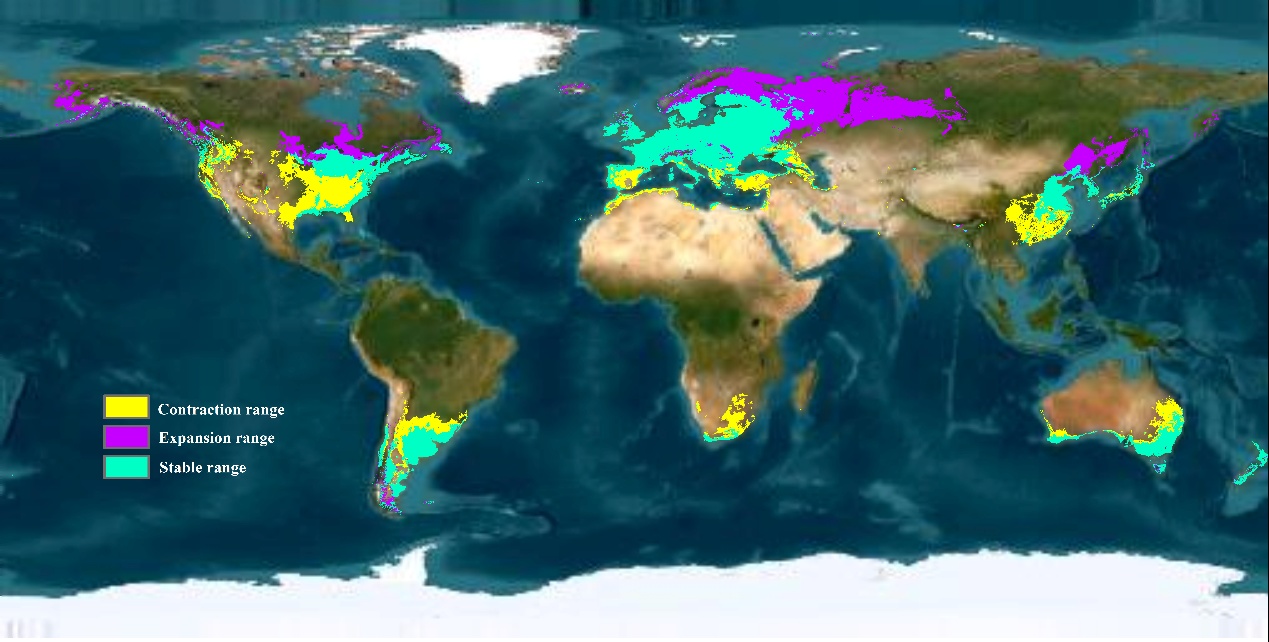
**

**G**

**
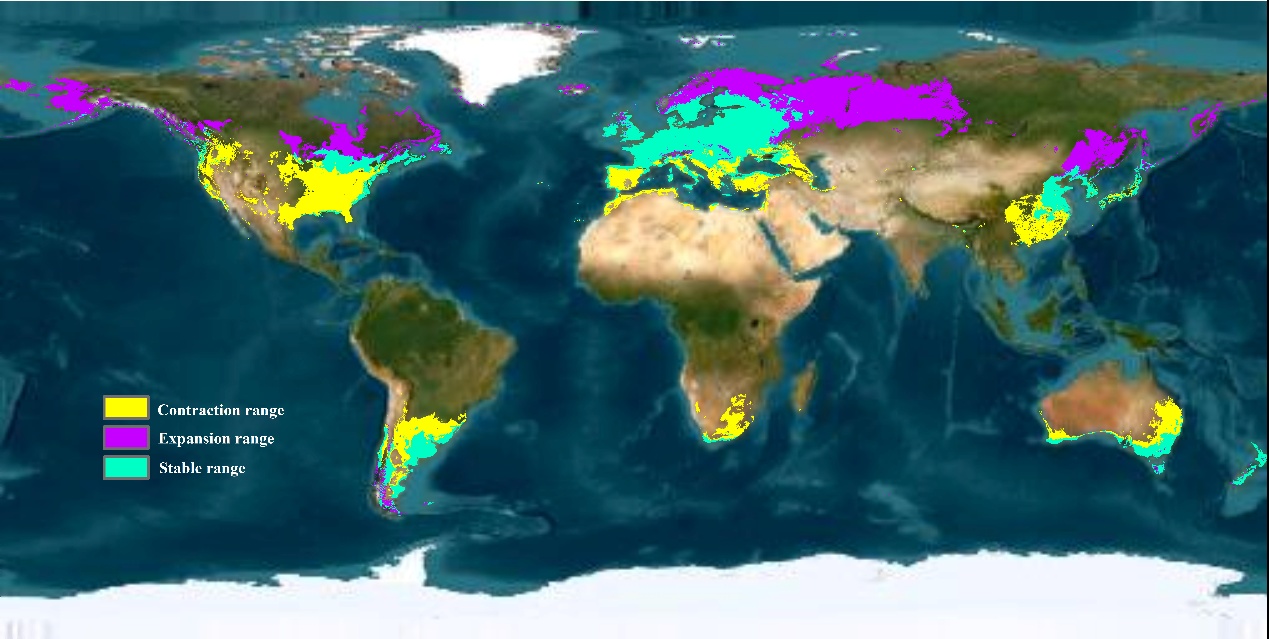
**

**H**

**
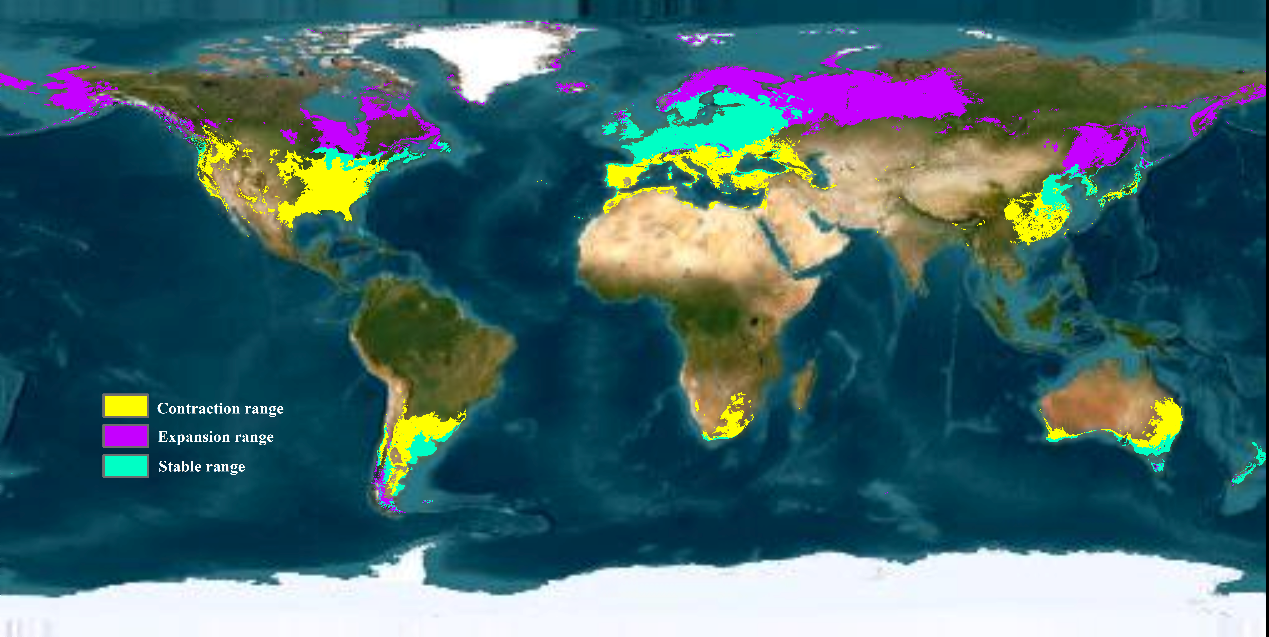
**

**(6) *Parthenolecanium persicae* (PP)**


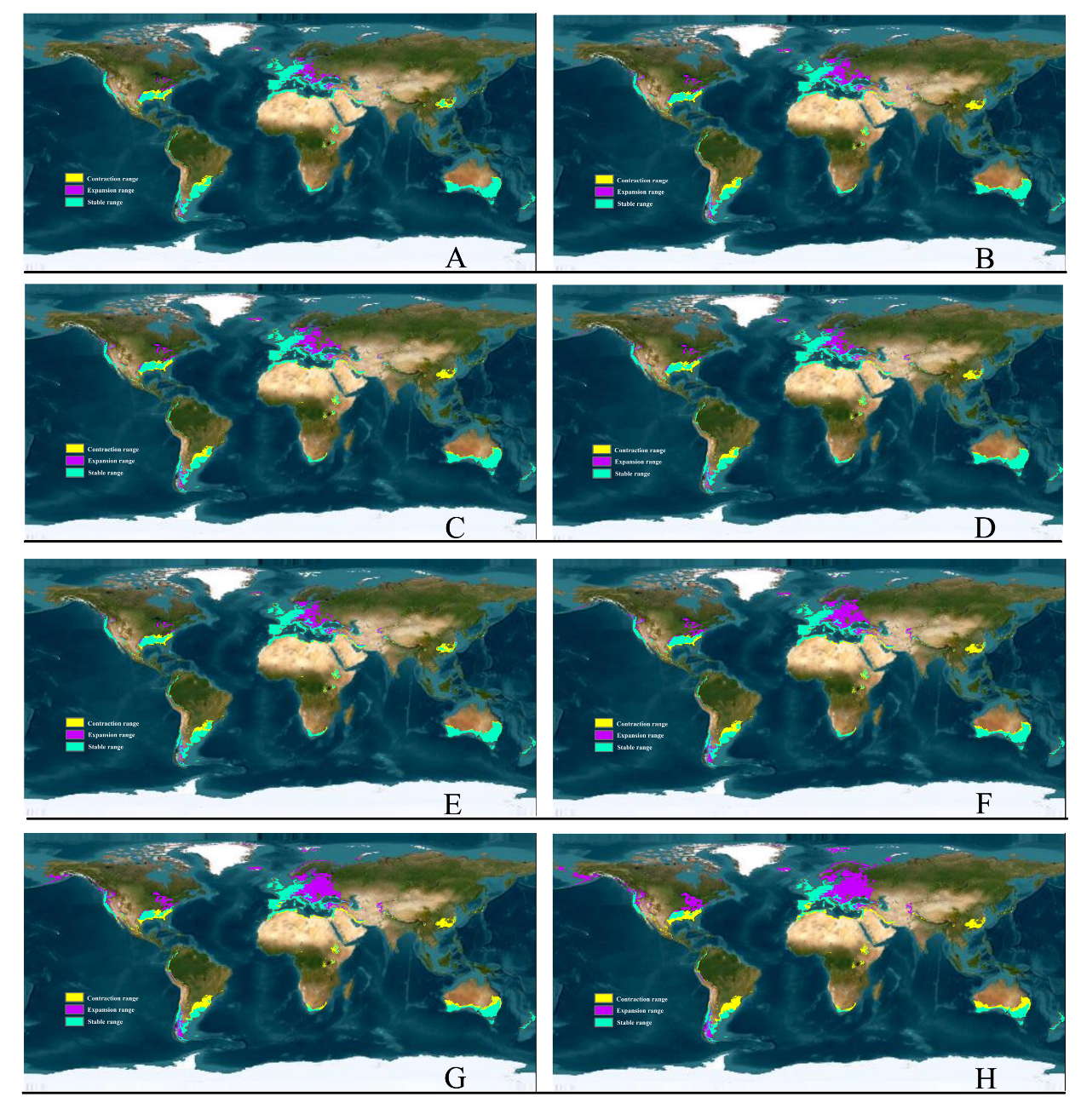


**A**

**
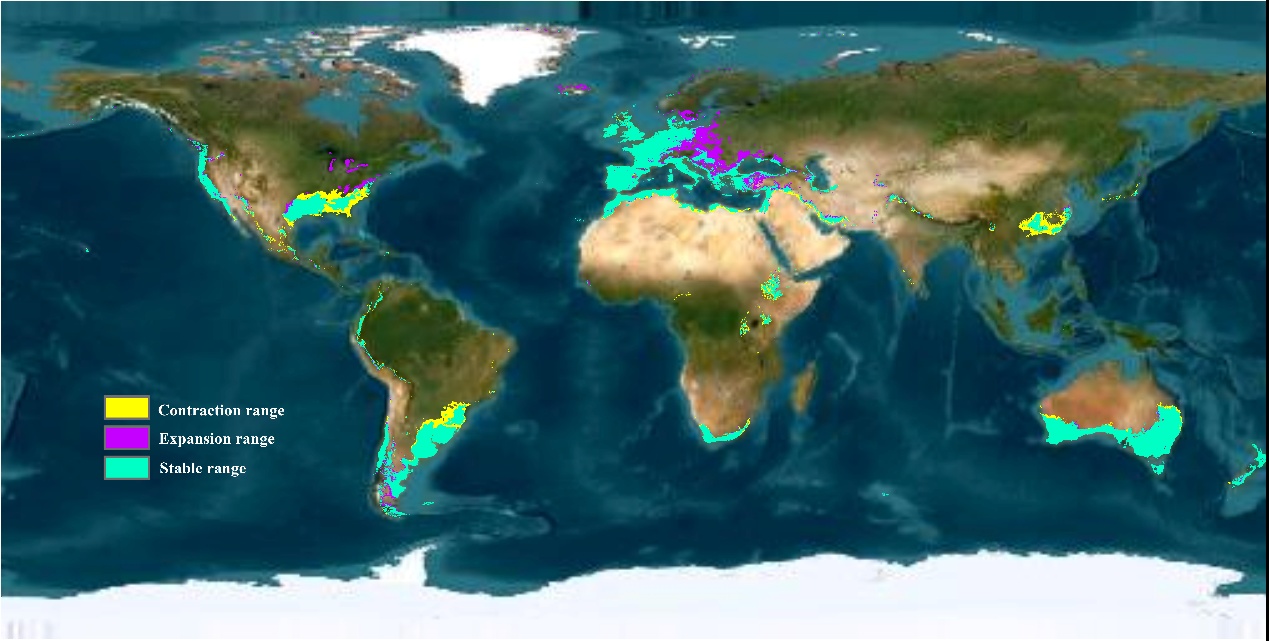
**

**B**

**
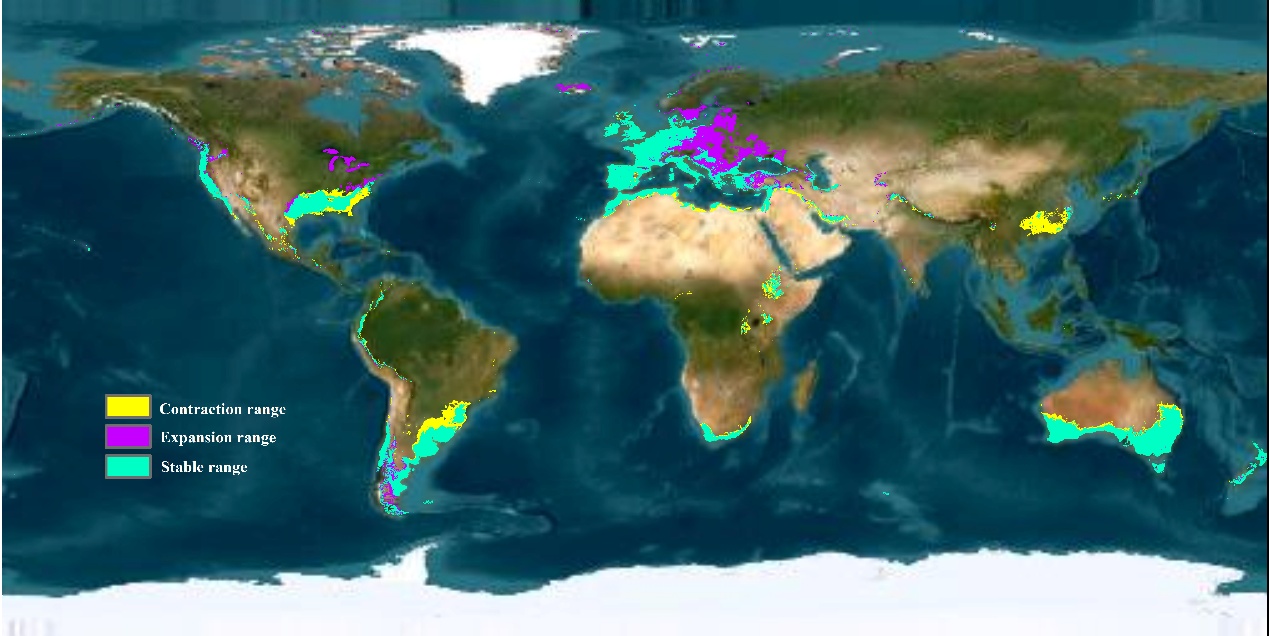
**

**C**

**
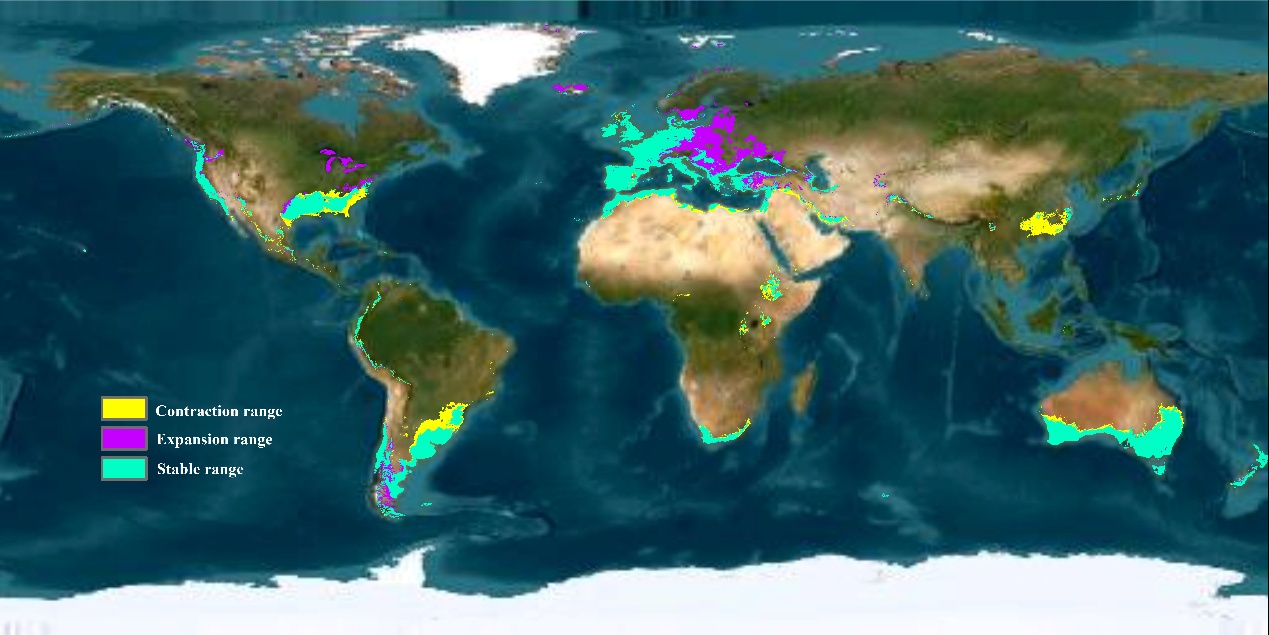
**

**D**

**
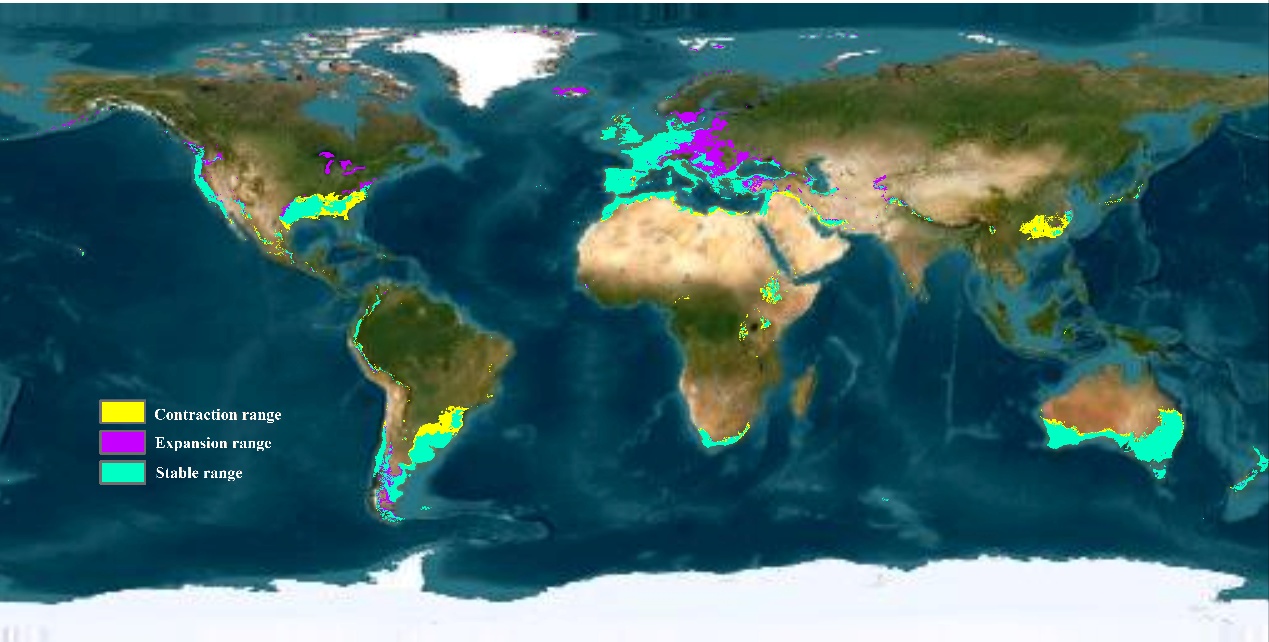
**

**E**

**
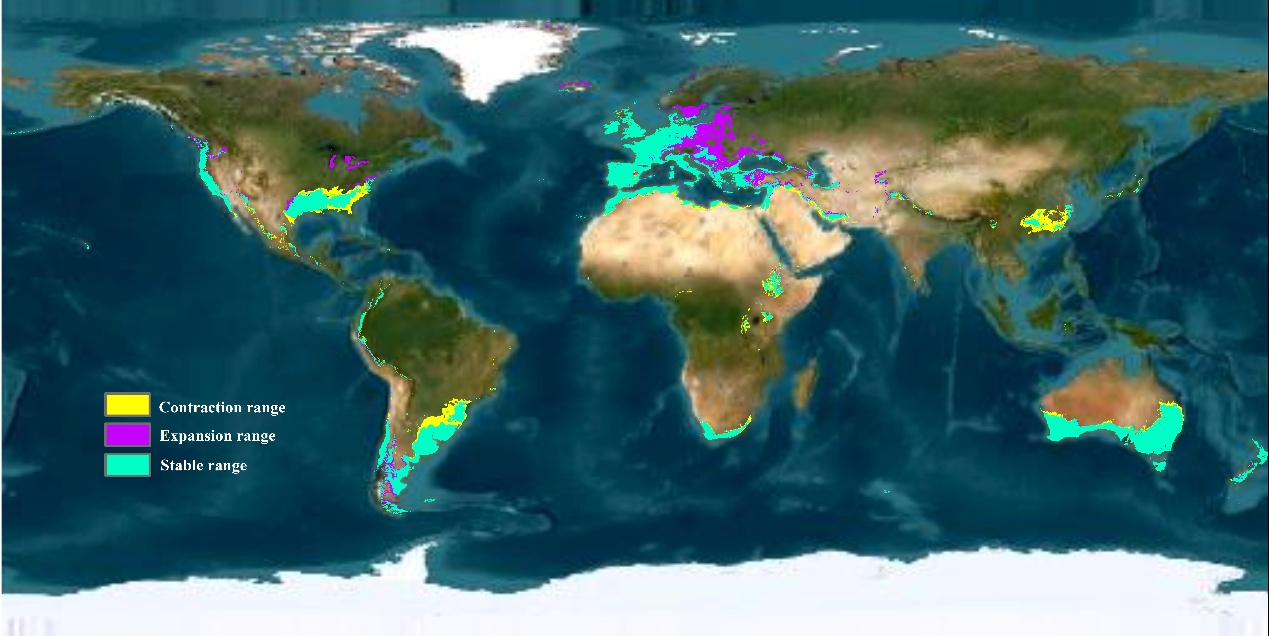
**

**F**

**
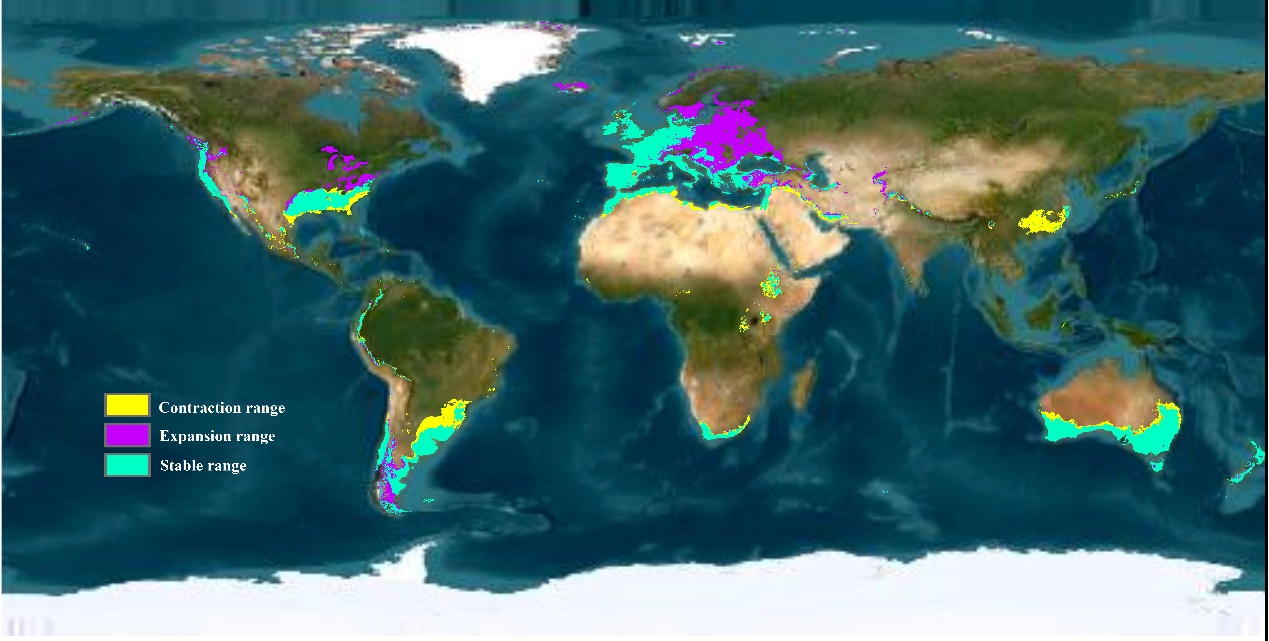
**

**G**

**
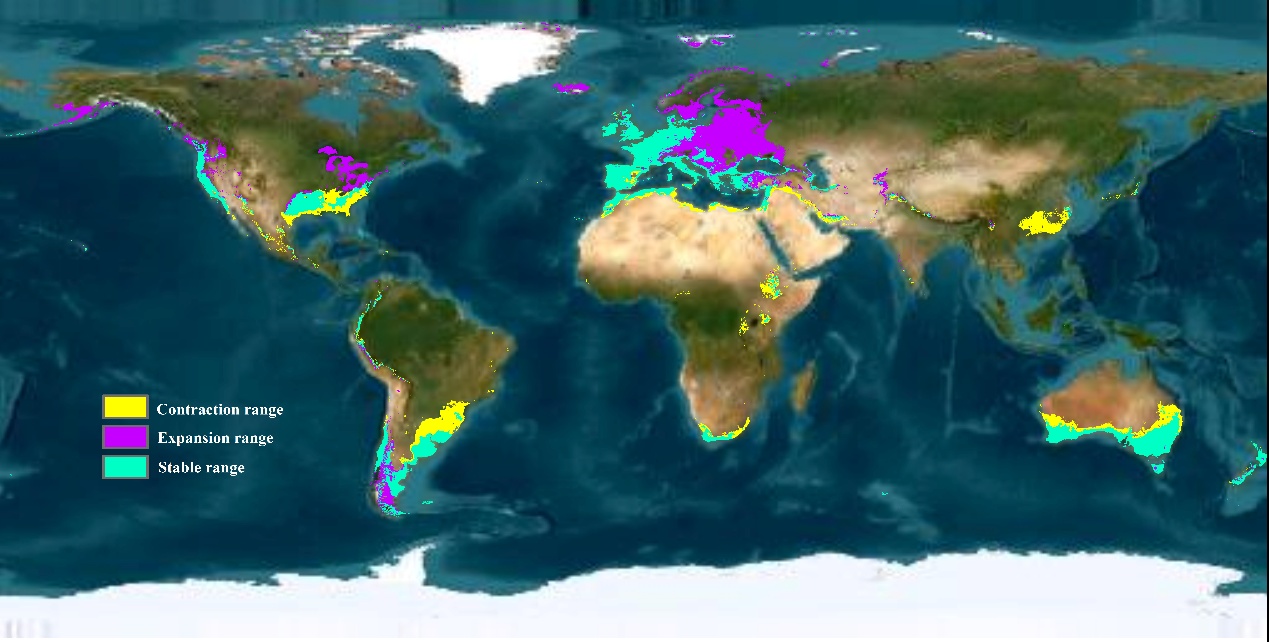
**

**H**

**
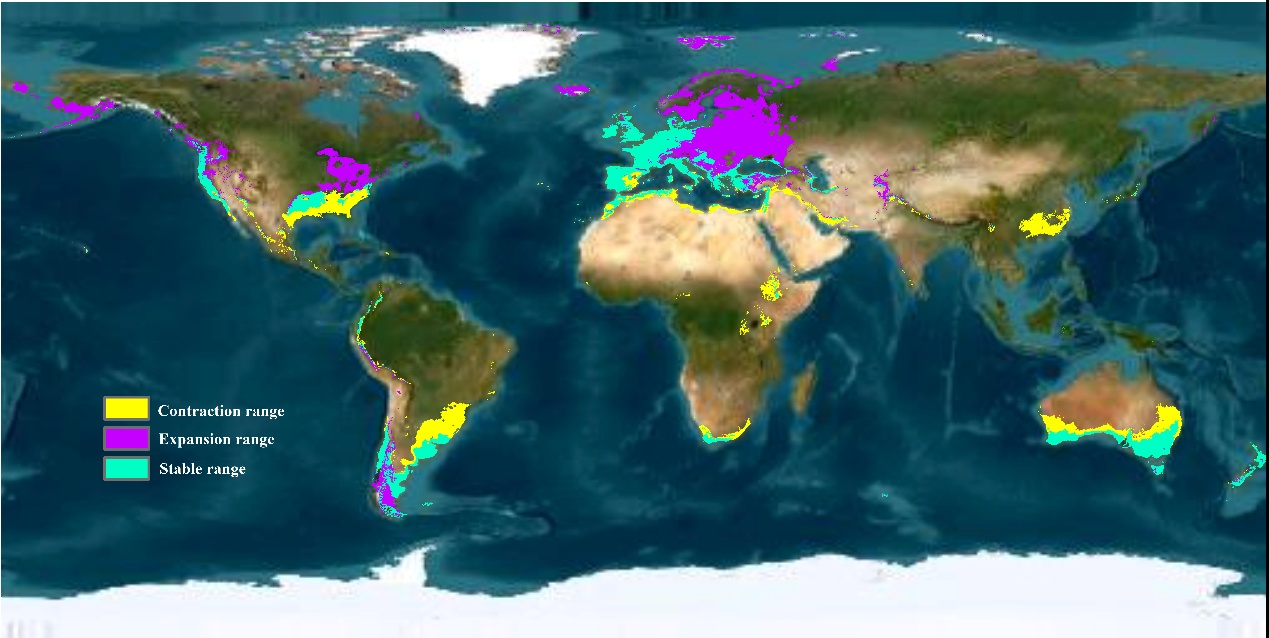
**

**(7) *Pulvinaria vitis* (PV)**


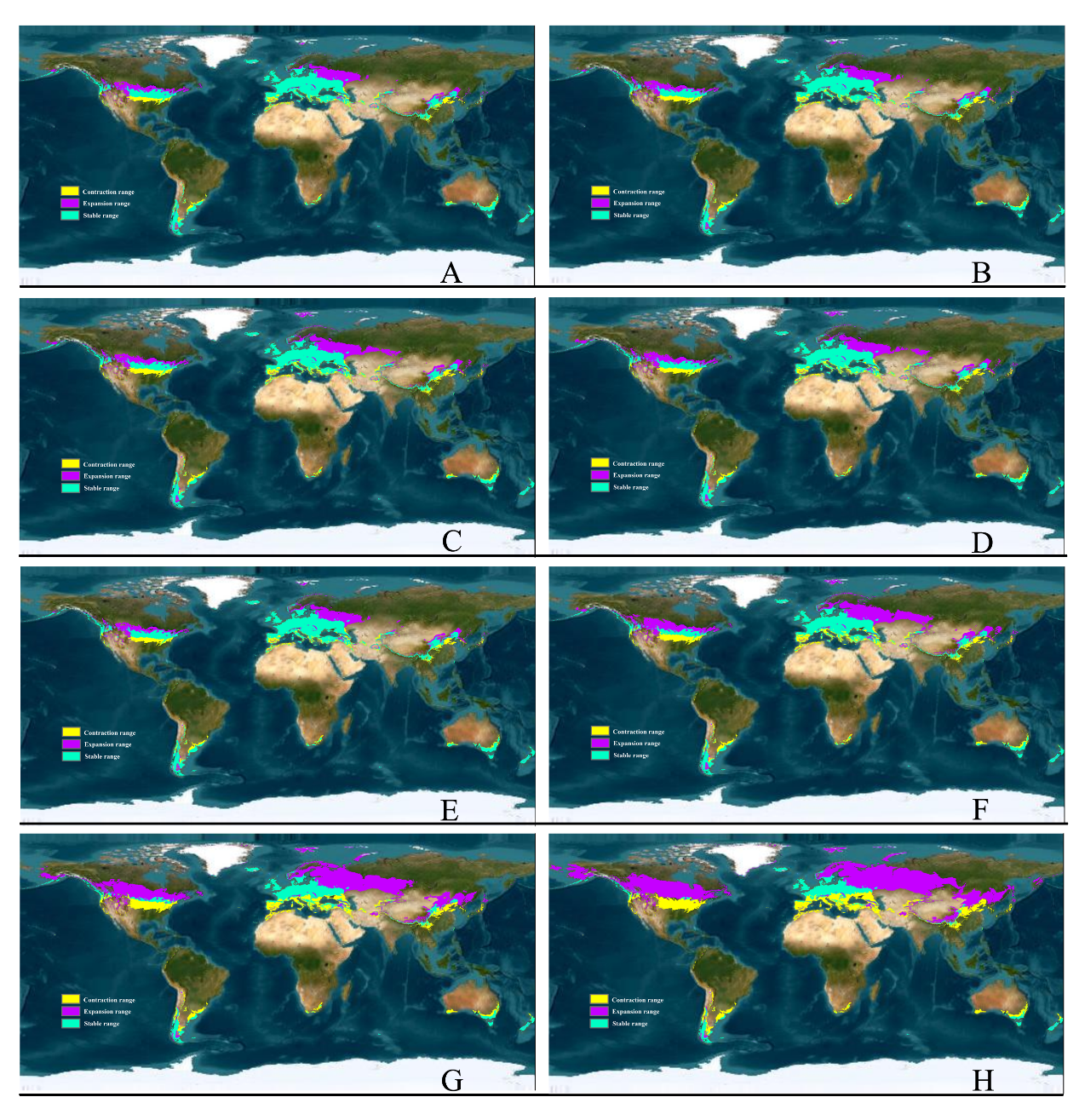


**A**

**
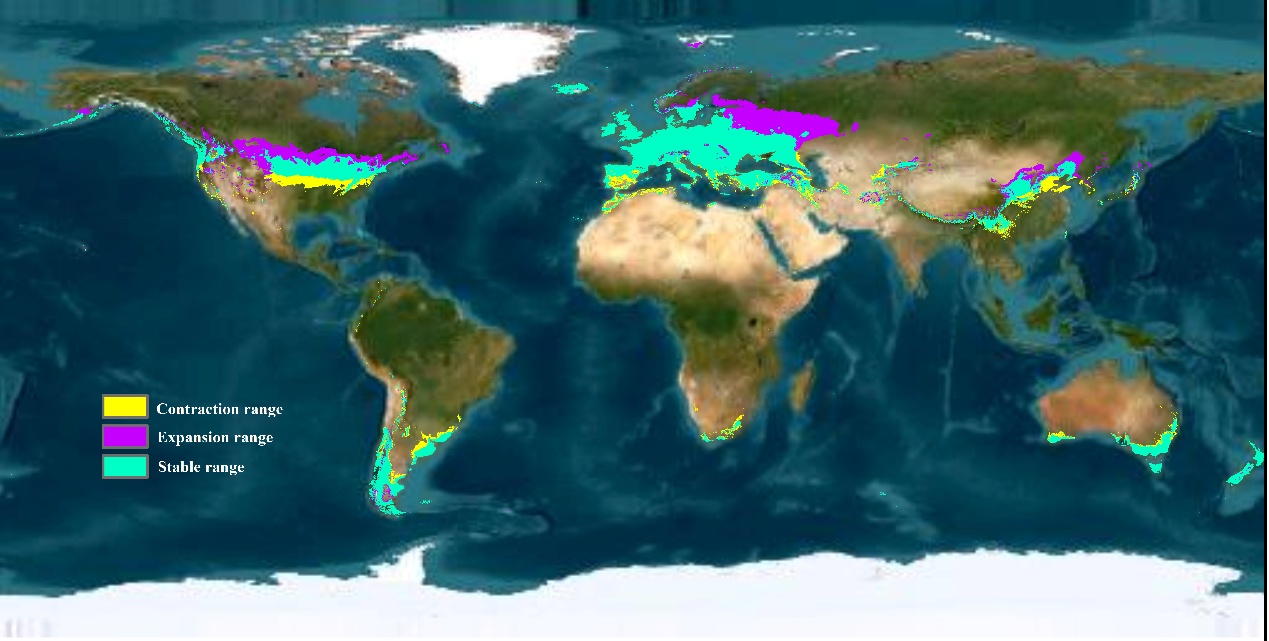
**

**B**

**
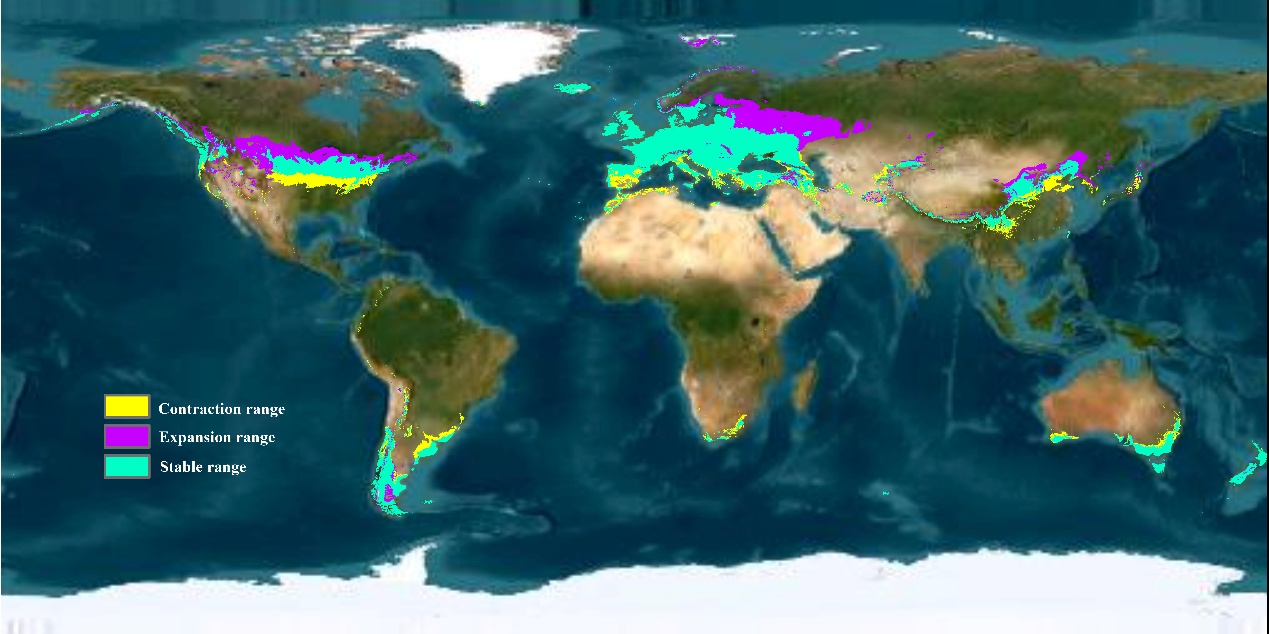
**

**C**

**
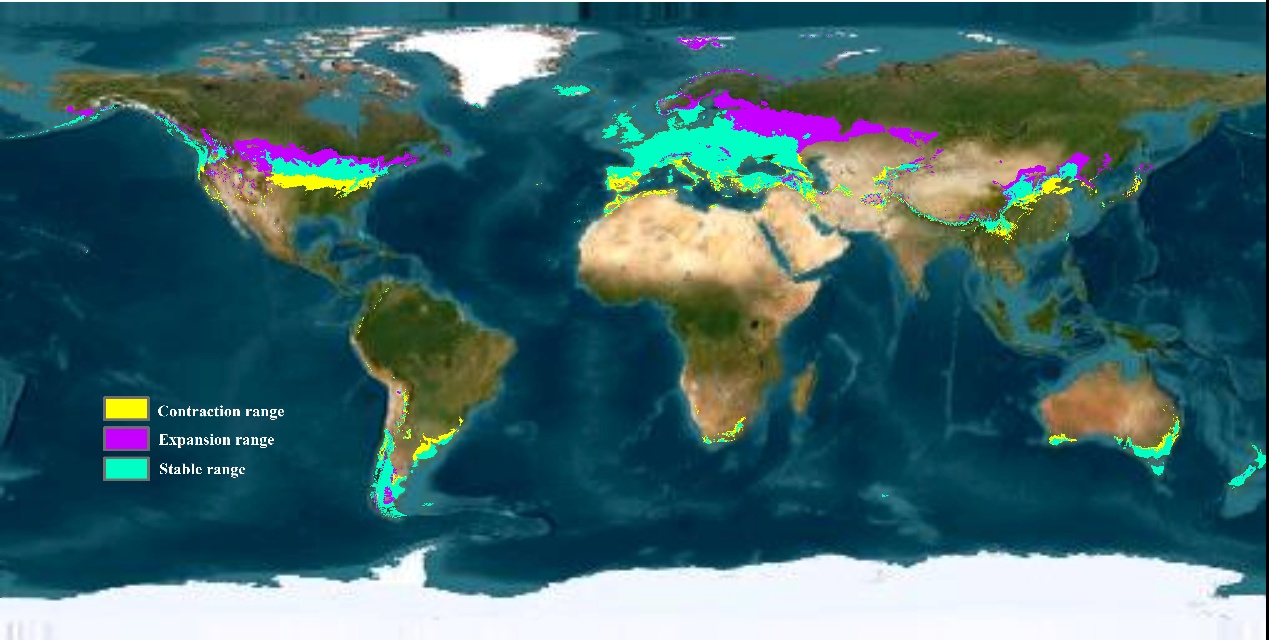
**

**D**

**
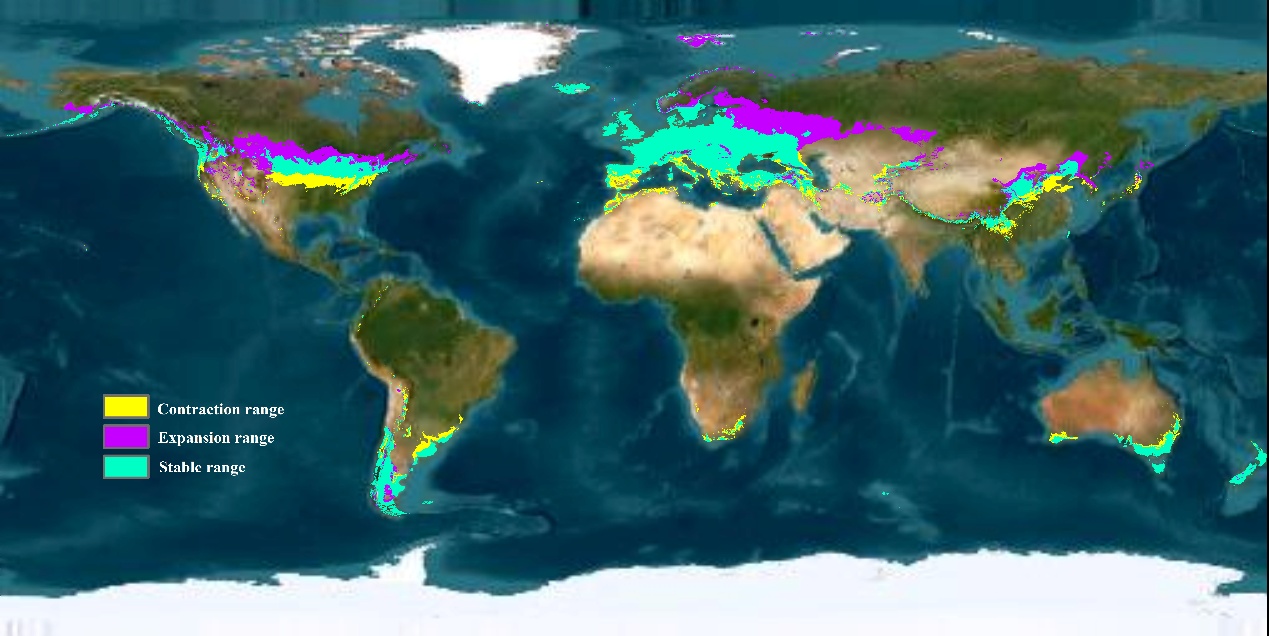
**

**E**

**
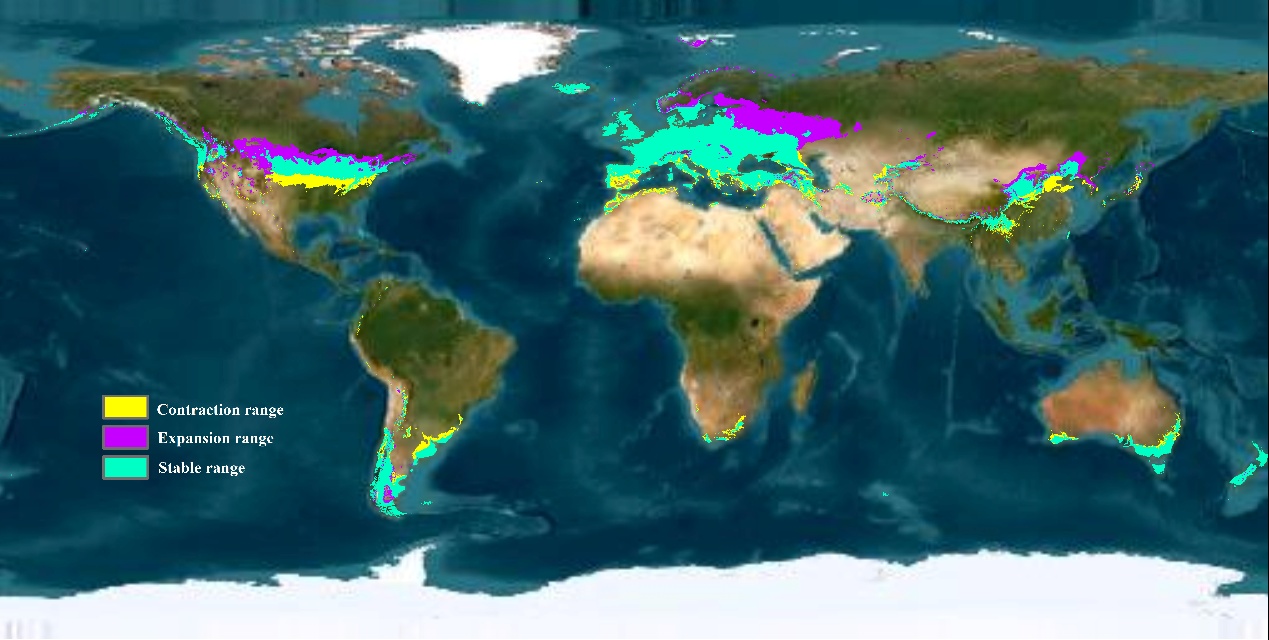
**

**F**

**
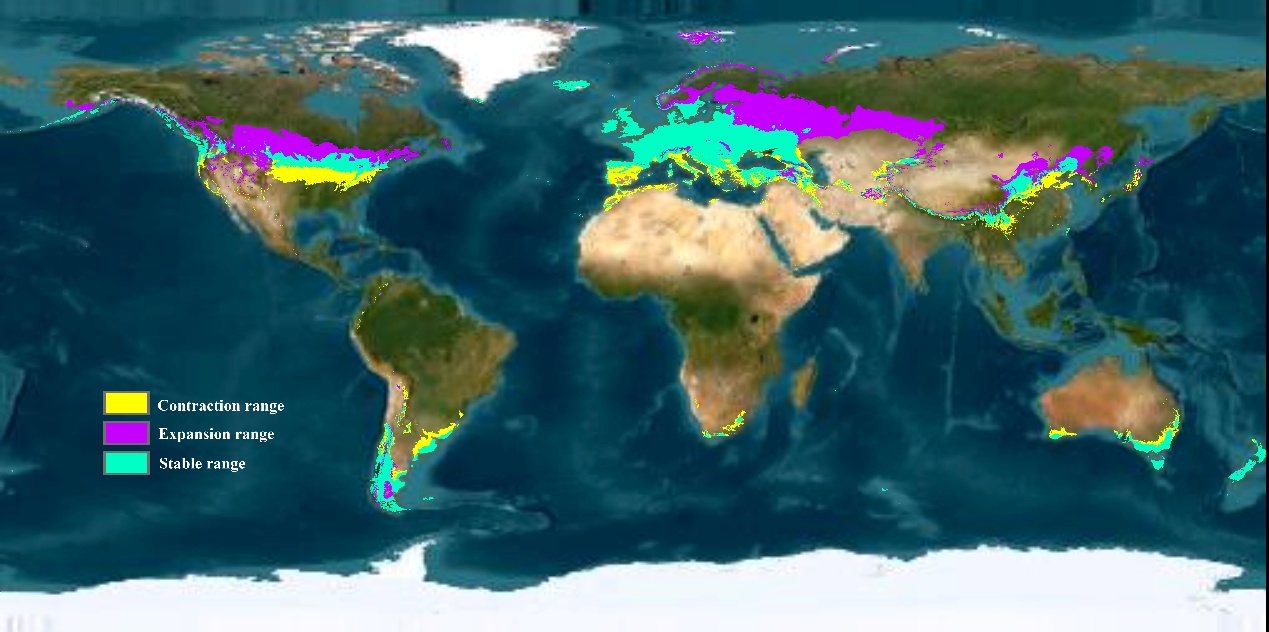
**

**G**

**
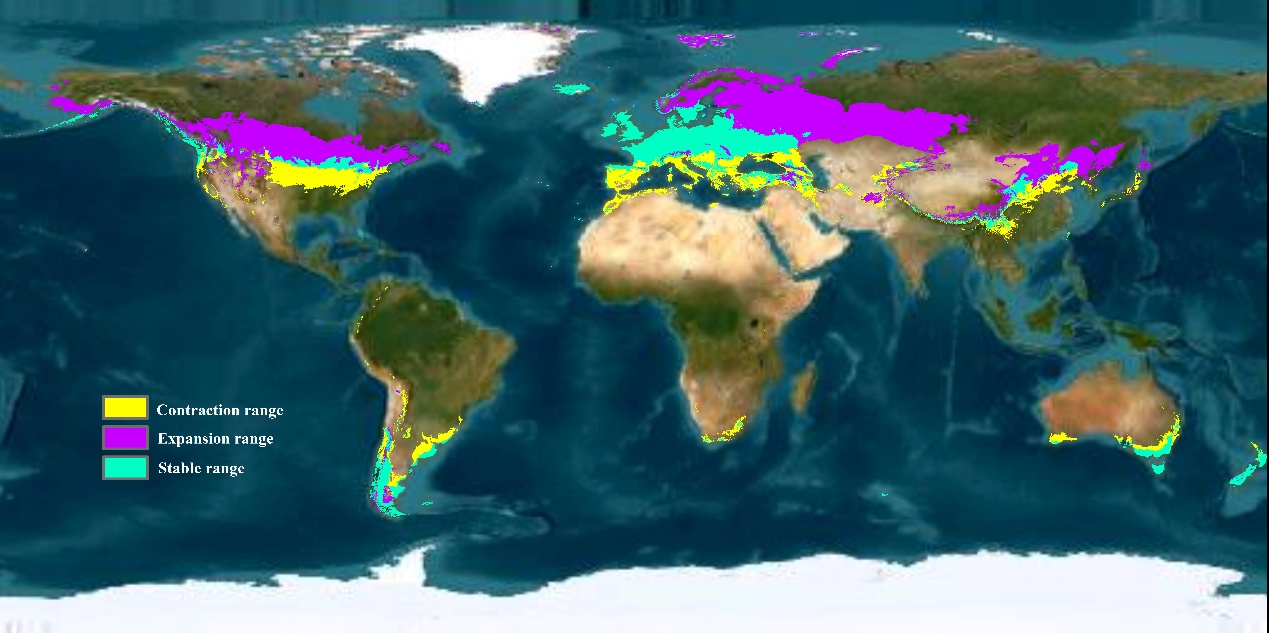
**

**H**

**
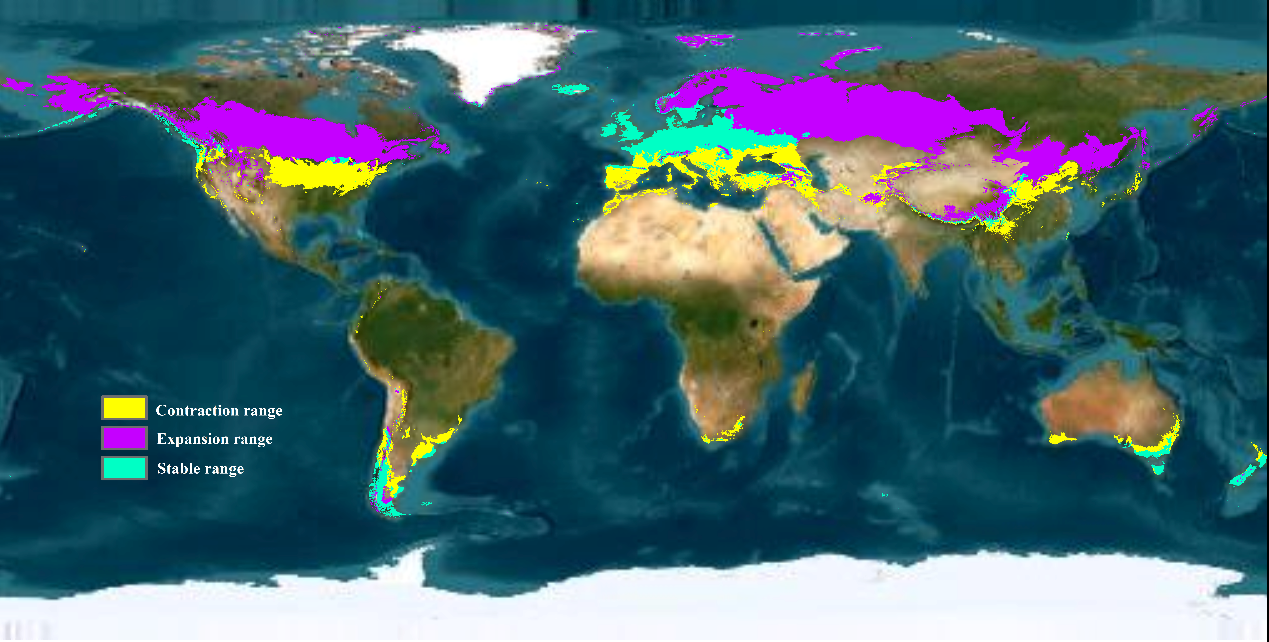
**
