# Supplementary material for: Climate Change Drives the Distribution of Insect Vectors for GLRaV‐3 on a Global Scale
Source: Ecol Evol. 2025 Oct 14;15(10):e72297. doi: 10.1002/ece3.72297 (PMC12521802; doi:10.1002/ece3.72297)

**Fig. S4.** The potential distribution region of seven insect vectors under current study

**(1) *Ceroplastes rusci* (CR)**

**
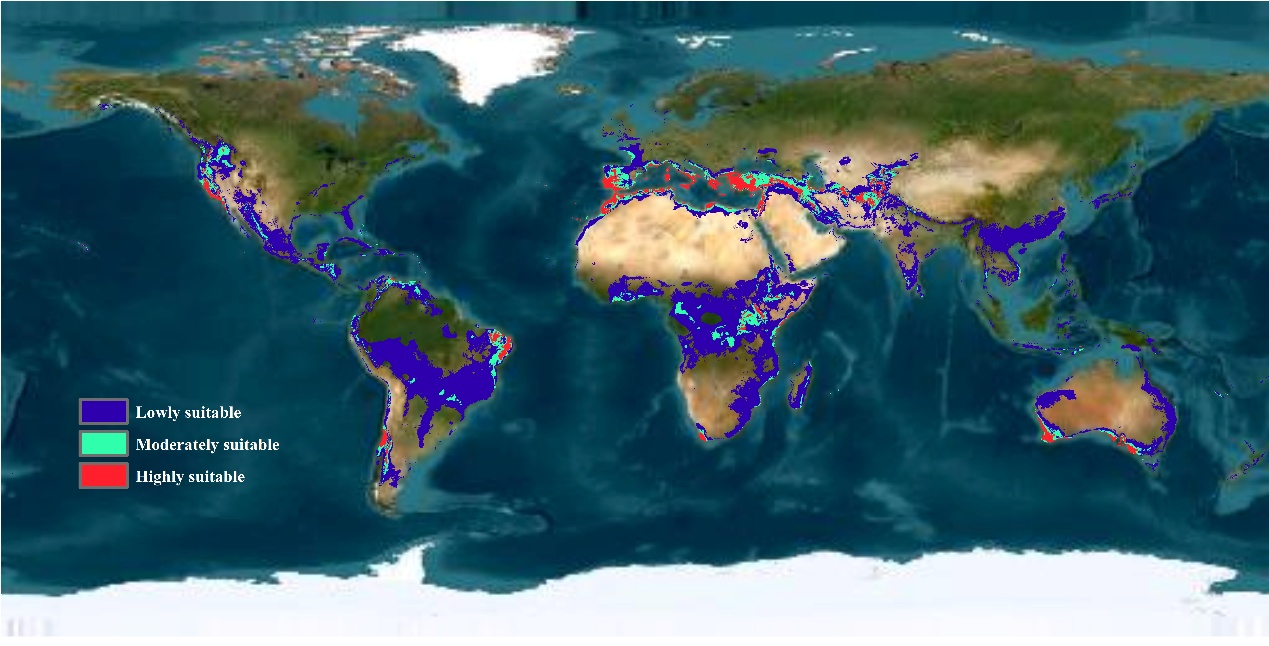
**

**(2) *Coccus longulus* (CL)**

**
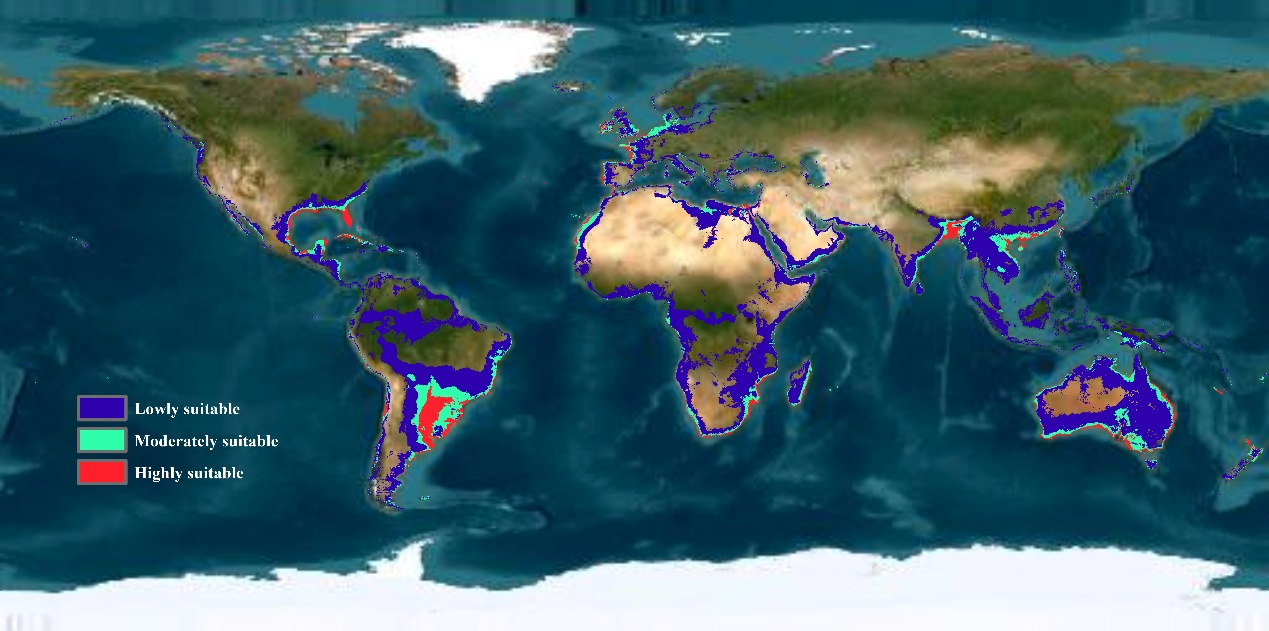
**

**(3) *Neopulvinaria innumerabilis* (NI)**

**
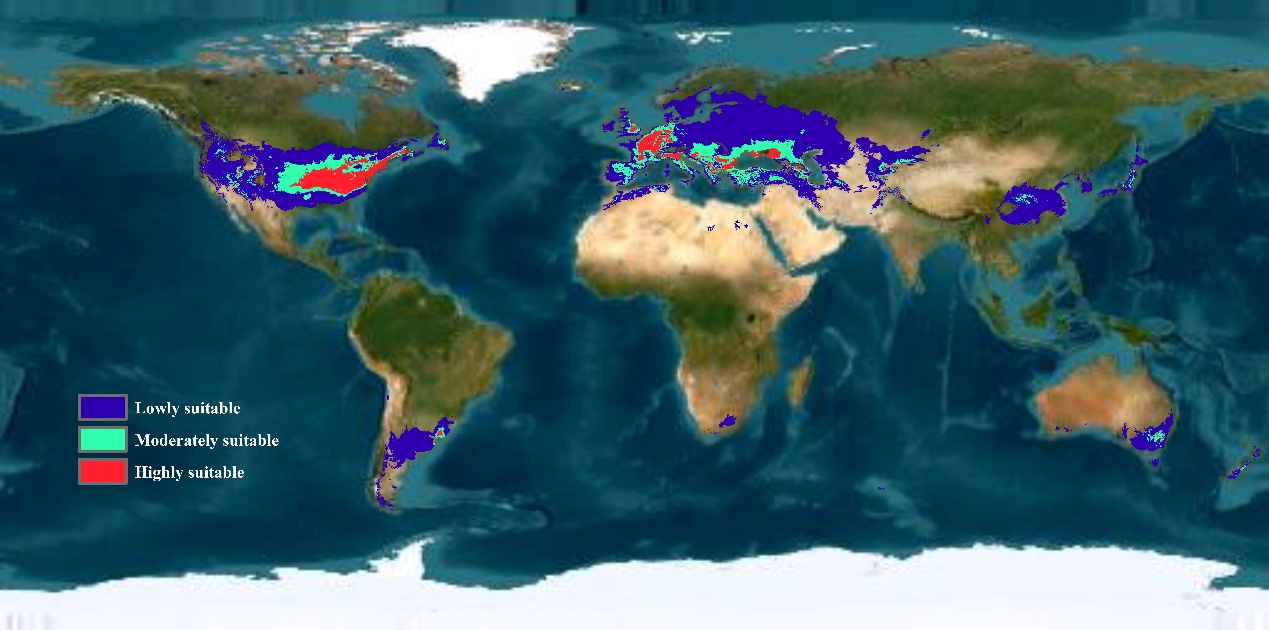
**

**(4) *Parasaissetia nigra* (PN)**

**
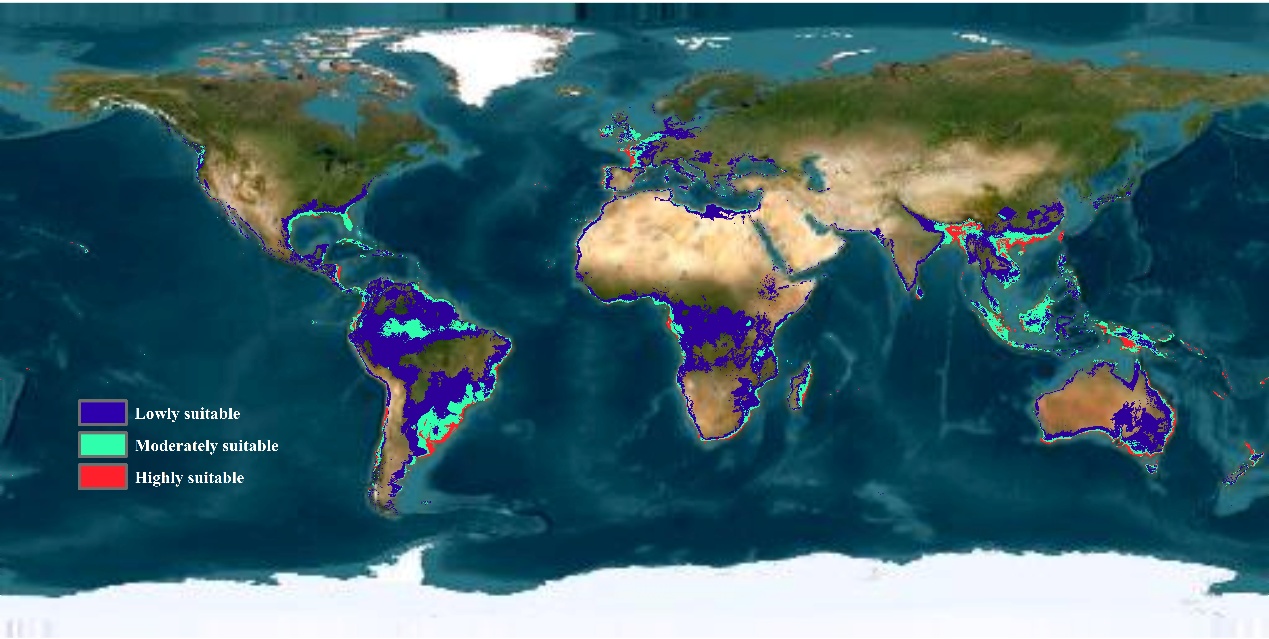
**

**(5) *Parthenolecanium corni* (PC)**

**
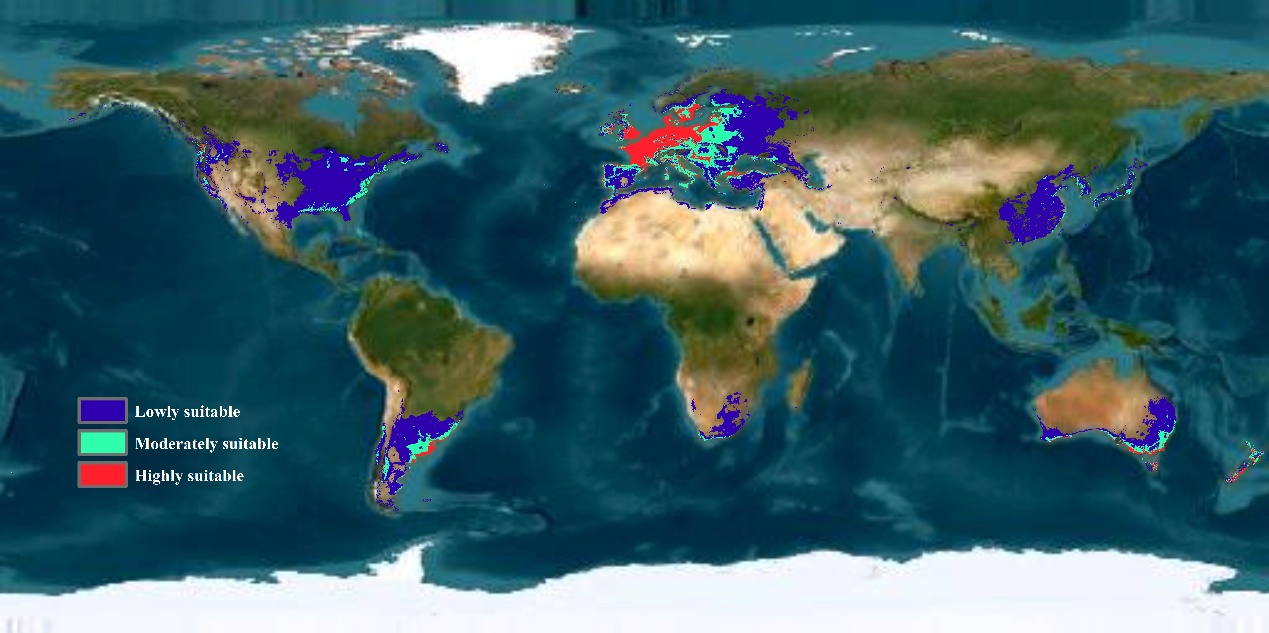
**

**(6)** ***Parthenolecanium persicae* (PP)**

**
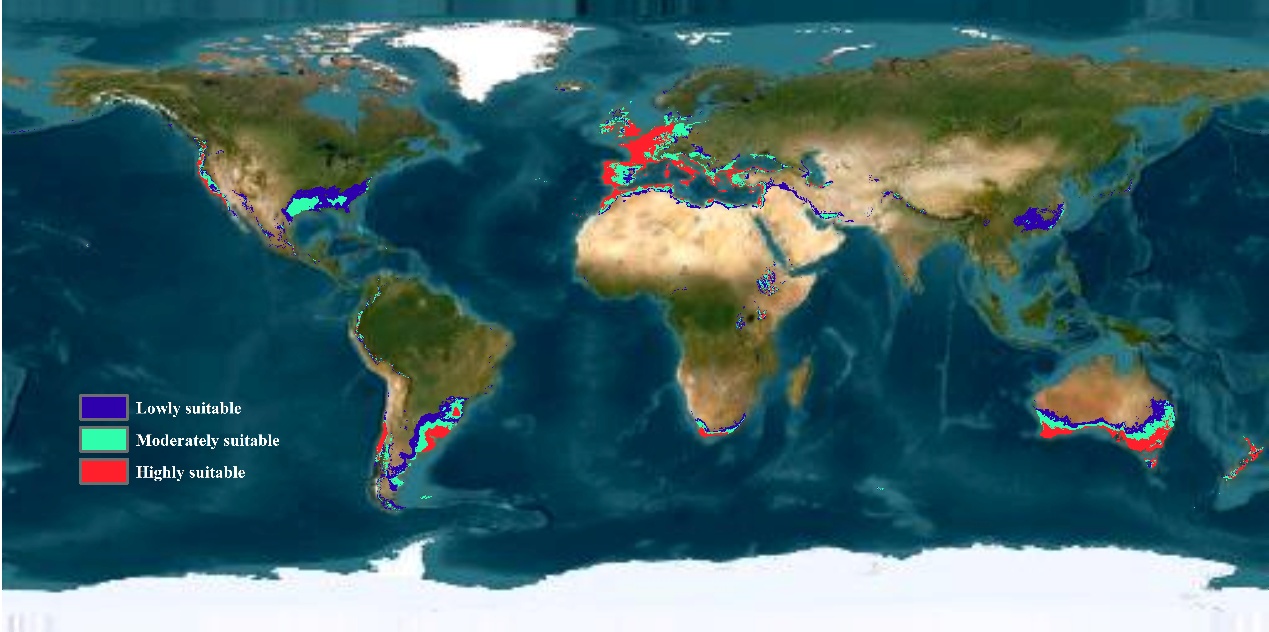
**

**(7) *Pulvinaria vitis* (PV)**


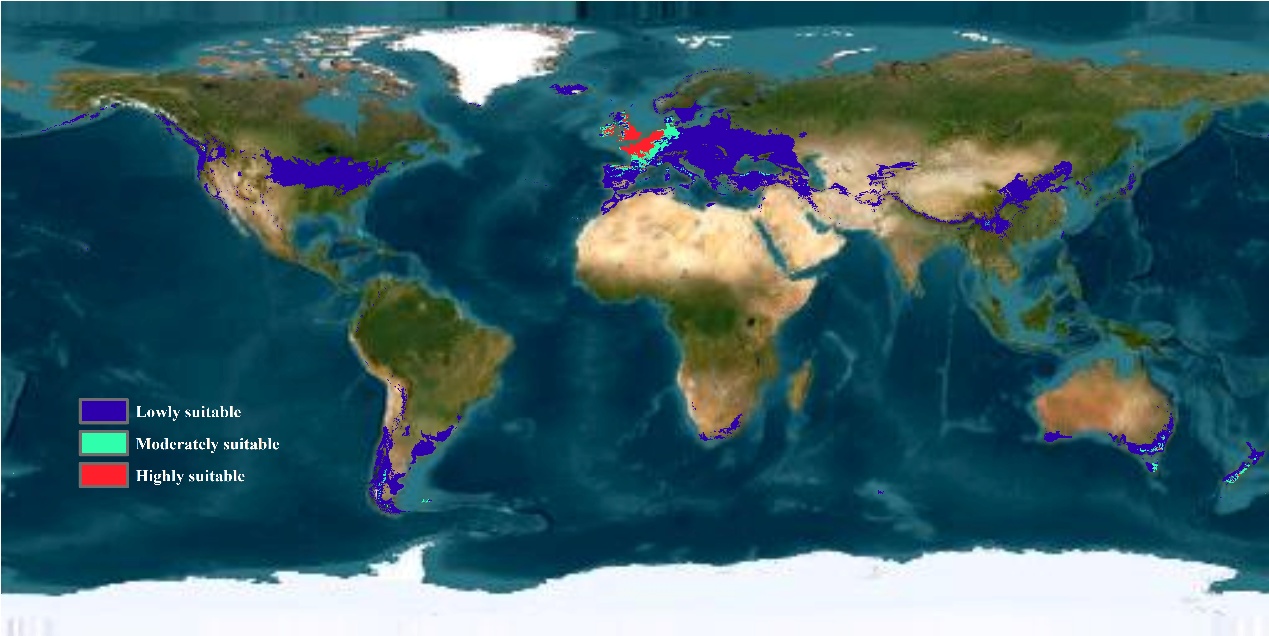

Supplement: Supplementary file 1 — Appendix S1: ece372297‐sup‐0001‐AppendixS1.zip. [file ECE3-15-e72297-s001.zip › ece372297-sup-0004-FigureS4.docx]
